# Nitro- and oxy-PAHs in grassland soils from decade-long sampling in central Europe

**DOI:** 10.1007/s10653-021-01066-y

**Published:** 2021-08-20

**Authors:** M. Wietzoreck, B. A. M. Bandowe, J. Hofman, J. Martiník, B. Nežiková, P. Kukučka, P. Přibylová, G. Lammel

**Affiliations:** 1 Max Planck Institute for Chemistry, Multiphase Chemistry Dept, Mainz, Germany; 2 Masaryk University, Research Centre for Toxic Compounds in the Environment, Brno, Czech Republic

**Keywords:** Polycyclic aromatic compounds, soil pollution, Nitrated PAHs, Soil exposure, Background, Temporal variation

## Abstract

Long-term exposure to polycyclic aromatic hydrocarbons (PAHs) and their nitrated (NPAHs) and oxygenated (OPAHs) derivatives can cause adverse health effects due to their carcinogenicity, mutagenicity and oxidative potential. The distribution of PAH derivatives in the terrestrial environment has hardly been studied, although several PAH derivatives are ubiquitous in air and long-lived in soil and water. We report the multi-annual variations in the concentrations of NPAHs, OPAHs and PAHs in soils sampled at a semi-urban (Mokrá, Czech Republic) and a regional background site (Košetice, Czech Republic) in central Europe. The concentrations of the Σ_18_NPAHs and the Σ_11+2_OPAHs and O-heterocycles were 0.31 ± 0.23 ng g^−1^ and 4.03 ± 3.03 ng g^−1^, respectively, in Košetice, while slightly higher concentrations of 0.54 ± 0.45 ng g^−1^ and 5.91 ± 0.45 ng g^−1^, respectively, were found in soil from Mokrá. Among the 5 NPAHs found in the soils, 1-nitropyrene and less so 6-nitrobenzo(a)pyrene were most abundant. The OPAHs were more evenly distributed. The ratios of the PAH derivatives to their parent PAHs in Košetice indicate that they were long-range transported to the background site. Our results show that several NPAHs and OPAHs are abundant in soil and that gas-particle partitioning is a major factor influencing the concentration of several semi-volatile NPAHs and OPAHs in the soils. Complete understanding of the long-term variations of NPAH and OPAH concentrations in soil is limited by the lack of kinetic data describing their formation and degradation.

**Supplementary Information:**

The online version contains supplementary material available at 10.1007/s10653-021-01066-y.

## Introduction

The combustion of fossil fuels and biomass is the main source of polycyclic aromatic compounds (PACs) such as polycyclic aromatic hydrocarbons (PAHs) and their nitrated (NPAHs) and oxygenated (OPAHs) derivatives (Baek et al., 1991; Bandowe & Meusel, 2017; Walgraeve et al., 2010). Besides these pyrogenic sources, PACs in contaminated soils can originate from fossil material such as coal, crude oil, petroleum, chemical waste, contaminated sewage sludge and water (Bandowe & Meusel, 2017; Bandowe et al., 2019; Vikelsøe et al., 2002). The O-heterocycle dibenzofuran could originate from the chemical industry (Nežiková et al., 2021). Another source for PAH derivatives in soils is their formation from the biodegradation and (photo)chemical oxidation of PAHs (Cerniglia, 1992; Finlayson-Pitts & Pitts, 2000; Keyte et al., 2013; Walgraeve et al., 2010). PACs in soil can enter the human body by inhalation, oral ingestion and dermal contact (Ruby et al., 2016) and can adversely affect humans as well as the ecosystem. Several PACs are carcinogenic (Collins et al., 1998; IARC, 2010, 2012) and mutagenic (Durant et al., 1996), cause oxidative stress (Bolton et al., 2000) and are endocrine-disrupting compounds (Lampi et al., 2006; Lundstedt et al., 2007; Nováková et al., 2020) and ecotoxic (Bandowe & Meusel, 2017; el Alawi et al., 2002; Sverdrup et al., 2002a, 2002b). Dibenzofuran causes effects in lung cells, contributes to oxidative stress and has estrogenic properties (Brinkmann et al., 2014; Duarte et al., 2011, 2012; Jaiswal et al., 2012), while 9-fluorenone is cytotoxic (Atsumi et al., 1998). However, these two substances were so far not found to be genotoxic or mutagenic (Leary et al., 1983; Matsumoto et al., 1988; Mortelmans et al., 1984; USEPA, 2020; Vasilieva et al., 1990). Even though several PAH derivatives can be even more toxic than the parent PAHs (Lampi et al., 2006; Lundstedt et al., 2007; WHO, 2003), the knowledge about the occurrence, cycling, fate, spatial, seasonal and long-term temporal trends of NPAHs, OPAHs and heterocyclic aromatics is limited (Lammel, 2015; Schlanges et al., 2008).

Significant portions of PACs emitted from various anthropogenic activities are transferred into soils by wet and dry deposition (Baek et al., 1991; Bandowe & Meusel, 2017) or by litter fall (Horstmann & McLachlan, 1998). Semi-volatile compounds, such as several PACs, can revolatilize from the soil to the atmosphere (Keyte et al., 2013; Lammel et al., 2009). Hence, soils are an important compartment and a major repository for the global cycling and large-scale chemodynamics of PACs (Lammel et al., 2009; Wild & Jones, 1995).

The concentration of a PAC in soil at every time-point depends on its emission intensity, formation in air and soil, degradation in air, deposition, volatilization, transport, sequestration, sorption and desorption to soil matrix, degradation in soil (biotic and abiotic), bioaccumulation, plant-uptake, formation of non-extractable residues amongst others (Idowu et al., 2019; Semple et al., 2003; Wilcke, 2000). Several of these processes were not studied in detail yet. Few studies addressed NPAHs and OPAHs in precipitation (Kawamura & Kaplan, 1983), and in fresh snow (Shahpoury et al., 2018), but no relevant model-based estimates of deposition fluxes or velocities are available. The deposition velocity depends on gas-particle partitioning in the aerosol, i.e. pollutants sorbed to particles are more efficiently deposited than gaseous, and even more so if lipophilic (Bidleman, 1988; Shahpoury et al., 2015, 2018; Škrdlíková et al., 2011). Thus, PAH derivatives might be deposited faster than their parent PAHs due to their lower vapour pressure (Tomaz et al., 2016).

The concentrations of most PACs in air and soil mainly depend on their proximity to emission sources (Bandowe et al., 2010, 2019). The highest concentrations in air and soil are found at urban and industrial sites (Arp et al., 2014; Bandowe et al., 2010, 2011, 2014; Cai et al., 2017; Lundstedt et al., 2007; Pham et al., 2015; Watanabe et al., 2005). Since PACs can undergo long-range transport due to their long lifetime in air and their vapour pressure (Keyte et al., 2013; Wilcke et al., 2014a; Wilson et al., 2020), PACs are abundant in rural and remote sites too. Despite that, the literature of PACs in soil at remote places is still limited. Some studies determined the abundance of PAHs in remote soils (Fernández et al., 2003; Marquès et al., 2017; Wang et al., 2009; Wilcke & Amelung, 2000), but the only studies about NPAHs and OPAHs in background soil samples are from Scandinavia (Brorström-Lundén et al., 2010; Vikelsøe et al., 2002), South America (Bandowe & Wilcke, 2010; Wilcke et al., 2014a), China (Bandowe et al., 2019) and the USA (Obrist et al., 2015).

PACs can also be found in subsoils due to transport by leaching, bioturbation and colloid-assisted transport (Bandowe et al., 2010; Krauss et al., 2000; Wilcke, 2000). The higher water solubility and lower lipophilicity of OPAHs than their related PAHs might render them more mobile in soils than PAHs (Lundstedt et al., 2007). Some NPAHs are less water soluble and have higher sorption coefficients (K_oc_) than their related parent PAHs, which might result in their diminished mobility in soil (Sun et al., 2017; WHO, 2003). Another major factor influencing the concentrations of PACs in soil is their formation and degradation. The half-lives of PAHs until mineralization range between days and years (Cerniglia, 1992). The biological degradation of PAHs by bacteria and fungi can result in the formation of OPAHs (Cerniglia, 1992).

There is no long-term study of PAH derivatives in soil, unlike for parent PAHs. The long-term studies of PAHs in soil found an increase in PAH abundance from 1880 to 1986 in England (Jones et al., 1989a, 1989b) and in the 1970s in Japan (Honda et al., 2007) followed by a levelling off or even a decrease in concentration thereafter (Becker et al., 2006; Cui et al., 2020; Holoubek et al., 2007b; Honda et al., 2007). In contrast, Gubler et al. (2015) only found a decreasing trend for light PAHs in Swiss soils between 1985 and 2013, while the concentration of the heavier PAHs almost stayed constant.

The aim of our study was to determine the temporal variations in the concentrations and composition profiles of OPAHs, O-heterocycles, NPAHs and PAHs in grassland soils of a central European background and a semi-urban site both located in Czech Republic. By elucidating the difference between soil at semi-urban vs rural sites and between air and soil concentration, we aim to improve the understanding of the sources, occurrence and fate of PAH derivatives in soil. We include 3-nitrobenzanthrone, a highly mutagenic nitrated oxy-PAH (Enya et al., 1997; Lübcke-von Varel et al., 2012) and less studied but abundant OPAHs and O-heterocycles, i.e. benzanthrone and 6H-benzo(c)chromen-6-one. To the best of our knowledge, this is the first study determining a time series of PAH derivatives in soil.

## Methods and materials

### Sampling

We sampled soils at grassland sites in Košetice and Mokrá, in the Czech Republic. Košetice is a rural background site located 534 m above the sea level in the central Czech Republic (85 km from Prague). The site is also a station of the European Monitoring and Evaluation Programme (EMEP), the Global Atmosphere Watch programme (GAW) and other networks. The average annual temperature (1988–2017) is 8.1 °C, and the average annual precipitation is around 650 mm. Košetice location 1 (Košetice-1) is located at the observatory on open area covered by grass. Košetice location 2 (Košetice-2) is close (28 m) to the confluence of two brooks at an open meadow. The soil samples from Košetice locations 1 and 2 were taken in summer of each year from 2010 to 2017. In this study, the soil samples from the years 2010–2017 except for year 2011 were analysed.

Mokrá is a semi-urban site at the rim of an urban and an industrial area at an elevated altitude. The site is located 13 km east–north-east of the city centre of Brno. The Brno metropolitan area has a population of ≈500,000 inhabitants (Czech Statistical Office, 2019). Mokrá location 1 (Mokrá-1) (Hostěnice Čihálky) is near a small forest that is close (40 m) to the edge of a quarry of a cement works. It is an open area covered by uncultivated grass and few small bushes. Mokrá location 2 (Mokrá-2) (Velká Baba), 3.5 km south of sampling site 1, is close to the village Sivice with approximately 1000 inhabitants (Czech Statistical Office, 2019). The sampling site is on an open grassland with a small forest 30 m to the west. To the north-west (940 m), there is a cement factory.

The soil samples from seven archived soil samples within 2006–2015 from Mokrá-1 and Mokrá-2, sampled in spring (Sp), fall (F) or summer (S), were analysed. Detailed information about the sampling dates and locations can be found in the Online Resource (Supplementary Information, SI) in Table S1. A map including both sampling locations per site is shown in Fig. S1.

A detailed description of the sampling procedure has previously been reported (Holoubek et al., 2007b, 2009). At each sampling site, the soils were inspected and the top 10 cm of the surface soil within the A horizon was sampled using a stainless steel spade (after removing the vegetation layer). At all four locations, the soil evolutions seemed identical based on visual inspection. The soil samples were all characterized as Cambisol, except at Košetice-2, where it is fluvisol. Each location was represented by a mixture of ten sub-samples collected from an area of 25 × 25 m. The samples were transported to the laboratory, air-dried at room temperature, sieved (2 mm mesh) and stored in paper bags in a dark room at constant temperature and humidity.

### Determination of soil properties

Several soil physico-chemical properties were determined on aliquots of each soil sample. The properties were measured by standard operational procedures. These include total organic carbon (TOC) content (ISO 14235, 1998), total soil nitrogen (N_tot_) (ISO 11261, 1995), soil pH_KCl_ and pH_H2O_ (ISO 10390, 2005). The basic soil properties can be found in the SI in Table S2.

### Determination of PAHs, NPAHs and OPAHs in soils

The concentrations of PAHs were determined in aliquots of the soil samples directly after sampling as described by Holoubek et al., (2007a, 2009). In brief, the soils were extracted with dichloromethane (DCM) on a Soxhlet apparatus. The soil extracts were subsequently purified on silica gel columns. PAHs in the purified soils extracts were measured by gas chromatography-mass spectrometry (GC–MS). Except for the extraction solvent, the sample preparation and the analysis of the PAHs were done similarly between the first PAH measurement (called “original” concentration) and the remeasurement taking another aliquot of the archived soil samples in May 2018. Additionally to the PAH concentration, the OPAH and NPAHs content in the soil samples was analysed as described below.

Aliquots (5 g) of the soil samples were transferred into cellulose extraction thimbles (Whatman 603 33 × 100 mm 10,350,242 and Advantec N08433X37X94mm) and placed into a Soxhlet extractor (Büchi B-811, Flawil, Switzerland). The soils were spiked with 50 µL of a standard mixture of deuterated PAHs [naphthalene-D8, phenanthrene-D10 and perylene-D12 (Dr. Ehrenstorfer, Augsburg, Germany), each with a concentration of 6.6 µg mL^−1^ in toluene] and 50 µL of a standard mixture of deuterated NPAHs [1-nitronaphthalene-D7, 2-nitrofluorene-D9, 9-nitroanthracene-D9, 3-nitrofluoranthene-D9, 1-nitropyrene-D9, 6-nitrochrysene-D11 and 6-nitrobenzo(a)pyrene-D11 (Chiron, Trondheim, Norway)], each with a concentration of 0.4 µg mL^−1^ in toluene as surrogate standards for PAHs and NPAHs, respectively. In addition, 50 µL of a deuterated OPAH standard mixture [9-fluorenone-D8 and 9,10-anthraquinone-D8 (Chiron, Trondheim, Norway)], each with a concentration of 0.8 µg mL^−1^ in ethyl acetate (EA, MS Suprasolv, Merck, Darmstadt, Germany), was spiked to the soil samples (except for the first 11 samples) to serve as surrogate standards for the OPAHs. Each sample was then extracted with 150 mL of a DCM/acetone (2:1, v:v, Rotisolv GC Ultra Grade, Roth, Karlsruhe, Germany and Suprasolv, Merck, Darmstadt, Germany) mixture for 40 min, as previously done (Klánová et al., 2008). The soil extracts were concentrated (to 1–2 mL), quantitatively transferred to an amber vial and stored until clean-up by column chromatography.

Each column was packed with 0.5 g of dried Na_2_SO_4_ and 8 g of 10% deactivated silica (Sigma Aldrich, St. Louis, MO, USA). On the top, another layer of 0.5 g of Na_2_SO_4_ was added. The column was conditioned with 6 mL of DCM followed by 6 mL of EA. The soil extract was then transferred into the column and solvent allowed to drain off. The target compounds were then eluted with 24 mL of EA followed by 24 mL of DCM collecting the eluates in vials. During the entire purification process, the column was covered with aluminium foil to avoid photodegradation of target compounds. The purified extracts were transferred into glass tubes and concentrated in an evaporation system (Turbovap II, Biotage, Uppsala, Sweden) to approximately 0.3 mL and then transferred to a GC vial. The extracts were further concentrated to 200 µL using a gentle stream of N_2_. This was followed by the addition of 50 µL nonane as a keeper, shaking and further evaporation to 50 µL. As the last step, 50 µL of a PCB121 solution (0.2 µg mL^−1^ in cyclohexane) and 50 µL of a p-terphenyl solution (4 µg mL^−1^ in toluene) were added as internal standards.

Polycyclic aromatic compounds (PACs) in the soil extracts were analysed by GC–MS in the Trace Analytical Laboratory of the research centre RECETOX at the Masaryk University in Brno, Czech Republic similar to Nežiková et al. (2021). The target compounds in this study were 27 PAHs, 17 NPAHs, 1 NOPAH, 11 OPAHs and 2 O-heterocycles. The 27 PAHs were naphthalene (NAP), acenaphthylene (ACY), acenaphthene (ACE), fluorene (FLN), phenanthrene (PHE), retene (RET), anthracene (ANT), fluoranthene (FLT), pyrene (PYR), benzo(a)anthracene (BAA), chrysene (CHR), benzo(b)fluoranthene (BBF), benzo(k)fluoranthene (BKF), benzo(a)pyrene (BAP, also called benzo(def)chrysene), indeno(1,2,3-cd)pyrene (INP), dibenz(ah)anthracene (DBA), benzo(ghi)perylene (BPE), benzo(b)fluorene (BBN), benzo(ghi)fluoranthene (BGF), cyclopenta(cd)pyrene (CCP), triphenylene (TPH), benzo(j)fluoranthene (BJF), benzo(e)pyrene (BEP), perylene (PER), dibenz(ac)anthracene (DCA), anthanthrene (ATT), coronene (COR). The target NPAHs were 1-nitronaphthalene (1-NNAP), 2-nitronaphthalene (2-NNAP), 3-nitroacenaphthene (3-NACE), 5-nitroacenaphthene (5-NACE), 2-nitrofluorene (2-NFLN), 9-nitroanthracene (9-NANT), 9-nitrophenanthrene (9-NPHE), 3-nitrophenanthrene (3-NPHE), 2-nitrofluoranthene (2-NFLT), 3-nitrofluoranthene (3-NFLT), reported as sum (2- + 3-NFLT), 1-nitropyrene (1-NPYR), 7-nitrobenzo(a)anthracene (7-NBAA), 6-nitrochrysene (6-NCHR), 1,3-dinitropyrene (1,3-N_2_PYR), 1,6-dinitropyrene (1,6-N_2_PYR), 1,8-dinitropyrene (1,8-N_2_PYR) and 6-nitrobenzo(a)pyrene (6-NBAP). In addition, the NOPAH 3-nitrobenzanthrone (3-NBAN) was another target compound. The OPAHs were 1,4-naphthoquinone (1,4-O_2_NAP), naphthalene-1-aldehyde (1-(CHO)NAP), 9H-fluoren-9-one (9-OFLN), 9,10-anthraquinone (9,10-O_2_ANT), 11H-benzo(a)fluoren-11-one (11-OBaFLN), 11H-benzo(b)fluoren-11-one (11-OBbFLN), benzanthrone (7H-benz(de)anthracene-7-one) (BAN), benz(a)anthracene-7,12-dione (7,12-O_2_BAA), 5,12-naphthacenequinone (5,12-O_2_NAC) and 6H-benzo(cd)pyren-6-one (6-OBPYR), while O-heterocycles were dibenzofuran (DBF) and 6H-benzo(c)chromen-6-one (6-OBCC, also called 6H-dibenzo(bd)pyran-6-one). All targeted compounds including their physico-chemical properties are shown in Table S3.

PAHs were measured on a GC (GC 7890A Agilent Technologies, Santa Clara, USA) using a 60 m × 0.25 mm × 0.25 µm Rxi-5Sil MS column (Restek, Bellefonte, USA). The instrument was coupled to a triple quadrupole mass spectrometer (MS 7000B, Agilent Technologies, Santa Clara, USA). The GC temperature programme started at 80 °C (hold for 1 min) followed by an increase by 15 °C min^−1^ to 180 °C and by 5 °C min^−1^ to 310 °C, which was held for 20 min. The injection volume was 1 µL in splitless mode at 280 °C. As carrier gas, helium with a flow rate of 1.5 mL min^−1^ was used. The transfer line and the ion source were set to 310 °C and 320 °C, respectively. Electron ionization (EI) in positive mode was applied as the ionization technique. Selected ion monitoring (SIM) mode was applied using one ion for quantification and one or two ions per compound for qualification. The retention times as well as the quantifying ions of the targeted PAHs are shown in Table S4. For the evaluation, the internal standard method was used, calculating the ratio of the internal standard p-terphenyl and the target compound.

All nitrated and oxygenated PAHs were analysed by GC–MS using atmospheric pressure chemical ionization (APCI) in negative mode on a 7890 GC (Agilent Technologies, Santa Clara, USA) coupled to a triple quadrupole MS Xevo TQ-S (Waters, Milford, USA). A 30 m × 0.25 mm × 0.25 µm Rxi-5Sil MS column (Restek, Bellefonte, USA) was used. One microlitre of the samples was injected splitless at 270 °C. Helium was used as the carrier gas at a constant flow of 1.5 mL min^−1^. The oven temperature program was starting at 90 °C for 1 min followed by an increase of 40 °C min^−1^ to 180 °C and 5 °C min^−1^ to 320 °C (6 min hold). The target compounds were measured in the multiple reaction monitoring (MRM) mode. The MRM m/z ratios and the retention time of the targeted OPAHs and NPAHs are given in Table S5. The ratio of the target compound and the internal standard PCB121 was used to determine the target compound concentration.

### Quality control

Details about the quality control such as the limits of quantification (LOQs), the recovery correction and the repeatability of the method can be found in the SI (Chapter S1). All reported values are blank corrected by using five method blanks (undergone whole procedure but without any soil). The NPAH concentrations are recovery-corrected since the variability of the recoveries of the deuterated NPAHs was relatively high. In contrast, the PAHs (except for the low molecular weight PAHs in three samples due to accidentally evaporation to almost dryness) and OPAHs are not recovery-corrected due to lower variability and higher values of the recoveries. The coefficient of variation of the deuterated PAHs and OPAHs was 15–33%, while it was 32–73% for the NPAHs.

The average recoveries of the deuterated PAHs (NAP-D8, PHE-D10 and PER-D12) in the samples and blanks were 37 ± 17%, 75 ± 12% and 102 ± 16%, respectively. The average recoveries of 9-OFLN-D8 and 9,10-O_2_ANT-D9 in the samples and blanks were 101% ± 34% and 107% ± 33%, respectively. The average recoveries of 1-NNAP-D7, 2-NFLN-D9, 9-NANT-D9, 3-NFLT-D9, 1-NPYR-D9, 6-NCHR-D11 and 6-NBAP-D11 in the samples and blanks were 15 ± 10%, 78 ± 25%, 55 ± 34%, 41 ± 23%, 32 ± 24%, 31 ± 19% and 43 ± 47%, respectively. More details about the recoveries are given in the SI in Chapter S1.3. The instrumental LOQs (iLOQs) ranged between 0.020–0.107 ng g^−1^, 0.001–0.026 ng g^−1^ (except 9,10-phenanthrenequinone with 2.844 ng g^−1^) and 0.001–0.488 for the PAHs, OPAHs + O-heterocycles and NPAHs, respectively. The individual LOQs including the method LOQs (mLOQs) are shown in Table S7.

## Results and discussion

### PAHs

#### Comments on preservation of PAHs during storage

Polycyclic aromatic compounds (PACs) in sampled soils are subject to contamination from the laboratory environment and processes such as volatilization, degradation (microbial, thermal and photodegradation) and formation of non-extractable residues, which might alter their concentrations if the soils are not properly protected, pre-treated and stored under the right conditions. Air-drying and storage of the archived soil samples in closed paper bags in a dark room at ambient temperature and constant relative humidity are considered being adequate for the preservation of PAH components of soils and has been adopted in the preservation and archiving in other studies (Bandowe et al., 2014; Jones et al., 1989a, 1989b).

In order to verify that the composition of PACs in the soil has been preserved during the (between 3 and almost 12 years), we compared the concentrations of the ∑_16_PAHs measured in this study (“archived samples”) to the concentrations measured in the same samples before archiving (“original”). Based on the strong correlations (Košetice: Pearson correlation coefficient *r* = 0.85, *p* < 0.01; Mokrá, in brackets without 2 outliers: *r* = 0.47 (0.90), *p* = 0.06 (< 0.01)) between the concentrations determined before storage and after storage (archived samples), we conclude that the PAHs composition in soils has remained uncontaminated and stable (preserved) during long years of storage. More information can be found in the Online Resource (Supplementary Information, SI) (Chapter S2).

#### Concentration of PAH in soils

**Fig. 1:**
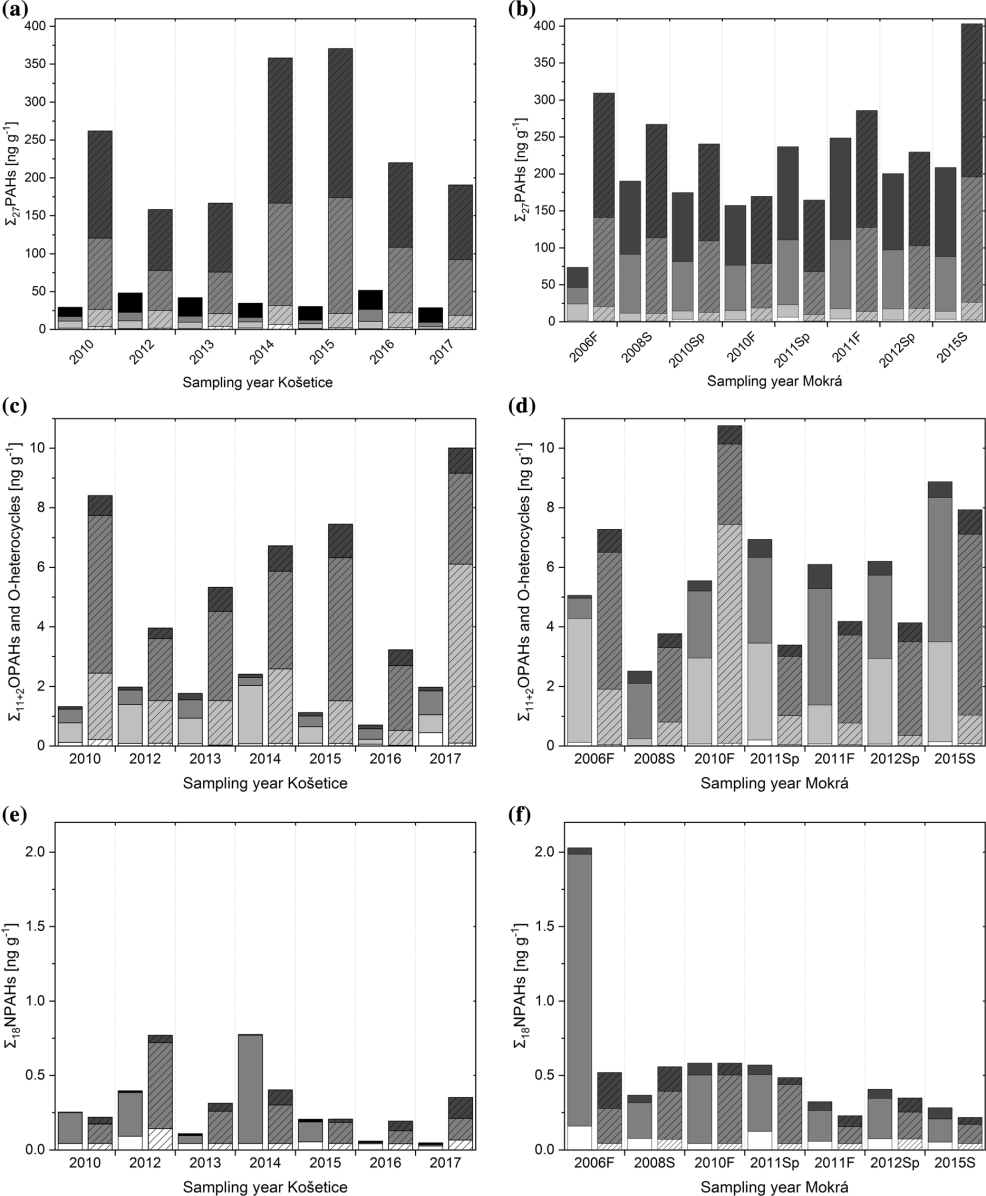
Concentration of **a**, **b** Σ_27_PAHs split into 2-ring (white), 3-ring (light grey), 4-ring (grey) and 5–7-ring PAHs (dark grey), **a** at Košetice-1 (plain) and Košetice-2 (dashed); **b** at Mokrá-1 (plain) and Mokrá-2 (dashed); **c**, **d** Σ_11+2_OPAHs and O-heterocycles split into 2-ring (white), 3-ring (light grey), 4-ring (grey) and 5-ring OPAHs (dark grey), **c** at Košetice-1 (plain) and Košetice-2 (dashed); **d** at Mokrá-1 (plain) and Mokrá-2 (dashed); **e**, **f** Σ_18_NPAHs split into 2-ring NPAHs (white), 3-ring NPAHs (light grey), 4-ring NPAHs (grey) and 5-ring NPAHs (dark grey), **e** at Košetice-1 (plain) and Košetice-2 (dashed); **f** at Mokrá-1 (plain) and Mokrá-2 (dashed); (F, fall; Sp, spring; S, Summer). Concentrations normalized to TOC content shown in Fig. S4 in the SI

We found 26 PAHs in over 75% of the examined soil samples from Košetice and Mokrá, while CCP could only be quantified in one soil sample. The detection frequencies of the PAHs are shown in the SI, Fig. S3a. Except for CCP, the detection frequencies of PAHs were > 75% with higher MW PAHs even > 90%. Figure 1a, b illustrates the concentration of ∑_27_PAHs, disaggregated for number of rings and examined years. The same plot but with the concentrations normalized to the total organic carbon (TOC) content (Table S2) is shown in Fig. S4. Figure S5 in the SI illustrates the location averaged concentrations shown as box plots. The concentrations of the sum of 16 EPA-prioritized PAHs (∑_16_PAHs) as well as the concentrations normalized to the TOC content are shown in the SI in Tables S9 and S10, and the concentrations of the individual PAHs in Table S11.

As summarized by Wilcke (2000), the soil properties influence the concentration and distribution of PAHs in soil. The basic soil properties can be found in the SI in Table S2. The locations mainly differ by the TOC content. The average TOC content is 1.9 ± 0.5%, 5.2 ± 0.7%, 3.7 ± 1.5% and 1.8 ± 0.6% at Košetice-1, Košetice-2, Mokrá-1 and Mokrá-2, respectively. The influence of the TOC content on the concentration of PAHs and PAH derivatives in soil will be described in the following as well as in “OPAHs and O-heterocycles” and “NPAHs” sections. In addition, Wilcke summarizes the influence of vegetation, land use and aggregate surface on the PAH concentration. However, these factors are not crucial for the comparison of the four locations in this study since these soil properties are similar or at least comparable between the four locations.

The concentration of the ∑_27_PAHs is 30 and 26% higher than the ∑_16_PAHs in Košetice and Mokrá, respectively. Our study reveals that several PAHs, which are not included in the list of traditionally measured 16 EPA-PAHs, showed higher concentrations than some of the 16 EPA-PAHs in these central European background and semi-urban soils. Many of these PAHs could contribute significantly to the overall risk posed by organic pollutants in the soils and are of value for determining the sources of PAHs (Andersson & Achten, 2015; Dvorská et al., 2012; Richter-Brockmann & Achten, 2018). For example, we found BJF and ATT with known carcinogenicity and toxicity equivalence factors (TEFs) of 0.1, respectively, in all examined soil samples (Greim, 2008).

The mean concentrations of the Σ_27_PAHs at the locations in Košetice and Mokrá are 142 ± 124 (29–370) ng g^−1^ and 223 ± 75 (74–403) ng g^−1^, respectively. The PAH levels found are at the lower end of the range spanned by other rural sites in Europe and comparable with levels reported from background sites in Northern Europe (Table S12). When studying the spatial variation of the samples from this study, it has to be considered that the sampling years between the samples from Mokrá are not totally matching the sampling years from the Košetice soil. Nevertheless, we can conclude that the mean concentration of the Σ_27_PAHs in Mokrá soils is statistically significantly higher than in Košetice soils (*p* < 0.05, Student’s *t*-test). We assume that the significant difference between the PAH concentrations of both sites is caused by the higher influence of anthropogenic emission sources at Mokrá due to higher proximity to urban and industrial areas. The greater contribution of high molecular weight (MW) PAHs in near source locations, such as Mokrá in our study, compared to rural background sites was reported before (International POPs Elimination Project—IPEP, 2006; Nam et al., 2008).

The concentration of Σ_27_PAHs at Mokrá-1 is lower, but not significantly (*p* = 0.051, Student’s *t*-test), than at Mokrá-2, i.e. average: 186 ± 54 (range: 74–249) ng g^−1^ and 259 ± 78 (165–403) ng g^−1^, respectively. The average concentration of Σ_27_PAHs at Košetice-2 (247 ± 88 (158–370) ng g^−1^) is in the same range as in both locations in Mokrá (Fig. S5a) but significantly higher (*p* < 0.01, Student’s *t*-test) than at Košetice-1 (38 ± 9 (29–52) ng g^−1^). The concentrations of PAHs in our studied soils significantly correlated (∑_27_PAHs, *r* = 0.39, *p* < 0.05) with the TOC content, a finding which was also reported in earlier findings (Cai et al., 2017; Holoubek et al., 2009; Wilcke & Amelung, 2000). Our finding suggests that variations in organic matter levels might partly explain the spatial and temporal variations of the PAH levels in the sampled soils. Organic matter in soil is the main sorbent for PAHs and hence ultimately drives the amount of PAHs that are partitioned into soil from diffusely contaminated atmosphere (if the concentration of PAHs is in equilibrium with the concentration in soil; Wilcke & Amelung, 2000). Normalization of the PAH concentration with the soil organic carbon concentrations (Fig. S5d and Table S10), did not completely remove the differences in concentrations between the Mokrá and Košetice soils. We therefore conclude that variability of soil organic carbon cannot completely explain the observed temporal and spatial differences. The temporal variation is discussed in “Temporal variations of PACs in soil” section and in Chapter S3 in the SI considering soil samples since 1996.

### OPAHs and O-heterocycles

Out of the targeted 11 oxygenated PAHs (OPAHs) and 2 O-heterocycles, 10 OPAHs and both O-heterocycles were found in soils of Košetice and Mokrá. Only 9,10-O_2_PHE was not detected in any sample. This has to be interpreted considering the relatively high LOQ of 9,10-O_2_PHE (see Table S7b in the SI). The detection frequencies of the PAH derivatives are shown in the SI, Fig. S3b. The detection frequencies of high MW OPAHs (≥ 4-ring OPAHs) were > 90%, while the lower MW OPAHs and O-heterocycles had a detection frequency of more than 75%, except for 6-OBCC (58%), 9,10-O_2_ANT (23%) and 9,10-O_2_PHE (0%). Regarding the low detection frequencies of 9,10-O_2_ANT, the high and varying amount in the blanks leading to a high mLOQ of 9,10-O_2_ANT has to be considered.

The concentrations of the sum of 11 OPAHs and the 2 O-heterocycles (∑_11+2_OPAHs and O-heterocycles) are shown in Fig. 1c, d. The TOC normalized concentrations and the results of the individual compounds are given in Fig. S4 and in Tables S9, S10 and S13 in the SI.

The averaged concentration of the Σ_11+2_OPAHs and O-heterocycles in Košetice (4.07 ± 3.08 ng g^−1^) is lower (*p* = 0.076, Student’s *t*-test) than in Mokrá (5.91 ± 2.30 ng g^−1^). The TOC normalized concentrations are even significantly different (*p* < 0.05, Student’s *t*-test) between the two sites (see Fig. S5b, e) showing that the difference is not caused by the influence of the soil TOC content. As mentioned in “Concentration of PAH in soils” section, it should be considered that spatial and temporal differences are conflated when comparing the average concentrations between both sites since the sampling years differ between soil samples from Košetice and Mokrá. The temporal variation (coefficient of variation) of the concentrations at each location is between 31 and 48%. Nevertheless, the higher OPAH burden at Mokrá is significant and might be caused by the higher proximity to emission sources showing the importance of primary emitted OPAHs on the soil pollution. The average concentration of the Σ_11+2_OPAHs and O-heterocycles at Košetice-1 (1.61 ± 0.59 ng g^−1^) is significantly lower (*p* < 0.01, Student’s *t*-test) than the concentrations at Košetice-2, Mokrá-1 and Mokrá-2 (6.54 ± 2.48 ng g^−1^, 5.89 ± 1.93 ng g^−1^ and 5.92 ± 2.79 ng g^−1^). The difference of the concentrations between the two locations in Košetice can mainly be explained by the TOC content. The average TOC content of Košetice-1 is 1.9%, while it is 5.0% at Košetice-2. Instead of 600% when comparing the concentrations per mass of soil, the average TOC content normalized concentration at Košetice-2 is only 60% higher than at Košetice-1.

We found a correlation (*r* = 0.55, *p* < 0.01) of the high MW (4–5-ring) OPAHs and O-heterocycles with the TOC content in soil, but no significant correlation of the low MW (2–3-ring) OPAHs and O-heterocycles (*r* = 0.13, *p* = 0.52). The determined correlation combines temporal and spatial correlation. Wilcke et al. (2014a) found a correlation of the OPAHs to the TOC content at spatial scale. The lack of significant correlation between the TOC content and the concentrations of 2–3-ring OPAHs over the spatial and temporal scales covered by our soils might be due their higher mobility, degradability and formation in soil (Table S3, Wilcke et al., 2014b). At the site Košetice, the correlation of TOC with high MW OPAHs is 0.92 (*p* < 0.01), but the correlation was not statistically significant (*r* = 0.38, *p* = 0.18) in the Mokrá soils. The lack of correlation at Mokrá is because close to sources, spatial distribution of the OPAHs in soil will be more influenced by the intensity of input from primary sources and less influenced by the soil’s spatial heterogeneity of soil property such as organic matter content. Apart from Wilcke et al. (2014a), Bandowe et al. (2014) also found a correlation of the soil organic carbon content with the concentration of several PAHs and OPAHs for spatial differences, whereas others (Bandowe et al., 2011; Cai et al., 2017; Sun et al., 2017) did not.

Apart from the TOC content, other characteristics of the locations may have influenced soil burdens. In contrast to Košetice-1 with no major influence other than OPAHs from the air by wet and dry deposition and from formation in soil, Košetice-2 is partly surrounded by trees at the confluence of two brooks resulting in a more diverse impact on the PAC levels. Soils in river valleys or flooded areas often contain higher amounts of PAHs due to the accumulation of river sediment with high organic matter (Wilcke, 2000).

Only few studies addressed OPAHs in soil at semi-urban and/or background sites (Table 1). Brorström-Lundén et al. (2010) measured 10 OPAHs in background and urban soil samples from Sweden. The concentrations of 9-OFLN, 9,10-O_2_ANT, 7,12-O_2_BAA and 6-OBPYR in the Swedish background soil samples were higher than in the soil samples from this study. However, specific information about the land use is not available, making a comparison more difficult since the PAC concentration is strongly influenced by land use and vegetation (Bandowe et al., 2019). One study with lower OPAH levels than in soil from Košetice and Mokrá is from grassland and scrubland soil samples in Argentina (Wilcke et al., 2014a). This can be explained by the overall low pollution of the investigated soil due to lower anthropogenic influence compared to samples from Europe. Slightly higher OPAH concentrations compared to this study were found in agricultural and remote forest soil samples (Obrist et al., 2015; Sun et al., 2017). Direct comparison is not possible due to pollution situation and land-use type. Bandowe and Wilcke (2010) measured OPAHs in rural tropical forest soil, Amazonia, Brazil. There, concentrations of 1-(CHO)NAP, 9-OFLN and 9,10-O_2_ANT were over one order of magnitude higher than in Mokrá and Košetice, despite much lower PAH burden compared to our samples. However, these samples are not directly comparable because of differing land-use type (grassland vs forest, forest filter effect). Tropical regions might also exhibit higher formation rates of OPAHs from enhanced photochemical, thermal and microbial degradation of PAHs (higher in the inner tropics, and also in subtropical China; Bandowe et al., 2014, 2019). The soil temperature and higher microbial activity may explain more efficient formation of soil OPAH from soil PAH, and faster degradation of PAHs, hence, higher c_OPAH_/c_PAH_ in warmer soils (Bandowe et al., 2014).

**Table 1:** OPAH concentration in surface soil (ng g^−1^; sampling depth 10 cm) at different locations; ND = not determined; < *x* = smaller than LOQ but LOQ unknown

Location	Košetice, Czech Republic^c^	Mokrá, Czech Republic^c^	North of Manaus, Brazil^d^	Gardsjön, Sweden^a,e^	20 sites in Argentina^f^	Eastern China^b, g^	China plateau^h^	China temperate^h^	China subtropical^h^	China tropical^h^
Type of location	Background	Semi-urban	Background	Background	Remote	Agricultural	Rural			
Land use	Grassland	Grassland	Forest	Not specified	Grassland/scrubland	Agricultural	Forest, agricultural, river shore, grassland			
Number of OPAHs	11	11	7	10	15	4	15	15	15	15
ΣOPAHs	4.1	5.9	6.6	112	0.1–125	9	123	76	147	70
1,4-O_2_NAP	0.13	0.05	< 1	ND	< *x*	ND	0.5	0.5	1	0.5
1-(CHO)NAP	0.02	0.02	1.1	ND	< *x*	ND	0.7	0.8	1.9	0.6
9-OFLN	0.57	0.70	1.7	3.6	< *x* − 2.8	3.7	4.5	9.4	13.4	5.0
9,10-O_2_ANT	0.32	0.85	2.1	13	< *x* − 8.6	7.1	4.8	12.2	26.2	3.6
9,10-O_2_PHE	< 2.8	< 2.8	ND	ND	ND	ND	ND	ND	ND	ND
11-OBaFLN	0.52	0.94	ND	ND	< *x* − 5.2	ND	4.3	4.8	7.0	1.1
11-OBbFLN	0.56	0.92	ND	ND	ND	ND	ND	ND	ND	ND
BAN	0.27	0.40	ND	ND	< *x* − 16	6.1	51	12.6	33.4	17.5
7,12-O_2_BAA	0.38	0.49	ND	28	< *x* − 20	2.9	39	9.4	19.6	5.9
5,12-O_2_NAC	0.21	0.33	ND	ND	< *x* − 2.4	ND	4.5	3.2	6.2	6.9
6-OBPYR	0.43	0.53	ND	31	< *x* − 21	ND	9.6	9.1	15.1	12.6
DBF	0.37	0.37	ND	< 3	ND	ND	ND	ND	ND	ND
6-OBCC	0.28	0.29	ND	ND	ND	ND	ND	ND	ND	ND
TOC [g kg^−1^]	37	28	41	ND	4–40	ND	39	19	19	21

^a^Sampling depth: upper 2–3 cm

^b^Sampling depth: 0–20 cm

^c^This study

^d^Bandowe and Wilcke (2010)

^e^Brorström-Lundén et al., (2010)

^f^Wilcke et al., (2014a)

^g^Sun et al., (2017)

^h^Bandowe et al., (2019)

In this study, the O-heterocycle DBF was determined with a concentration of 0.2–0.5 ng g^−1^. Brorström-Lundén et al. (2010) could not quantify DBF in Swedish background soil because it was lower than the LOQ. Since the LOQ was with 3 ng g^−1^ significantly higher than the examined concentrations in this study, a comparison is not possible for the background soil. However, the concentration of DBF in the least polluted urban soil sample, Göteborg, was slightly higher (0.94 ng g^−1^).

#### Composition pattern of OPAHs

**Fig. 2:**
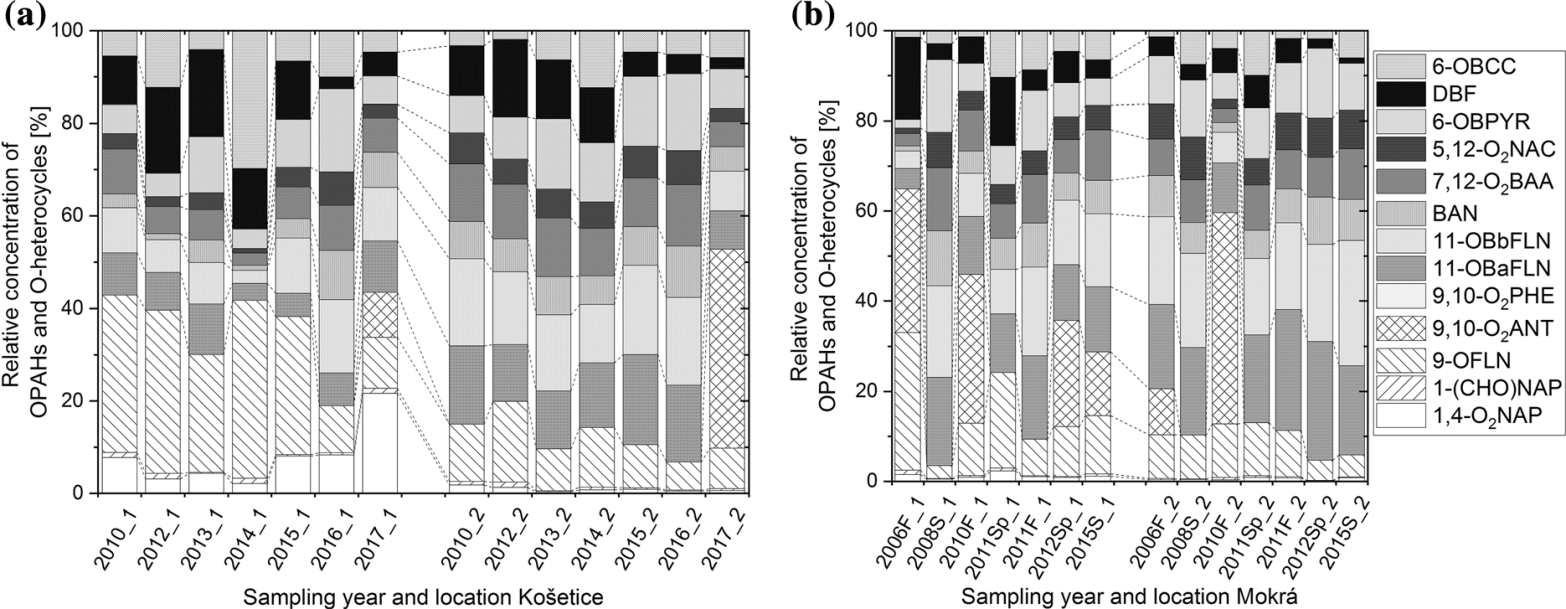
Composition pattern of OPAHs and O-heterocycles **a** in Košetice **b** in Mokrá (F, fall; Sp, spring; S, summer). Absolute data available in Table S9. Values < LOQ were replaced by LOQ/2 if the detection frequency was > 25% (Fig. S3b), else replaced by 0 ng g^−1^

The composition patterns of OPAHs and O-heterocycles of each year and location can be found in Fig. 2. Figure S6b in the SI shows the location average composition patterns. 9-OFLN, 11-OBaFLN and 11-OBbFLN are the most abundant OPAHs. Some differences in the pattern might result from 9,10-O_2_ANT, which has a low detection frequency and a relatively high LOQ (Table S7b). Concentrations < LOQ of substance with a detection frequency < 25% are replaced by 0 ng g^−1^, substances detected more often by LOQ/2. Thus, the relative contribution of 9,10-O_2_ANT to the total OPAHs can be relatively high if found > LOQ but is not reflected if concentration was < LOQ. At Košetice, DBF and 9-OFLN significantly contributed to the burden of OPAHs and O-heterocycles. The relative contribution of 9-OFLN and DBF to the ΣOPAHs and O-heterocycles was even higher in air samples (Nežiková et al., 2021) than in soil samples from Košetice (Figure S7b). Nežiková and colleagues explained the high relative concentrations of DBF by its high atmospheric lifetime compared to other PACs (Brubaker & Hites, 1998). In addition, we assume a slower deposition rate of DBF compared to other OPAHs or O-heterocycles since it is almost completely in the gas phase (Nežiková et al., 2021). Similar processes can be assumed for 9-OFLN. The estimated atmospheric lifetime of 9-OFLN is slightly shorter than of DBF, but longer than of all targeted 4-ring OPAHs (USEPA, 2019). Ding et al. (2019) even calculated an atmospheric lifetime of 9.7 days for 9-OFLN. DBF is found in coal tar as well as coal and wood tar creosotes. Furthermore, it is used in heat-transfer oils, as a carrier for dyeing and printing textiles, and as an antioxidant in plastics (PubChem, 2021a). 9-OFLN is used as an intermediate and as a reagent in the industry (PubChem, 2021b). Similar to other PACs, 9-OFLN and DBF are formed during the incomplete combustion of biomass and fossil fuels (PubChem, 2021a, 2021b). Since the dominance of 9-OFLN is widespread (see Table 1), we assume that the pollutant is not from a specific source but from combustion sources and secondary formation.

The samples from 2016 in Košetice at both locations and from 2008 at Mokrá-1 differ from the other samples at the same locations by their low concentrations of 9-OFLN and DBF. This is also the case for the 2–3-ring PAHs, as shown in the annual pattern of different ring size PAHs in Fig. S8 in the SI. The low contribution of the low MW OPAHs, especially 9-OFLN, strongly affects the absolute OPAH concentrations (Σ_11+2_OPAHs and O-heterocycles) leading to the lowest values compared to the other years at each location (see Fig. 1). We hypothesize that this is caused by the weather conditions. Since it was shown before that the highest OPAH air concentrations are in the cold season due to heating (Nežiková et al., 2021), we focussed on the winter months December, January and February. It can be expected that the pollution from winter is still detected in the soil sampled in summer due to the high persistence of the targeted compounds in soil. Wild et al. (1991) determined a lifetime for PAHs of approximately 2 (NAP) to 9 years (BPE) in sewage sludge amended on soil. Kuśmierz et al. (2016) found slightly smaller half-lives, which still ranged 141–1165 d for 3-ring and 6-ring PAHs, respectively. Doick et al. (2005) found comparable values for BAP but smaller half-lives for FLT. According to Wild and Jones (1995), leaching is not a crucial factor for the loss of PAHs due to their low water solubility and high affinity to soil organic matter. Lundstedt et al. (2003) measured the percentage of residual soil pollutants after 29 days. While 90% of the initial concentration of 3-ring PAHs was degraded, 5–6 ring PAHs were not significantly degraded. Lundstedt et al. (2003) concluded that the OPAHs either degraded slower than the PAHs or that they get formed during storage since the residual amount of the OPAHs was generally higher than of the parent PAHs. Based on estimates (BioWin4 of EPI-Suite, USEPA, 2019; Table S3), the biodegradability of OPAHs is similar or slightly higher than of their parent PAHs. Obviously, more research is needed in this field.

The air temperature in winter 2015/2016 was the second highest in the studied period 2006–2017 (Table S14) resulting in a higher percentage of semi-volatile OPAHs in the gas phase. The higher share of OPAHs in the gas phase leads to a lower amount of deposited pollutants, since wet and dry deposition of gaseous substances is much less efficient than of substances in the particulate fraction (Bidleman, 1988; Shahpoury et al., 2015; Škrdlíková et al., 2011). Elevated concentrations in air of the examined OPAHs can be confirmed by data from Nežiková et al. (2021). They found significantly higher concentration of DBF in Košetice in winter 2015/2016 compared to the other years. In addition, the winter-to-summer ratio in 2016 was very high compared to 2015 and 2017 for DBF but also for almost all other OPAHs. The influence of the different gas-particle partitioning due to the temperature on the concentration in soil is only significant for compounds showing a high sensitivity of temperature on their particulate fraction. The deposition flux of low MW OPAHs such as 1-NAP(CHO) and 1,4-O_2_NAP, as well as high MW OPAHs such as 11-OBaFLN, 11-OBbFLN, BAN, 7,12-O_2_BAA and 5,12-O_2_NAC will not change significantly with temperature since these compounds will stay in the gaseous phase (low MW OPAHs) or the particulate phase (high MW OPAHs), respectively (Lammel et al., 2020; Nežiková et al., 2021; Tomaz et al., 2016).

The lower than average precipitation in winter 2015/2016 (Table S14) can be another reason for the low amount of OPAHs in the soil samples from 2006. The amount and type of precipitation will have a significant influence for all OPAHs in the particulate fraction. High amounts of precipitation, especially of snow, an efficient scavenger of particulate matter (Shahpoury et al., 2018), lead to high OPAH concentrations in soil. The winter 2007/2008 was warmer and dryer than average (Table S14), which could be the reason for the low concentration of low MW OPAHs in soil from Mokrá in 2008. The opposite was found in 2006 and 2010. In these years, the absolute OPAH concentrations and the contributions of low MW PAHs and OPAHs are high. This can be explained by low temperatures and high precipitation in the respective winters (Table S14), leading to a high amount of deposited particulate phase pollutants.

When comparing concentrations in air and soil, it has to be considered that the air concentration is composed of gas and particulate phase air pollutants, while the concentration in soil is mainly discriminating against the gas phase PACs, as dry deposition velocity of lipophilic trace gases is almost negligible (Atlas & Giam, 1988; Bidleman, 1988). It has to be considered that processes in air, especially the gas-particle partitioning can significantly influence the concentrations in soil due to atmospheric deposition. The substance pattern in air is preserved in soil if degradability in soil and runoff mass fluxes are much smaller than the deposition flux or if similar across substances provided that the deposition flux is similar for all substances. The Pearson correlation coefficient of the relative contribution of OPAHs in soil with air at Košetice-1 (2015–2017; Nežiková et al., 2021) was 0.15 (*p* = 0.64). The pattern mainly differed by the higher contribution of 9-OFLN and DBF in air (Fig. S7). Hence, ignoring the contributions of DBF and 9-OFLN, the correlation coefficient of the contributions between air and soil at Košetice-1 is 0.66 (*p* < 0.05). There is no indication for a particularly efficient degradation or leachability of DBF and 9-OFLN in soil (Table S3) that could explain the high difference. Thus, we hypothesize that the difference in contribution is caused by the gas-particle partitioning of these low MW OPAHs, which have a small particulate fraction, even in winter (particulate fraction of all seasons ≤ 0.01 and 0.04 ± 0.05 for DBF and 9-OFLN, respectively; Nežiková et al., 2021). Since the gaseous lipophilic pollutants are not efficiently deposited by wet and dry deposition (Bidleman, 1988; Shahpoury et al., 2015; Škrdlíková et al., 2011), their contribution in air is much higher than in soil. The same is found for the 3-ring PAHs FLN and PHE (Fig. S7). The particulate mass fractions of FLN and PHE range 0.06 ± 0.04 at Košetice (Degrendele et al., 2020; Lammel et al., 2010; Nežiková et al., 2021) and at rural sites and towns in the area of Mokrá (Landlová et al., 2014), similar to 9-OFLN and DBF.

#### OPAH/PAH ratios

Compared to findings of OPAHs in soil at other background/rural sites, the OPAH/PAH ratios at Mokrá and Košetice (Fig. S9) are among the lowest ever reported (Table S15**)**. The contributing influences are difficult to reveal, as the ratios result from the combined influences of primary emission patterns, lifetimes in air and soil, and the efficiency of deposition and of conversion in air (along the transport from primary sources) and soil. Most of the related kinetic data are not available in literature yet. However, when comparing the soil ratios to the ratios in air at the same location (taken from Nežiková et al., 2021), the uncertainty of different primary emission patterns and of the formation and degradation in air can be ruled out. The air ratios are illustrated in Fig. S9b. The measured air concentration can be compared to the soil concentrations of the same period at Košetice-1 since the air was sampled at the very same location at the Košetice observatory. The high ratio of 1,4-O_2_NAP and NAP in soil at Košetice-1 could be partly explained by a high ratio in air, possibly due to strong secondary formation of 1,4-O_2_NAP by atmospheric oxidants (Lu et al., 2005). The higher ratio in soil compared to air could indicate formation of 1,4-O_2_NAP in soil (Hadibarata et al., 2012). However, the difference is not significant due to the high variability of the ratio, which is mainly based on a low detection frequency in the air samples. The mobility and the biodegradability cannot be a cause for the higher ratio in soil compared to air since the mobility of NAP is lower than of 1,4-O_2_NAP and their estimated biodegradabilities are almost similar (USEPA, 2019, see Table S3). However, the higher particulate fraction of 1,4-O_2_NAP compared to NAP (Nežiková et al., 2021) could lead to a significantly higher percentage of deposited 1,4-O_2_NAP compared to NAP. Furthermore, the ratio can also be influenced by the higher vapour pressure of NAP resulting in a higher revolatilization rate of NAP.

The higher particulate fraction of 9-OFLN compared to FLN might be a reason for the higher ratio of 9-OFLN/FLN in soil compared to air due to higher deposition flux of the particulate phase 9-OFLN. The mobility and biodegradability in soil can be neglected due to similar biodegradability and even higher mobility of 9-OFLN in soil (Table S3). Additionally, we hypothesize that the formation of 9-OFLN in soil from microbial transformation of FLN contributes to the higher ratio of 9-OFLN/FLN in soil compared to the ratio in air (George & Neufeld, 1989; Wilcke et al., 2014b). Since BBN was not measured by Nežiková et al. (2021) in air, the ratio 11-OBbFLN/BBN is not available for air in Košetice. The lower ratio of 7,12-O_2_BAA/BAA in soil compared to air cannot be caused by the difference in gas-particle partitioning, since the particulate mass fraction of 7,12-O_2_BAA is higher (Nežiková et al., 2021), which would lead to higher ratios in soil. The lower ratio could indicate that the quinone is degraded faster and/or is more mobile in soil than the parent PAH, supported by the respective estimates of physico-chemical parameters (Table S3) and by degradability studies (Matscheko et al., 2002). Possibly, 7,12-O_2_BAA is not or only with a low percentage formed in soil, because the bacteria mainly attack the bay region at the 1,2-position as well as the 8,9- and 10,11-position (Gibson et al., 1975; Jerina et al., 1984; Mahaffey et al., 1988; Schneider et al., 1996) to form dihydrodiols and aromatic acids. However, Moody et al. (2005) reported the formation of 7,12-O_2_BAA from one bacterial strain along with several other degradation products. Wu et al. (2010) reported the formation of 7,12-O_2_BAA in fungi from sediments and Cajthaml et al. (2006) by a ligninolytic fungus. We cannot draw conclusions from the 9,10-O_2_ANT/ANT ratio since 9,10-O_2_ANT was < LOQ in most soil samples, except 2017 at Košetice-1. Nevertheless, it is known from literature that, like 7,12-O_2_BAA, another position than the 9- and 10-positions of ANT is preferably attacked by bacteria and in the metabolization from mammals to form dihydroxyanthracenes (Akhtar et al., 1975; Dean-Ross et al., 2001; Evans et al., 1965; Moody et al., 2001), while some fungi species form 9,10-O_2_ANT from ANT (Cajthaml et al., 2002; Krivobok et al., 1998; Ye et al., 2011). The gas-particle partitioning would suggest a higher ratio in soil than in air, since the particulate fraction of the quinone is higher than of the parent PAH (Košetice 2015–2017; Nežiková et al., 2021). Indeed, 9,10-O_2_ANT is degraded faster than ANT (Matscheko et al., 2002).

### NPAHs

Out of the 17 targeted NPAHs and one NOPAH included into the group of NPAHs, 5 were found in soils from Košetice and Mokrá, respectively (Fig. S3c). However, it has to be considered that some low MW NPAHs could have been abundant but not detected due to the low recovery of the low MW NPAH 1-NNAP-D7 (see “Quality control” section and S.1.3). As shown in Fig. S3c, the three detected high MW NPAHs (≥ 4 ring NPAHs) had a detection frequency of > 85%, while the two detected low MW NPAHs had a detection frequency of 26% and 28%. For the results such as the sum of all NPAHs (∑_18_NPAHs), the concentrations < LOQ of substances with a detection frequency < 25% are replaced by 0 ng g^−1^, substances detected more often by LOQ/2. The LOQs are found in Table S7 in the SI. The annual concentrations of ∑_18_NPAHs and individual NPAHs are shown in Fig. 1e, f and S4 (normalized to soil TOC content) as well as Tables S9 (pg g^−1^) and S10 (ng (g TOC)^−1^), respectively.

Similar to the PAHs and OPAHs, lower NPAH levels are found in Košetice (0.31 ± 0.23 ng g^−1^) than in Mokrá (0.54 ± 0.45 ng g^−1^), though not significant (*p* = 0.11, Student’s t-test). It should be considered that we are comparing the average of different sampling years between Košetice and Mokrá. The temporal variation (coefficient of variation) at the individual locations is between 36 and 97%.

When comparing the TOC normalized concentrations, the two sites are different, not significantly but close to significance (*p* = 0.054, Student’s t-test) (see Fig. S5f). The concentration of ∑_18_NPAHs at Košetice-1 is lower than at Košetice-2 (0.26 ± 0.26 ng g^−1^ vs 0.35 ± 0.20 ng g^−1^, not significant: *p* = 0.49, Student’s t-test) but higher when normalizing to the TOC content (13.7 ± 12.9 ng (g TOC)^−1^ vs 6.5 ± 3.7 ng (g TOC)^−1^, not significant: *p* = 0.19, Student’s *t*-test). The highest concentration (0.65 ± 0.62 ng g^−1^) was found at Mokrá-1. However, this is mainly caused by an exceptionally high NPAH concentration in one soil sample, from summer 2006. Without this value, the mean concentration of Σ_18_NPAHs at Mokrá-1 is 0.42 ± 0.13 ng g^−1^, similar (*p* > 0.95, Student’s *t*-test) to the other location, Mokrá-2 (0.42 ± 0.15 ng g^−1^).

We did not find significant correlations (*p* < 0.05) between the concentrations of the individual NPAHs or ∑_18_NPAHs and the TOC content of the studied soils. Similar results were also reported by Cai et al. (2017) in their study. They explained that the lack of correlation between NPAH concentrations and TOC content in soils sampled over a wide spatial scale in China was due to the higher mobility of NPAHs and OPAHs compared to PAHs (see log K_ow_ in Table S3). Our findings and their finding are however in contrast to the study of Sun et al. (2017), which reported significant correlations between the concentrations of NPAHs and the TOC content in soils.

A comparison to other studies is shown in Table S16. The average concentration of the Σ_18_NPAHs in soils from Košetice is similar to concentrations determined in urban soil from Basel, Switzerland, with 0.34 ng g^−1^ (Σ_8_NPAHs, Niederer, 1998) and from Hanoi, Vietnam, with 0.32 ng g^−1^ (Σ_10_NPAHs, Pham et al., 2015). The average concentration of Σ_18_NPAHs at the semi-urban site Mokrá from this study (0.54 ng g^−1^) was slightly higher than the above-mentioned concentrations. Soil from an urban site in Göteborg in Sweden, from the Yangtze River Delta in China and Ejby in Denmark showed similar concentrations with 0.54 ng g^−1^ (Σ_8_NPAHs, Brorström-Lundén et al., 2010), 0.60 ng g^−1^ (Σ_12_NPAHs, Cai et al., 2017) and 0.50 ng g^−1^ (Σ_3_NPAHs, Vikelsøe et al., 2002), respectively. All other sites showed a higher NPAH burden, although the number of examined NPAHs was generally lower. The studies including rural soils from China by Bandowe et al. (2019), from Gardsjön, a rural site in Sweden studied by Brorström-Lundén et al. (2010), and from the site Ejby in Denmark, investigated by Vikelsøe et al. (2002), are the only rural/background site in the literature reporting NPAH soil burden up to now.

#### Comparison to NPAHs’ air concentration

Similar to the OPAHs, we compared the NPAH concentrations at Košetice in soil to the concentrations in air from the study of Nežiková et al. (2021). Only 4 of the 12 NPAHs detected in air were found in soil. This might mainly be limited by LOQs in soil due to low concentrations at the background and semi-urban sites based on the low air concentrations, but it should also be considered that degradation and leaching in soil may have caused dissipation of NPAHs. However, estimates of the soil adsorption coefficient, the biodegradability and the log K_ow_ (USEPA, 2019, Table S3) do not show an exceptionally high or low value to explain the disappearance of any particular NPAH detected in air but not in soil. A major difference between the detected NPAHs in air and soil is the relative abundance of 2 + 3-NFLT being higher in air than in soil and of 1-NPYR being higher in soil compared to the air, which will be explained in more detail in “Composition pattern of NPAHs” section.

#### Composition pattern of NPAHs

**Fig. 3:**
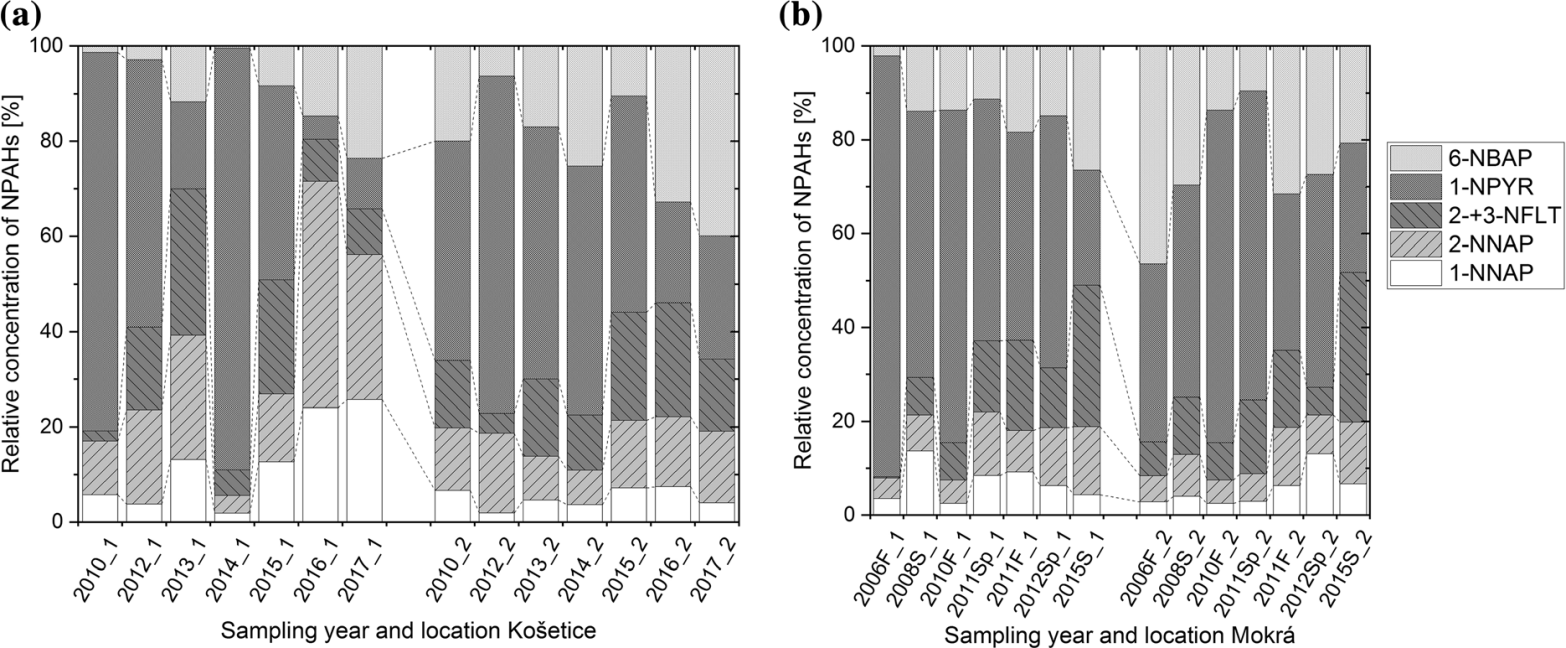
Composition pattern of NPAHs **a** in Košetice **b** in Mokrá (F, fall; Sp, spring; S, summer); Absolute data in Table S9

The NPAHs’ composition patterns are shown in Fig. 3, temporally averaged in Fig. S6. The highest contribution between 50 and 66% is from 1-NPYR, followed by 6-NBAP (4–25%), which is very different from the NPAH pattern in air (≈1% and < 1%, respectively; Tomaz et al., 2016; Lammel et al., 2020; Nežiková et al., 2021) shown in Fig. S7c and probably explained by susceptibility to photolysis in air (see below, “NPAH/PAH ratios” section) in combination with slow degradation in soil (Table S3). As visible in Fig. 3, the contribution of 2- + 3-NFLT is in all samples between around 5–30%. In contrast, the contribution of 2- + 3-NFLT (relative to the sum of all NPAHs detected in both compartments) in air from Košetice measured by Nežiková et al. (2021) is 74% (Fig. S7c). Nežiková and colleagues showed that only 2-NFLT is abundant in air, while 3-NFLT could not be detected. Similarly, 2-NFLT is dominating the NFLT isomers in soil at the background and the semi-urban site. The separation of the two isomers was inadequate to quantify 2-NFLT and 3-NFLT separately but good enough to qualitatively report that 3-NFLT was either not detected or only detected as a small shoulder up to 5% of the peak area of the peak of 2-NFLT. This finding is in line with previous knowledge from air: 2-NFLT dominates NFLT isomers in ambient urban and even more so in rural and remote atmospheric environments (2-NFLT/(2-NFLT + 3-NFLT) > 0.96), while 3-NFLT dominates NFLT isomers in exhaust of diesel engines only (Bamford et al., 2003; Schantz et al., 2005; Zimmermann et al., 2013; besides other).

In air samples, the ratio of the concentration of 2-NFLT to the concentration of 1-NPYR is often used as a diagnostic ratio for the relative importance of photochemical formation since 1-NPYR is known to be emitted primarily, while 2-NFLT is secondarily formed (Bandowe & Meusel, 2017). The ratio of 2- + 3-NFLT/1-NPYR (assuming 2- + 3-NFLT is approximately similar to 2-NFLT) in soil in this study ranging between 0.35 and 0.76 across the four locations is much lower than in air (23 at Košetice; Nežiková et al., 2021). Parameters that could influence this ratio in soil are deposition velocity, leachability and biodegradation or formation in soil. The deposition velocity hardly adds to the selectivity because of similarly high particulate mass fractions at Košetice (Nežiková et al., 2021) as well as at urban sites, also in the region (Lammel et al., 2020; Tomaz et al., 2016). The leachability depends on sorption and water solubility. As shown in Table S3, the calculated soil adsorption coefficient for both compounds is the same, while log K_ow_ of 1-NPYR is higher than of 2-NFLT (5.06 vs. 2.55 according to the KOWWIN model; USEPA, 2019). 2-NFLT is degraded faster than 1-NPYR (3.42 or days-weeks vs. 2.68 or weeks-months, respectively, according to BioWin 4; USEPA, 2019), leading to lower ratios in soil compared to the atmosphere. The indication of relative importance of primary emissions by this ratio from air is obviously not preserved in soil. However, the ratio is higher at the background site Košetice compared to the semi-urban site Mokrá (0.61 ± 0.58 vs. 0.36 ± 0.38).

#### NPAH/PAH ratios

The ∑_18_NPAHs are found 2 to almost 3 orders of magnitude lower than the ∑_16_PAHs. The temporal average ratios of NPAHs and the corresponding parent PAHs are illustrated in Fig. S10 in the SI. It can be hypothesized that the high concentration ratio of the sum of NPAHs to the sum of PAHs at the background site Košetice-1 is due to deposited long-range transported aerosols forming NPAHs in the atmosphere by reactions of parent PAHs with atmospheric oxidants. This is in line with the finding of a similar 2- + 3-NFLT/FLT concentration ratio between the soil at Košetice-1 and the air samples from Košetice with the secondarily formed 2-NFLT being dominant in both compartments (Nežiková et al., 2021) (Fig. S10b). The higher 1-NPYR/PYR concentration ratio in soil compared to air may be due to the higher persistence in soil compared to the corresponding PAH (lower biodegradability, by BIOWIN, USEPA, 2019; see Table S3) and shielding from photolysis, while the ratios 1-NNAP/NAP, 2-NNAP/NAP and, as already said, 2- + 3-NFLT/FLT in soil samples seem to be preserved from air. 3-NFLT and 1-NPYR are susceptible to photolysis in air due to a peri-H atom (Fan et al., 1995), which also applies to 6-NBAP (2 peri-H’s), abundant in the soil samples, too (see above, “Composition pattern of NPAHs” section), while there are no peri-H atoms in 2-NNAP and 2-NFLT. Formation of NPAHs in soil is not indicated by the findings; degradability and sorption may vary considerably across NPAHs.

### Temporal variations of PACs in soil

The results of the annual PAH, OPAH and NPAH concentrations, disaggregated for numbers of rings, are shown in Fig. 1 and Fig. S4 (normalized to TOC content). The OPAH concentrations at the two locations in Mokrá follow a similar direction of the variation (except for fall 2010 and spring 2011) meaning, e.g. a decrease between fall 2006 to summer 2008 following by an increase between 2008 and fall 2010. However, this only shows the similar behaviour at both sites in Mokrá and not a trend between the years since at Mokrá we compare different seasons. Except for the samples from 2006, the NPAH concentrations at Mokrá-1 and Mokrá-2 also follow a similar direction of temporal variation but not the same as for the OPAHs. The NPAH concentration at Mokrá is smaller in summer 2015 compared to spring 2012 at both locations but for the OPAHs, the opposite is true for Mokrá-1 and Mokrá-2. The finding suggests that the soils from both locations at Mokrá receive the same varying primary pollution and that the transport processes influence the concentrations of the target compounds in these soils to a similar extent. The same is true for the NPAHs at the different locations in Košetice. Only in between 2016 and 2017, the behaviour at both locations is different meaning the NPAH concentration at Košetice-1 in 2017 is smaller than in 2016, while at Košetice-2 it is higher. In contrast, the OPAHs in Košetice do not follow a similar direction of the annual variation, except 2015–2017.

The concentrations of the Σ_18_NPAHs at all examined locations decrease from older to contemporary samples (Fig. 1), significant for one out of four locations, i.e. Mokrá-2 (*p* > 0.95, Neumann test, Hecht, 2020). The compounds most contributing to the decrease are 1-NPYR and the 2-ring NPAHs. In contrast, the concentrations of 2- + 3-NFLT and 6-NBAP are mainly increasing.

An increasing trend (not significant; p = 0.91, Neumann trend test) is seen for Σ_11+2_OPAHs and O-heterocycles at Mokrá-1 (Fig. 1). No such trend is seen for the Košetice and Mokrá-2 locations. The increasing OPAH concentration at Mokrá-1 is mainly driven by the 4–5-ring OPAHs. These are also slightly increasing at Mokrá-2 and Košetice-1. In contrast, it seems that the 3-rings DBF and 9-OFLN decrease from older to contemporary samples at all locations (significant for DBF at Košetice-2, *p* > 0.95; not significant at Košetice-1; p = 0.94, Neumann trend test). We hypothesize that the decrease might be related to increasing winter temperatures. Similar results were found for the PAHs. The 3-ring PAHs FLN and PHE are decreasing at all locations except for Mokrá-2 (significant for FLN at Košetice-1 and Košetice-2 and for PHE at Košetice-1, *p* > 0.95, Neumann trend test), while almost all other PAHs either stay the same or are increasing. In total, the concentration of Σ_27_PAHs stayed constant at both locations in Košetice (Fig. 1a) in the studied period. At Mokrá, the PAH concentrations increase (not significant, Neumann trend test), although at Mokrá-2 this is strongly influenced by high levels in 2015 (Fig. 1b). Similar results were found for the concentration of Σ_16_PAHs except for a slight, not significant decreasing trend at Košetice-1. Comparable results were determined when studying even longer time series of the same locations (Chapter S3 in the SI).

Nežiková et al. (2021) found a decreasing trend of several NPAHs and OPAHs in air in Košetice between 2015 and 2017 attributable to ongoing emission reductions of PAHs that are also effective for NPAHs and OPAHs. This could be one reason for the decreasing NPAH concentrations. Unfortunately, long-term measurements of PAHs, NPAHs and OPAHs in air at Mokrá to compare with are not available in the literature. Long-term studies for PACs in soil are so far only available for PAHs. Gubler et al. (2015) found similar results in Swiss soil samples between 1985 and 2013 as we found in the Czech soil samples, namely a decreasing trend for low MW PAHs and no or slightly increasing trend for the high MW PAHs. A decreasing trend of PAHs was examined for the period 1990–2009 in Scottish soil by Cui et al. (2020). Given the limited number of years sampled and the complex dependencies of the deposition, leaching, degradation and formation mass fluxes on climate parameters, the observed variabilities of the PAH derivatives in soil are not conclusive. Longer time series and deeper process understanding are needed.

## Summary and conclusion

The concentrations of the PAH derivatives in soil at the central European background site Košetice are among the lowest ever reported. The average concentration in the studied period of the Σ_18_NPAHs and the Σ_11+2_OPAHs and O-heterocycles was 0.31 ± 0.23 ng g^−1^ and 4.07 ± 3.08 ng g^−1^, respectively. At the semi-urban site Mokrá, the concentrations were slightly higher (0.54 ± 0.45 ng g^−1^ and 5.91 ± 2.30 ng g^−1^, respectively). Our results show that several NPAHs and OPAHs are abundant in soil at the background site. 1-NPYR and 6-NBAP, which are identified as highly toxic, were the most abundant NPAHs in soil. The findings suggest that 1-NPYR and 6-NBAP are more stable in soil than in air. Through eventual revolatilization, soil may turn into a secondary source of this and other NPAHs and OPAHs. The contributions of OPAHs were more equally distributed, showing the highest contributions from 9-OFLN, 11-OBaFLN and 11-OBbFLN. We found a correlation of the soil organic carbon content with the high MW OPAHs and PAHs, but not with the low MW OPAHs and PAHs, nor with the NPAHs. The temporal variation of the concentration of PACs in soil at Košetice and Mokrá significantly differed between substances. It could be noticed that the concentrations of dibenzofuran, 9-fluorenone and some 3-ring PAHs decreased, while it stayed constant or increased for higher MW PACs. In order to make more reliable statements about the temporal variation of the soil concentrations, longer time series and deeper understanding of the PAC chemodynamics in soil are needed.

## Supplementary Information

Below is the link to the electronic supplementary material.

Supplementary file1 (DOCX 5071 kb)

## Data Availability

All data are supplied in the SI or can be requested from the authors.
