# Supplementary material for: Nitro- and oxy-PAHs in grassland soils from decade-long sampling in central Europe
Source: Environ Geochem Health. 2021 Aug 20;44(8):2743–65. doi: 10.1007/s10653-021-01066-y (PMC9213387; doi:10.1007/s10653-021-01066-y)
Supplement: Supplementary file 1 — Supplementary file1 (DOCX 5071 kb) [file 10653_2021_1066_MOESM1_ESM.docx]

**Supplementary Information**

**Nitro- and oxy-PAHs in grassland soils from decade-long sampling in central Europe**

M. Wietzoreck^a^, B.A.M. Bandowe^a^, J. Hofman^b^, J. Martiník^b^, B. Nežiková^b^, P. Kukučka^b^, P. Přibylová ^b^, G. Lammel^a,b^

^a^Max Planck Institute for Chemistry, Multiphase Chemistry Dept., Mainz, Germany

^b^Masaryk University, Research Centre for Toxic Compounds in the Environment, Brno, Czech Republic

**Contents**

[S1 Quality control 5](#_Toc76462290)

[S1.1 Method validation 5](#_Toc76462291)

[S1.2 Limit of quantification and blank correction 5](#_Toc76462292)

[S1.3 Recoveries 6](#_Toc76462293)

[S1.4 Repeatability 7](#_Toc76462294)

[S2 Comparison of remeasured to original PAH results 8](#_Toc76462295)

[S3 PAHs multiannual variation 9](#_Toc76462296)

[Tables and Figures 12](#_Toc76462297)

[References 55](#_Toc76462298)

**List of Tables**

**Table S1** Information about soil sampling sites and dates………………………………………...12

**Table S2** Basic soil parameters of the analysed soil samples, n.d. = not determined……………14

**Table S3** Physico-chemical properties & degradation rates of a) PAHs, b) OPAHs and O-heterocycles, c) NPAHs; k_biodeg.1_: > 0.50: Likely to biodegrade rapidly, < 0.50: Not likely to biodegrade rapidly, k_biodeg. 2_: >4.75 – 5: Hours, >4.25 - 4.75: Hours – days, >3.75 - 4.25: Days, >3.25 - 3.75: Days – weeks, >2.75 - 3.25: Weeks, >2.25 - 2.75: Weeks – months, >1.75 - 2.25: Months, <1.75: Longer “recalcitrant”; MW = Molecular weight, n.d. = not determined………….16

**Table S4** SIM masses and retention times of PAHs in GC-MS analysis………………………….19

**Table S5** MRM masses and retention times of NOPAHs in GC-MS……………………………...21

**Table S6** Recoveries of target compounds from duplicate measurements………………………..21

**Table S7** Limits of quantification (LOQs) of a) PAHs b) OPAHs and O-heterocycles and c) NPAHs in ng g^-1^ (mLOQ: method LOQ; iLOQ: instrumental LOQ; STD: Standard deviation)….22

**Table S8** Repeatability (CoVs) of individual PACs, a) PAHs, b) OPAHs and O-heterocycles and c) NPAHs, of the 11 samples measured in duplicate or triplicate (STD: Standard deviation)……..25

**Table S9** Concentration of a) Σ_11_OPAHs, Σ_2_O-heterocycles, Σ_16_PAHs, Σ_27_PAHs in ng g^-1^ and b) ∑_18_NPAHs and individual NPAHs in pg g^-1^ (F: Fall; Sp: Spring; S: Summer). Values <LOQ were replaced by LOQ/2 if the detection frequency was >25 % (**Fig. S3**), else replaced by 0 ng g^-1^……31

**Table S10** Same as Table S9 but normalized for soil TOC content in ng (g TOC)^-1^………………33

**Table S11** Concentrations of PAHs in soil samples from a) Košetice-1, b) Košetice-2, c) Mokrá-1 and d) Mokrá-2 in ng g^-1^ (F:Fall; Sp:Spring; S:Summer; STD: Standard deviation)………………35

**Table S12** PAH concentration in surface soil (sampling depth 10 cm)…………………………...39

**Table S13** Concentrations of OPAHs and O-heterocycles in soil samples from a) Košetice-1, b) Košetice-2, c) Mokrá-1 and d) Mokrá-2 in ng g^-1^ (F: Fall; Sp: Spring; S:Summer; STD: Standard deviation). Values <LOQ were replaced by LOQ/2 if the detection frequency was >25 % (Fig. S3), else replaced by 0 ng g^-1^…………………………………………………………………………40

**Table S14** Temperature and precipitation compared to average in Košetice and Mokrá (Czech Hydrometeorological Institute 2005-2017)………………………………………………………48

**Table S15** OPAH/PAH ratios in soil at different locations; n.d.: not determined…………………51

**Table S16** NPAH concentration in surface soil (ng g^-1^) at different locations; n.d. = not determined; <x = smaller than not reported limit……………………………………………………………….52

**Table S17** Sum of 16 EPA-prioritized PAHs, Σ16PAH, in soil at Košetice and Mokrá in ng g^-1^ (STD: Standard deviation)………………………………………………………………………54

**List of Figures**

**Fig. S1** Sampling locations Košetice-1, Košetice-2, Mokrá-1 and Mokrá-2 (Satellite image taken from CENIA, Czech Environmental Information Agency, Software used: ArcGIS 9.6, ESRI, Redlands, USA) ……………………………………………………………………………13

**Fig. S2** ∑_16_PAHs concentration from measurement done temporarily close to the sampling date, c_original_, compared to ∑_16_PAHs concentration from re-measurement, c_archived_ _sample_, of the archived soil samples. Linear fit with all values (red fit line with equation c_archived sample_ = 0.35 c_original_ + 94.25; R = 0.58, p<0.05) and linear fit without two outlier values from Mokrá-1 and Mokrá-2 (marked in red) (blue fit line with equation c_archived sample_ = 0.93 c_original_ + 33.77; R = 0.86, p<0.05 )…………...27

**Fig. S3** Detection frequency of a) PAHs, b) OPAHs and O-heterocycles and c) NPAHs using the sum of the average of blanks and 1 × standard deviation of blanks as LOQ……………………….28

**Fig. S4** Concentration normalized for TOC content in ng (g TOC)^-1^ of A & B: Σ_27_PAHs split into 2-ring (white), 3-ring (light grey), 4-ring (grey) and 5-7-ring PAHs (dark grey), A: at Košetice-1 (plain) and Košetice-2 (dashed); B: at Mokrá-1 (plain) and Mokrá-2 (dashed); C & D: Σ_11+2_OPAHs and O-heterocycles split into 2-ring (white), 3-ring (light grey), 4-ring (grey) and 5-ring OPAHs (dark grey), C: at Košetice-1 (plain) and Košetice-2 (dashed); D: at Mokrá-1 (plain) and Mokrá-2 (dashed); E & F: Σ_18_NPAHs split into 2-ring NPAHs (white), 3-ring NPAHs (light grey), 4-ring NPAHs (grey) and 5-ring NPAHs (dark grey), E: at Košetice-1 (plain) and Košetice-2 (dashed); F: at Mokrá-1 (plain) and Mokrá-2 (dashed); (F: Fall; Sp: Spring; S: Summer)……………………..29

**Fig. S5** Box-and-whisker-plot of location average concentrations of Σ27PAHs (a and d), Σ_11_OPAHs (b and e) and Σ_18_NPAHs (c and f) in ng g^-1^ (a-c) and normalized for TOC content in ng (g TOC)^-1^ (d-f) (empty square: Mean value; Filled squares: Measurement points; Filled box with extra borders: Interquartile range (IQR) bound by the 75th and 25th percentile and range of 1.5 IQR; Horizontal line: Median)……………………………………………………………………30

**Fig. S6** Location average of relative concentrations of a) 16 PAHs, b) 11 OPAHs and 2 O-heterocycles and c) 18 NPAHs in soil from Košetice and Mokrá (average of all examined years) ……………………………………………………………………………………………………44

**Fig. S7** Location average of relative contribution of a) 16 PAHs, b) 11 OPAHs and 2 O-heterocycles and c) 18 NPAHs in air at Košetice (taken from Nežiková et al. 2021) and in soil. The relative contributions in soil and air from Košetice show the average of the years 2015-2017, the mean of all examined years is shown for the locations in Mokrá………………………………….46

**Fig. S8** Relative concentrations of different ring size PAHs to the ∑_27_PAHs a) in Košetice and b) in Mokrá, split into 2-3-ring PAHs (white), 4 ring PAHs (light grey) and 5-7-ring PAHs (dark grey) ……………………………………………………………………………………………………47

**Fig. S9** Average ratio of OPAHs and corresponding parent PAHs in soil from Košetice and Mokrá a) of all examined years and b) of 2015-2017 at Košetice-1 and -2, of air data from 2015-2017 at Košetice (data from Nežiková et al. 2021) and for all examined years from Mokrá soil. Since BBN was not measured by Nežiková et al., the ratio 11-OBbFLN/BBN is not available for air in Košetice. “*” shows the significance with p<0.05 (Student’s t-test). In b), only the significance between Košetice-1 air and soil was tested but difficult to achieve with only 3 soil samples between 2015-2017. Error bars show the standard deviation of the ratio from different years. Lower limit value for the ratio 9,10-O_2_ANT/ANT since detection frequency of 9,10-O_2_ANT was <25 % (23 %). For calculation: Values <LOQ were replaced by LOQ/2 if the detection frequency was >25 % (**Fig. S3**), else replaced by 0 ng g^-1^. “*” shows the significance with p<0.05 (Student’s t-test) in diagram a)………………………………………………………………………………………...49

**Fig. S10** Ratio of NPAHs and corresponding parent-PAHs in soil from Košetice and Mokrá a) of all examined years and b) of 2015-2017 at Košetice-1 and Košetice-2, of the air data from 2015-2017 at Košetice (data from Nežiková et al. 2021) and for all examined years from Mokrá soil. Since 6-NBAP was not measured by Nežiková et al., the ratio 6-NBAP/BAP is not available for air in Košetice. Ratios of 1-NNAP/NAP and 2-NNAP/NAP are upper limits, since NNAP values <LOQ were replaced by LOQ/2 (detection frequency >25% i.e., ≈30 %). “*” shows the significance with p<0.05 (Student’s t-test) in diagram a)………………………………………………………50

# S1 Quality control

## S1.1 Method validation

The method was validated by a spike and recovery experiment. We performed the spiking experiments with top 10 cm surface soil within the A horizon after removing the vegetation layer collected at a grassland site in Mainz, Germany. The dried and sieved soil was spiked with 10 µL of a target analyte mix (1.5 ng µL^-1^) directly onto 5 g portions of the soil (n =4) resulting in 15 ng of each target compound in the soil. The PACs were then determined in the spiked (n =2) and unspiked (n =2) soils as described below.

The extraction was done by Soxhlet (5 g of soil in extraction thimble) with 200 mL DCM/acetone (2:1, v:v) for 40 min (Soxhlet: Büchi B-811, Flawil, Switzerland). The purification was done by solid phase extraction using SiOH cartridges (6 mL, 2000 mg, Chromabond) eluting the target substances by ethyl acetate and dichloromethane. In addition, we re-extracted the soil samples with 200 mL DCM by Soxhlet extraction in order to check whether a re-extraction reveals a significant additional amount of substances making an improvement of the extraction efficiency necessary. The amount of target compounds from the second extraction of the soil was insignificant indicating that a the first extraction with DCM/acetone (2:1 v/v) was sufficient to recover the spiked PACs.

In order to calculate the recoveries, we subtracted the average amount of the duplicate measurement of the spiked samples by the average amount of the duplicate measurement of the unspiked sample and divided it by the expected amount of spiked substance of 15 ng. **Table S6** shows the recoveries of the target compounds. The recoveries were 132 ± 41 (61-171) % and 108 ± 17 (81-152) % for OPAHs and NPAHs, respectively. However, it has to be considered that these results are the upper limits of the recoveries since in real soil samples the substances can undergo stronger binding to the soil matrix than in the 30 min after spiking the soil in the recovery experiment.

## S1.2 Limit of quantification and blank correction

For the evaluation of the results, we used two different limits of quantification (LOQs) i.e., first, the instrumental LOQ, iLOQ, based on the GC-MS analysis, and, secondly, the method LOQ, mLOQ. For NOPAHs, the iLOQ is calculated on the signal to noise ratio (S/N) for a specific compound and specific sample as the concentration which corresponds to S/N = 10:1. For PAHs, the iLOQ is calculated by extrapolation of S/N = 10:1 to the corresponding concentration, based on calibration standards. The LOQs can be found in **Table S7**. We measured five method blanks (same sample preparation but without any soil) and calculated the mLOQ by calculating the mean concentration of a compound from the blanks µ_mb_ plus the standard deviation σ_mb_ of the blanks:

mLOQ = µ_mb_ + σ_mb_

The average amounts of the target compounds in the method blanks, µ_mb_, were also used for blank correction. The final concentration C_i_ was calculated as follows:

1. If a value was higher than the LOQ, the raw concentration of a compound c_i_ was subtracted by the mean of the method blanks µ_mb_

C_i_ = c_i_ - µ_mb_

1. If a value was lower than the LOQ (using the maximum of iLOQ and mLOQ), we used the half of the value of LOQ subtracted by the mean of the method blanks µ_mb_:

C_i_ = (max(iLOQ, mLOQ) - µ_mb_)/2

In case that the total detection frequency of a compound in all samples was lower than 25 %, a concentration of 0 was used for further calculations such as ∑_18_ NPAHs

## S1.3 Recoveries

The average recoveries (compared to the target amount) of 1-NNAP-D7, 2-NFLN-D9, 9-NANT-D9, 3-NFLT-D9, 1-NPYR-D9, 6-NCHR-D11 and 6-NBAP-D11 in the samples and blanks were 15 ± 10 %, 78 ± 25 %, 55 ± 34 %, 41 ± 23 %, 32 ± 24 %, 31 ± 19 % and 43 ± 47 %, respectively. Due to the high variability of the recoveries (CoV of 32-73 %) and the relatively low recoveries, the NPAH data was recovery corrected. The amounts of NPAHs detected in the samples were recovery corrected using the recoveries of their respective surrogate standards. Each compound was corrected with the corresponding deuterated NPAH. The amounts of 1-NNAP and 2-NNAP were corrected by the recovery of 1-NNAP-D7; 2-NFLT and 3-NFLT with 3-NFLT-D9; 1-NPYR with 1-NPYR-D9 and 6-NBAP with 6-NBAP-D11. For all other investigated NPAHs, the recovery correction was not needed since none of them was measured in concentrations higher than the LOQ.

The average recoveries (compared to the target amount) of NAP-D8, PHE-D10 and PER-D12 in the samples and blanks were 37 ± 17 %, 75 ± 12 % and 102 ± 16 %, respectively. The recovery correction was applied only for the samples, namely Kos_2010Sp_1b, Kos_2015_1b and Mok_2012_2a, with a recovery of less than 10 % for NAP-D8 due to evaporation of the extract to almost dryness. NAP and BPH were corrected by NAP-D8; ACY, ACE, FLN by the average of the recoveries of NAP-D8 and PHE-D10 and PHE by PHE-D10. The concentrations of the compounds were corrected by the average recovery of their surrogates in all samples.

The average recoveries (compared to the target amount) of 9-OFLN-D8 and 9,10-O_2_ANT-D9 in the samples and blanks were 101 % ± 34 % and 107 % ± 33 %.

The results of the PAHs and OPAHs were not recovery corrected since their recovery was acceptable and the variability of the recovery of the deuterated compounds was low (CoV of 15-33 %). The variability of the relative recoveries was smaller than the variability of the compound concentrations of the repeated measurements (shown in **Table S8**).

## S1.4 Repeatability

A number (n = 11) of the soil samples were measured in duplicate or triplicate to determine the reproducibility of the method. The intra-sample coefficient of variation (CoV) for the 11 samples averaged 15±12 % (range: 2-40 %) for the ∑_16_PAHs. The CoVs of the individual PAHs can be seen in **Table S8a**.

Typical CoVs for the reproducibility of PAHs in soil (from round robin tests) are 15-20 % for concentrations of 40-100 mg kg^-1^. For each individual PAH, CoV is 30-40 % for concentrations of 2-15 mg/kg and 40-60 % for concentrations <2 mg kg^-1^ (UBA 2003). Since their evaluation shows that the CoV increases with decreasing concentrations, and the concentrations of the analysed soils are around three orders of magnitude lower than the concentrations in the study, the CoV for the reproducibility for PAHs could easily be 20-30 % for the same method and 20-40 % or more for a different sample preparation method. In comparison of the two measurements, only 6 out of 30 (i.e., 20 %) of our analysed samples show CoV >40 % and only 4 samples (13 %) show CoV >50 %.

The intra-sample CoV of the Σ_11_OPAHs + 2 O-heterocycles is 22 ± 17 % (5-60 %). As shown in **Table S8b**, the CoV of the individual OPAHs ranged from 21 % by 5,12-O_2_NAC to 48 % by 9-OFLN. The intra-sample CoV of the Σ_18_NPAHs (**Table S8c)** (in fact quantified only 6) is 27 ± 25 % (0.7-71 %). The CoV is not very different for the individual NPAHs. It is the lowest for 2-NFLT with 26 % followed by 6-NBAP and 2-NNAP with 28 and 31 %, respectively. 1-NNAP, 3-NFLT and 1-NPYR showed only a slightly higher CoV with 38 %, 34 % and 36 %, respectively.

# S2 Comparison of remeasured to original PAH results

The concentrations of 16 US-EPA PAHs measured in this study (“archived samples”) are similar to the previous measurements (“original”) of the same samples (**Fig. S2**), except for two soil samples from Mokrá (Mokrá_2010F_1 and Mokrá_2006F_2), which are outliers (P<0.01, Grubbs’ test). The PAH concentrations of the re-measurement of these two samples are shifted to a significantly lower concentration. The relative differences between both measurements ((c_archived sample_ - c_original_) (c_archived sample_ + c_original_)^-1^) are -94 ± 54 % and -91 ± 57 % for Mokrá_2010F_1 and Mokrá_2006F_2, respectively. Possible reasons for the significant differences in the concentrations of these two samples are soil heterogeneity at a small spatial scale (< 10 m) and/or reduced extractability (MacLeod & Semple 2003).

The correlation coefficient between the concentration of the original and the archived samples is 0.47 (p = 0.06), while it is 0.90 without the two outliers (p <0.01). The site-specific correlation coefficients are 0.83 (p<0.05) for Mokrá-1 and 0.89 (p<0.05) for Mokrá-2 without the two outliers (0.07 and 0.56 (p = 0.87 and p = 0.15) for all samples of Mokrá-1 and Mokrá-2, respectively). The correlation coefficients for the two PAH measurements of soil from Košetice are 0.12 and 0.41 (p = 0.80 and p = 0.36) for locations 1 and 2, respectively. Without sample Košetice_2015_2, for which a large difference between both measurements is found, the correlation coefficient is 0.82 (p<0.05). However, this is not an outlier (Grubbs test). The small correlation coefficient for Košetice-1 might be explained by a higher uncertainty of the concentrations close to the limit of quantification.

The difference between the concentration of the archived and the original measurement for each individual sample compared to the average of both measurements shows that the results from the archived samples is on average 7 ± 21 % higher than the original data. However, this is slightly biased by the two samples from Mokrá (Mokrá_2010F_1 and Mokrá_2006F_2) with comparably high PAH concentrations in the original measurement. Excluding these two samples, the re-measured concentrations are 11 ± 17 % higher than the original data. Although this is lower than the variability of the concentration of the Σ_16_PAHs from the repeatability experiments of 11 soil samples, which is 14.7 %, we try to reveal the cause for this deviation. Possible reasons for the differences are (a) the extractability of PAHs in soil has increased during sample storage or (b) sample contamination during storage or (c) difference in sample preparation or (d) soil heterogeneity. The soil samples were air-dried before the first analysis and stored under controlled conditions. Hence, it is unlikely that air contamination during sample storage caused the observed differences. Explanation (a) can be rejected, because there was no trend between concentration differences and storage time. Furthermore, MacLeod et al. (2003) found that the non-extractable fraction tends to increase with storing time, rather than decrease. Reason (b) is very unlikely since Cousins et al. (1997) found that contaminations during air-drying mainly affect the lower MW PAHs. In this work, the difference between the original PAH concentration and the PAH concentration of the remeasured archived samples were mainly due to the higher MW PAHs. Therefore, we conclude that this could not be due to air contamination during storage. We suggest that (c), the difference in sample preparation might have caused the observed differences. For the higher recovery of the OPAHs, a slightly more polar extraction solvent mixture was used compared to the original PAH measurement. Hollender et al. (2002) and Lau et al. (2010) found that slightly polar solvents can increase the extractable amount of PAHs in soil with a low degree of pollution, since it can break up soil aggregates. In addition, (d) soil heterogeneity, might play an important role for the observed differences, too. However, this relatively small difference between both measurements should not be overstated, since the uncertainties in determining representative pollutant concentrations in soil samples are generally higher than in other environmental matrices, such as water or air. This could also be seen from the repeatability results of our samples, which are shown as quality control data (**Table S8**). The variation between different measurements of the same sample can be significant for soil samples (UBA 2003). In summary, the PAHs in the archived soil samples were not prone to degradation or any other strong modification. The concentrations of the re-measured compounds are comparable to the original results determined shortly after sampling.

# S3 PAHs multiannual variation

The annual PAH concentrations of the samples from this study are shown in **Table S11**. In order to reveal time-related trends, we looked at even longer time series. An overview of the PAH concentrations in soil from Košetice and Mokrá from the very same locations from different periods in the past can be found in **Table S17**. Compared to the data from 1996 to 2007 from the same locations in Košetice reported by Holoubek et al. (2007), the concentration of the ∑_16_PAHs in the more recently sampled soils (2010-2017) from Košetice-1 is significantly (p<0.0001, Student’s t-test) lower, by 63 %. The levels at Košetice-2 are 12% lower in the more recent period (2009-2017) compared to 1996-2007. The trend between 1996 and 2017 is not significant at Košetice-2 according to the trend test from Neumann (Hecht 2020). In contrast, there is a significant decreasing trend for the Σ_16_PAHs at Košetice-1 (p<0.01, trend test by Neumann), which was already observed 1996-2007 at several locations, including Košetice-1 (Holoubek et al. 2007). A probable explanation for the difference can be given when looking at the sampling locations in detail. Košetice-1 is at the EMEP station on grassland with no influence other than PAHs from the air by wet and dry deposition. Since the concentration of PAHs in air has decreased during the study period (2010-17) at the regional background site Košetice, weakly but significantly (Kalina et al. 2017; Lhotka et al. 2019; Degrendele et al. 2020), the concentrations in soil might also decrease with a time lag. A decreasing trend for both air and soil had been found at the site for the years 1996-2005 (Holoubek et al. 2007), a faster decrease than later, though. Furthermore, a decrease was also examined for other matrices studied at the site i.e., water, rainwater, and mosses (Prokeš et al. 2019). In contrast, Košetice-2 (location 8 in Holoubek et al. 2007) is on grassland, partly surrounded by trees at the confluence of two brooks resulting in a more diverse impact on the PAH levels. There is first the higher TOC content in this soil and second the possible influence of PAHs from the river water to the examined soil samples in addition to the PAHs from the air. Different TOC content in soil can affect the trend of the pollutant concentration, since it could affect leachability, revolatilisation as well as biodegradability due to stronger interaction between the pollutant and the soil matrix (Wilcke, 2000).

At Mokrá-1, the concentrations of ∑_16_PAHs in the period 1998-2005 are significantly different to 2006-2015. The more recent PAH concentration is 66 % higher than the old one. The trend test by Neumann does not reveal a significant trend, because of an exceptionally high value in April 2001. Excluding this value as an outlier (p<0.01, Grubbs test), a significant increasing trend is found (p<0.05, Neumann trend test). To determine the exact reason, decade-long monitoring of air, deposition and soil samples would be needed. We hypothesize that the trend is caused by a slight change of the sampling site by around 80-100 m in 2007. Even minor changes in the sampling location can have a great impact on the results. This hypothesis is supported in our case by a significant difference in PAH concentrations before and after changing the location (p<0.06, Student’s t-test). Looking at the specific locations individually, there is a significant (p<0.05, Neumann test) decreasing trend for the period 1998-2006 and no trend for the period 2007-2015.

There is no significant trend regarding the concentration of the ∑_16_PAHs at Mokrá-2. The ∑_16_PAHs of this study, 2006-2015, is 6 % higher than the data found for 1998-2005. However, this difference is not significant, but within the variability (CoV = 65 %). Accordingly, the slight change of the sampling position in 2009 did not result in a significant change of the PAH concentration (p<0.22, Student’s t-test), but reflects the variability.

# Tables and Figures

**Table S1** Information about soil sampling sites and dates

| Site | Lo-ca-tion # | Sampling date | Season | Sample abbreviation | Detail location | Latitude (WGS84) | Longitude (WGS84) | Altitude [m] |
| --- | --- | --- | --- | --- | --- | --- | --- | --- |
| Košetice | 1 | 31.08.2010 | Summer | Kos_2010_1^b^ | EMEP station, obser-vatory | 49.57345 | 15.08041 | 534 |
|  |  | 04.09.2012 | Summer | Kos_2012_1 |  |  |  |  |
|  |  | 28.08.2013 | Summer | Kos_2013_1 |  |  |  |  |
|  |  | 25.08.2014 | Summer | Kos_2014_1 |  |  |  |  |
|  |  | 07.09.2015 | Summer | Kos_2015_1^b^ |  |  |  |  |
|  |  | 22.08.2016 | Summer | Kos_2016_1 |  |  |  |  |
|  |  | 22.08.2017 | Summer | Kos_2017_1^b^ |  |  |  |  |
|  | 2 | 31.08.2010 | Summer | Kos_2010_2^b^ | Con-fluence - meadow | 49.571724 | 15.091728 | 489 |
|  |  | 04.09.2012 | Summer | Kos_2012_2 |  |  |  |  |
|  |  | 28.08.2013 | Summer | Kos_2013_2 |  |  |  |  |
|  |  | 25.08.2014 | Summer | Kos_2014_2 |  |  |  |  |
|  |  | 08.09.2015 | Summer | Kos_2015_2 |  |  |  |  |
|  |  | 23.08.2016 | Summer | Kos_2016_2 |  |  |  |  |
|  |  | 22.08.2017 | Summer | Kos_2017_2^b^ |  |  |  |  |
| Mokrá | 1 | 15.11.2006 | Fall | Mok_2006F_1 | Hostěnice Čihálky | 49.234939 | 16.77169 | 427 |
|  |  | 12.09.2008 | Summer/ Fall | Mok_2008S_1 | Hostěnice Čihálky 2 | 49.235283 | 16.770733 | 427 |
|  |  | 21.04.2010 | Spring | Mok_2010Sp_1 |  |  |  |  |
|  |  | 01.10.2010 | Fall | Mok_2010F_1 |  |  |  |  |
|  |  | 21.04.2011 | Spring | Mok_2011Sp_1 |  |  |  |  |
|  |  | 01.10.2011 | Fall | Mok_2011F_1^a^ |  |  |  |  |
|  |  | 25.04.2012 | Spring | Mok_2012Sp_1^b^ |  |  |  |  |
|  |  | 11.06.2015 | Summer | Mok_2015S_1^b^ |  |  |  |  |
|  | 2 | 15.11.2006 | Fall | Mok_2006F_2^b^ | Velká Baba | 49.211188 | 16.784422 | 375 |
|  |  | 12.09.2008 | Summer/ Fall | Mok_2008S_2^b^ |  |  |  |  |
|  |  | 21.04.2010 | Spring | Mok_2010Sp_2 | Velká Baba 2 | 49.211117 | 16.784400 | 375 |
|  |  | 01.10.2010 | Fall | Mok_2010F_2 |  |  |  |  |
|  |  | 21.04.2011 | Spring | Mok_2011Sp_2 |  |  |  |  |
|  |  | 01.10.2011 | Fall | Mok_2011F_2 |  |  |  |  |
|  |  | 25.04.2012 | Spring | Mok_2012Sp_2 |  |  |  |  |
|  |  | 11.06.2015 | Summer | Mok_2015S_2^a^ |  |  |  |  |

^a^measured in duplicate ^b^measured in triplicate


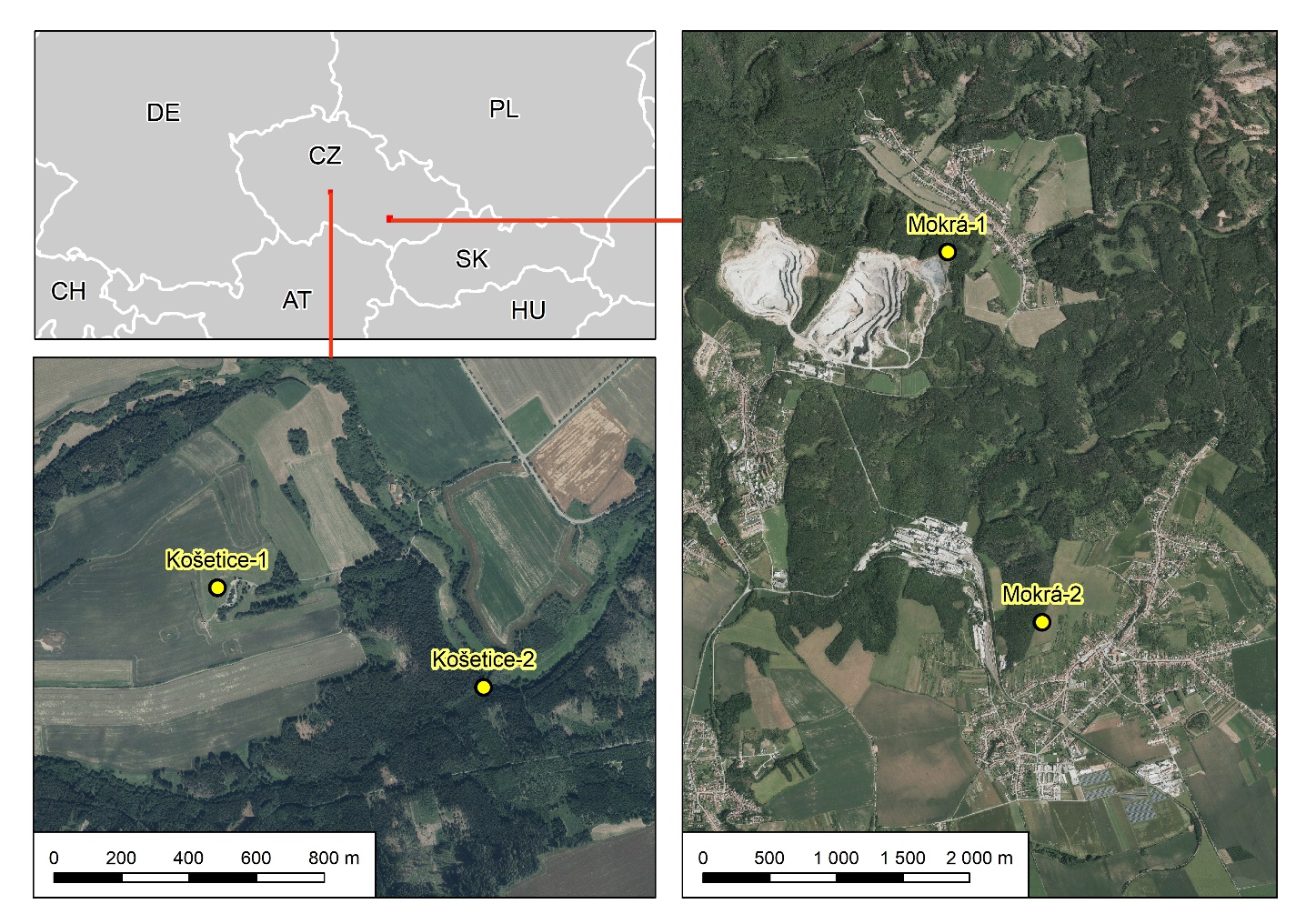


**Fig. S1** Sampling locations Košetice-1, Košetice-2, Mokrá-1 and Mokrá-2 (Satellite image taken from CENIA, Czech Environmental Information Agency, Software used: ArcGIS 9.6, ESRI, Redlands, USA)

**Table S2** Basic soil parameters of the analysed soil samples, n.d. = not determined

| Site | Lo-ca-tion # | Date of collection | Land use | Hori-zon | Depth  [cm] | Tex-ture | Soil type | Descrip-tion of vegeta-tion | Total organic carbon (TOC) [%] | pH (H_2_O) | pH (KCl) | Total N [%] | C/N | Phys. clay (≤ 2 µm) [%] | Clay (≤ 10 µm) [%] | Dust (10-50 µm) [%] | Pow-dered sand (50-100 µm) [%] | Sand (100-2000 µm) [%] | CaCO_3_ [%] |
| --- | --- | --- | --- | --- | --- | --- | --- | --- | --- | --- | --- | --- | --- | --- | --- | --- | --- | --- | --- |
| Ko-šetice | 1 | 31.08.2010 | Grass-land | A | 0-10 | Loam | Cambisol | Grass | 1.29 | n.d. | n.d. | n.d. | n.d. | n.d. | n.d. | n.d. | n.d. | n.d. | n.d. |
|  |  | 04.09.2012 |  | A |  |  |  |  | n.d. | n.d. | n.d. | n.d. | n.d. | n.d. | n.d. | n.d. | n.d. | n.d. | n.d. |
|  |  | 28.08.2013 |  | A |  |  |  |  | n.d. | n.d. | n.d. | n.d. | n.d. | n.d. | n.d. | n.d. | n.d. | n.d. | n.d. |
|  |  | 25.08.2014 |  | A |  |  |  |  | n.d. | n.d. | n.d. | n.d. | n.d. | n.d. | n.d. | n.d. | n.d. | n.d. | n.d. |
|  |  | 07.09.2015 |  | A |  |  |  |  | 2.20 | n.d. | n.d. | n.d. | n.d. | n.d. | n.d. | n.d. | n.d. | n.d. | n.d. |
|  |  | 22.08.2016 |  | A |  |  |  |  | 2.20 | n.d. | n.d. | n.d. | n.d. | n.d. | n.d. | n.d. | n.d. | n.d. | n.d. |
|  |  | 22.08.2017 |  | A |  |  |  |  | 2.48 | n.d. | n.d. | n.d. | n.d. | n.d. | n.d. | n.d. | n.d. | n.d. | n.d. |
|  | 2 | 31.08.2010 | Grass-land | A | 0-10 | Loam | Flu-visol | Grass | 5.70 | n.d. | n.d. | n.d. | n.d. | n.d. | n.d. | n.d. | n.d. | n.d. | n.d. |
|  |  | 04.09.2012 |  | A |  |  |  |  | n.d. | n.d. | n.d. | n.d. | n.d. | n.d. | n.d. | n.d. | n.d. | n.d. | n.d. |
|  |  | 28.08.2013 |  | A |  |  |  |  | n.d. | n.d. | n.d. | n.d. | n.d. | n.d. | n.d. | n.d. | n.d. | n.d. | n.d. |
|  |  | 25.08.2014 |  | A |  |  |  |  | n.d. | n.d. | n.d. | n.d. | n.d. | n.d. | n.d. | n.d. | n.d. | n.d. | n.d. |
|  |  | 08.09.2015 |  | A |  |  |  |  | 5.94 | n.d. | n.d. | n.d. | n.d. | n.d. | n.d. | n.d. | n.d. | n.d. | n.d. |
|  |  | 23.08.2016 |  | A |  |  |  |  | 4.81 | n.d. | n.d. | n.d. | n.d. | n.d. | n.d. | n.d. | n.d. | n.d. | n.d. |
|  |  | 22.08.2017 |  | A |  |  |  |  | 5.23 | n.d. | n.d. | n.d. | n.d. | n.d. | n.d. | n.d. | n.d. | n.d. | n.d. |
| Mokrá | 1 | 15.11.2006 | Grass-land | A | 0-10 | sandyloam | Cambisol | Low growth of grass, shrubs, deciduous trees (approx. 10  years) | 1.89 | n.d. | n.d. | n.d. | n.d. | n.d. | n.d. | n.d. | n.d. | n.d. | n.d. |
|  |  | 12.09.2008 |  | A |  |  |  |  | 3.19 | 6.04 | 5.08 | n.d. | n.d. | n.d. | n.d. | n.d. | n.d. | n.d. | n.d. |
|  |  | 21.04.2010 |  | A |  |  |  |  | n.d. | n.d. | n.d. | n.d. | n.d. | n.d. | n.d. | n.d. | n.d. | n.d. | n.d. |
|  |  | 01.10.2010 |  | A |  |  |  |  | 3.25 | 5.74 | 4.90 | 0.27 | 12.04 | 7.28 | 32.33 | 16.73 | 7.23 | 43.70 | 0.10 |
|  |  | 21.04.2011 |  | A |  |  |  |  | 1.85 | n.d. | n.d. | n.d. | n.d. | n.d. | n.d. | n.d. | n.d. | n.d. | n.d. |
|  |  | 01.10.2011 |  | A |  |  |  |  | 4.83 | 6.46 | 5.76 | 0.38 | 14.89 | 1.48 | 24.00 | 12.80 | 13.00 | 50.10 | 0.15 |
|  |  | 25.04.2012 |  | A |  |  |  |  | 5.05 | n.d. | n.d. | n.d. | n.d. | n.d. | n.d. | n.d. | n.d. | n.d. | n.d. |
|  |  | 11.06.2015 |  | A |  |  |  |  | 6.52 | 6.58 | 6.28 | n.d. | n.d. | n.d. | n.d. | n.d. | n.d. | n.d. | n.d. |
| Site | Lo-ca-tion # | Date of collection | Land use | Horizon | Depth  [cm] | Texture | Soil type | Descrip-tion of vegeta-tion | Total organic carbon (TOC) [%] | pH (H_2_O) | pH (KCl) | Total N [%] | C/N | Phys. clay (≤ 2 µm) [%] | Clay (≤ 10 µm) [%] | Dust (10-50 µm) [%] | Pow-dered sand (50-100 µm) [%] | Sand (100-2000 µm) [%] | CaCO_3_ [%] |
| Mokrá | 2 | 15.11.2006 | Arable land | A | 0-10 | sandy loam | Cambisol | alternation of cultures | 1.78 | n.d. | n.d. | n.d. | n.d. | n.d. | n.d. | n.d. | n.d. | n.d. | n.d. |
|  |  | 12.09.2008 |  | A |  |  |  |  | 1.97 | 6.94 | 6.30 | 0.21 | 9.37 | 5.00 | 17.70 | 13.30 | 9.60 | 59.40 | 0.13 |
|  |  | 21.04.2010 | Grass-land | A |  |  |  | Grass | 1.38 | n.d. | n.d. | n.d. | n.d. | n.d. | n.d. | n.d. | n.d. | n.d. | n.d. |
|  |  | 01.10.2010 |  | A |  |  |  |  | 1.23 | 6.52 | 5.97 | 0.20 | 6.14 | 4.56 | 24.27 | 8.60 | 10.97 | 56.17 | 0.10 |
|  |  | 21.04.2011 |  | A |  |  |  |  | 1.61 | n.d. | n.d. | n.d. | n.d. | n.d. | n.d. | n.d. | n.d. | n.d. | n.d. |
|  |  | 01.10.2011 |  | A |  |  |  |  | 1.73 | 6.85 | 6.17 | 0.21 | 11.62 | 0.57 | 13.90 | 11.90 | 12.90 | 61.30 | 0.05 |
|  |  | 25.04.2012 |  | A |  |  |  |  | 1.81 | n.d. | n.d. | n.d. | n.d. | n.d. | n.d. | n.d. | n.d. | n.d. | n.d. |
|  |  | 11.06.2015 |  | A |  |  |  |  | 3.33 | n.d. | n.d. | n.d. | n.d. | n.d. | n.d. | n.d. | n.d. | n.d. | n.d. |

**Table S3** Physico-chemical properties and degradation rates of a) PAHs, b) OPAHs and O-heterocycles, c) NPAHs; k_biodeg.1_: > 0.50: Likely to biodegrade rapidly, < 0.50: Not likely to biodegrade rapidly, k_biodeg. 2_: >4.75 – 5: Hours, >4.25 - 4.75: Hours – days, >3.75 - 4.25: Days, >3.25 - 3.75: Days – weeks, >2.75 - 3.25: Weeks, >2.25 - 2.75: Weeks – months, >1.75 - 2.25: Months, <1.75: Longer “recalcitrant”; MW = molecular weight, n.d. = not determined

a)

| Compound | Acronym | CAS # | MW  [g mol^-1^] | # of rings | Phase state ^a,b^ | log K_ow_ | k_biodeg.1_^c^ | k_biodeg.2_^d^ | log K_oc_ |
| --- | --- | --- | --- | --- | --- | --- | --- | --- | --- |
| Naphthalene | NAP | 91-20-3 | 128.18 | 2 | g | 3.30 | 1.0057 | 3.3200 | 2.96 |
| Acenaphthylene | ACY | 208-96-8 | 152.20 | 3 | g | 3.94 | 0.6751 | 3.6282 | 3.70 |
| Acenaphthene | ACE | 83-32-9 | 154.20 | 3 | g | 3.92 | 0.7835 | 3.4882 | 3.59 |
| Fluorene | FLN | 86-73-7 | 166.23 | 3 | g | 4.18 | 0.7231 | 3.5394 | 3.70 |
| Phenanthrene | PHE | 85-01-8 | 178.24 | 3 | g | 4.46 | 0.9819 | 3.2478 | 4.35 |
| Anthracene | ANT | 120-12-7 | 178.24 | 3 | g | 4.45 | 0.9819 | 3.2478 | 4.31 |
| Fluoranthene | FLT | 206-44-0 | 202.26 | 4 | g | 5.16 | -0.0060 | 2.8537 | 4.80 |
| Pyrene | PYR | 129-00-0 | 202.26 | 4 | g | 4.88 | -0.0060 | 2.8537 | 4.90 |
| Retene | RET | 483-65-8 | 234.34 | 3 | p | 6.35 | 0.7453 | 3.3726 | 5.117 |
| Benzo(b)fluorene | BBN | 243-17-4 | 216.28 | 4 | n.d. | 5.77 | 0.6992 | 3.4672 | 4.987 |
| Benzo(ghi)fluoranthene | BGF | 203-12-3 | 226.28 | 5 | n.d. | 5.52 | -0.0175 | 2.8190 | 5.256 |
| Cyclopenta(cd)pyrene | CCP | 27208-37-3 | 226.28 | 5 | n.d. | 5.70 | -0.0175 | 2.8190 | 5.248 |
| Benzo(a)anthracene | BAA | 56-55-3 | 228.30 | 4 | p | 5.76 | -0.0184 | 2.8161 | 5.248 |
| Triphenylene | TPH | 217-59-4 | 228.30 | 4 | n.d. | 5.49 | -0.0184 | 2.8161 | 5.265 |
| Chrysene | CHR | 218-01-9 | 228.30 | 4 | g | 5.81 | -0.0184 | 2.8161 | 5.256 |
| Benzo(b)fluoranthene | BBF | 205-99-2 | 252.32 | 5 | p | 5.78 | -0.0299 | 2.7815 | 5.778 |
| Benzo(j)fluoranthene | BJF | 205-82-3 | 252.32 | 5 | p | 6.11 | -0.0299 | 2.7815 | 5.778 |
| Benzo(k)fluoranthene | BKF | 207-08-9 | 252.32 | 5 | p | 6.11 | -0.0299 | 2.7815 | 5.778 |
| Benzo(e)pyrene | BEP | 192-97-2 | 252.32 | 5 | p | 6.44 | -0.0299 | 2.7815 | 5.778 |
| Benzo(a)pyrene | BAP | 50-32-8 | 252.32 | 5 | p | 6.13 | -0.0299 | 2.7815 | 5.769 |
| Perylene | PER | 198-55-0 | 252.32 | 5 | n.d. | 6.25 | -0.0299 | 2.7815 | 5.778 |
| Indeno(123-cd)pyrene | INP | 193-39-5 | 276.34 | 6 | p | 6.70 | -0.0413 | 2.7468 | 6.290 |
| Dibenz(ah)anthracene | DBA | 53-70-3 | 278.36 | 5 | p | 6.54 | -0.0423 | 2.7439 | 6.281 |
| Dibenz(ac)anthracene | DCA | 215-58-7 | 278.36 | 5 | p | 6.41 | -0.0423 | 2.7439 | 6.290 |
| Benzo(ghi)perylene | BPE | 191-24-2 | 276.34 | 6 | p | 6.63 | -0.0413 | 2.7468 | 6.290 |
| Compound | Acronym | CAS # | MW  [g mol^-1^] | # of rings | Phase state ^a,b^ | log K_ow_ | k_biodeg.1_^c^ | k_biodeg.2_^d^ | log K_oc_ |
| Anthanthrene | ATT | 191-26-4 | 276.34 | 6 | n.d. | 7.04 | -0.0413 | 2.7468 | 6.281 |
| Coronene | COR | 191-07-1 | 300.36 | 7 | p | 7.64 | -0.0527 | 2.7121 | 6.803 |

^a^if seasonal average of particulate fraction <0.5: g (gas phase), if >0.5: p (particulate phase), ^b^Data from Nežiková et al. (2021) and Tomaz et al. (2016), ^c^linear, estimate fast degradation, BioWin1 of USEPA 2019, ^c^(primary, estimate), BioWin4 of USEPA 2019

b)

| Compound | Acronym | CAS # | MW  [g mol^-1^] | # of rings | Phase state ^a,b^ | log K_ow_ | k_biodeg.1_^c^ | k_biodeg.2_^d^ | log K_oc_ |
| --- | --- | --- | --- | --- | --- | --- | --- | --- | --- |
| 1,4-Naphthoquinone | 1,4-O_2_NAP | 130-15-4 | 158.16 | 2 | g | 1.71 | 0.6859 | 3.3200 | 2.657 |
| Naphthalene-1-aldehyde | 1-(CHO)NAP | 66-77-3 | 156.19 | 2 | g | 2.89 | 0.9578 | 3.8190 | 2.079 |
| Dibenzofuran | DBF | 132-64-9 | 168.19 | 3 | g | 4.12 | 0.6675 | 3.6051 | 3.962 |
| 9-Fluorenone | 9-OFLN | 486-25-9 | 180.21 | 3 | g | 3.58 | 0.6686 | 3.5655 | 3.056 |
| 6H-Benzo(c)chromen-6-one | 6-OBCC | 2005-10-9 | 196.21 | 3 | g | 1.99 | 0.8283 | 3.7936 | 3.140 |
| 9,10-Anthraquinone | 9,10-O_2_ANT | 84-65-1 | 208.22 | 3 | g | 3.39 | 0.6621 | 3.5029 | 3.700 |
| 9,10-Phenanthroquinone | 9,10-O_2_PHE | 84-11-7 | 208.22 | 3 | p | 2.52 | 0.6484 | 3.5473 | 1.450 |
| 11H-Benzo(a)fluoren-11-one | 11-OBaFLN | 479-79-8 | 230.27 | 4 | p | 4.73 | 0.6448 | 3.4933 | 4.090 |
| 11H-Benzo(b)fluoren-11-one | 11-OBbFLN | 3074-03-1 | 230.27 | 4 | p | 4.73 | 0.6448 | 3.4933 | 4.081 |
| Benzanthrone | BAN | 82-05-3 | 230.27 | 4 | p | 4.81 | 0.6448 | 3.4933 | 4.090 |
| Benz(a)anthracene-7,12-dione | 7,12-O_2_BAA | 2498-66-0 | 258.27 | 4 | p | 4.61 | 0.6382 | 3.4307 | 4.734 |
| 5,12-Naphthacenequinone | 5,12-O_2_NAC | 1090-13-7 | 258.27 | 4 | p | 4.52 | 0.6382 | 3.4307 | 4.725 |
| 6H-Benzo(cd)pyren-6-one | 6-OBPYR | 3074-00-8 | 254.28 | 5 | p | 5.31 | -0.0240 | 2.7564 | 4.602 |

^a^if seasonal average of particulate fraction <0.5: g (gas phase), if >0.5: p (particulate phase), ^b^Data from Nežiková et al. (2021) and Tomaz et al. (2016), ^c^linear, estimate fast degradation, BioWin1 of USEPA 2019, ^c^(primary, estimate), BioWin4 of USEPA 2019

c)

| Compound | Acronym | CAS # | MW  [g mol^-1^] | # of rings | Phase state ^1,2^ | log K_ow_ | k_biodeg.1_^3^ | k_biodeg.2_ ^4^ | log K_oc_ |
| --- | --- | --- | --- | --- | --- | --- | --- | --- | --- |
| 1-Nitronaphthalene | 1-NNAP | 86-57-7 | 173.17 | 2 | g | 3.19 | 0.3601 | 3.4895 | 3.389 |
| 2-Nitronaphthalene | 2-NNAP | 581-89-5 | 173.17 | 2 | g | 3.24 | 0.3601 | 3.4895 | 3.380 |
| 3-Nitroacenaphthene | 3-NACE | 3807-77-0 | 199.21 | 3 | g | 3.97 | 0.4570 | 3.3149 | 3.901 |
| 5-Nitroacenaphthene | 5-NACE | 602-87-9 | 199.21 | 3 | g | 3.85 | 0.4570 | 3.3149 | 3.901 |
| 2-Nitrofluorene | 2-NFLN | 607-57-8 | 211.22 | 3 | g | 3.37 | 0.3966 | 3.3661 | 4.153 |
| 9-Nitroanthracene | 9-NANT | 602-60-8 | 223.23 | 3 | g | 4.78 | 0.3362 | 3.4173 | 4.423 |
| 9-Nitrophenanthrene | 9-NPHE | 954-46-1 | 223.23 | 3 | g | 4.16 | 0.3362 | 3.4173 | 4.423 |
| 3-Nitrophenanthrene | 3-NPHE | 17024-19-0 | 223.23 | 3 | g | 4.16 | 0.3362 | 3.4173 | 4.414 |
| 2-Nitrofluoranthene | 2-NFLT | 13177-29-2 | 247.26 | 4 | p | 4.29 | 0.6835 | 3.4196 | 4.935 |
| 3-Nitrofluoranthene | 3-NFLT | 892-21-7 | 247.26 | 4 | p | 4.75 | -0.3325 | 2.6804 | 4.944 |
| 1-Nitropyrene | 1-NPYR | 5522-43-0 | 247.26 | 4 | p | 5.06 | -0.3325 | 2.6804 | 4.935 |
| 7-Nitrobenzo(a)anthracene | 7-NBAA | 20268-51-3 | 273.29 | 4 | p | 5.34 | -0.3449 | 2.6428 | 5.456 |
| 6-Nitrochrysene | 6-NCHR | 7496-02-8 | 273.29 | 4 | p | 5.34 | -0.3449 | 2.6428 | 5.456 |
| 1,3-Dinitropyrene | 1,3-N_2_PYR | 75321-20-9 | 292.25 | 4 | p | 4.57 | -0.6590 | 2.5071 | 5.135 |
| 1,6-Dinitropyrene | 1,6-N_2_PYR | 42397-64-8 | 292.25 | 4 | p | 4.57 | -0.6590 | 2.5071 | 5.135 |
| 1,8-Dinitropyrene | 1,8-N_2_PYR | 42397-65-9 | 292.25 | 4 | p | 4.57 | -0.6590 | 2.5071 | 5.135 |
| 3-Nitrobenzanthrone | 3-NBAN | 17117-34-9 | 275.27 | 4 | n.d. | 4.54 | 0.3183 | 3.3200 | 4.290 |
| 6-Nitrobenzo(a)pyrene | 6-NBAP | 63041-90-7 | 297.32 | 5 | p | 5.93 | -0.3563 | 2.6082 | 5.978 |

^a^if seasonal average of particulate fraction <0.5: g (gas phase), if >0.5: p (particulate phase), ^b^Data from Nežiková et al. (2021) and Tomaz et al. (2016), ^c^linear, estimate fast degradation, BioWin1 of USEPA 2019, ^c^(primary, estimate), BioWin4 of USEPA 2019

**Table S4** SIM masses and retention times of PAHs in GC-MS analysis

| Substance | Acronym | SIM [m/z] | Retention time [min] |
| --- | --- | --- | --- |
| Naphthalene | NAP | 128.0 | 9.167 |
| Acenaphthylene | ACY | 152.0 | 12.207 |
| Acenaphthene | ACE | 154.0 | 12.613 |
| Fluorene | FLN | 166.0 | 13.952 |
| Phenanthrene | PHE | 178.0 | 16.987 |
| Anthracene | ANT | 178.0 | 17.170 |
| Fluoranthene | FLT | 202.0 | 21.628 |
| Pyrene | PYR | 202.0 | 22.574 |
| Retene | RET | 219.0 | 23.950 |
| Benzo(b)fluorene | BBN | 216.0 | 24.369 |
| Benzo(ghi])fluoranthene | BGF | 226.0 | 27.047 |
| Cyclopenta(cd)pyrene | CCP | 228.0 | 27.953 |
| Benzo(a)anthracene | BAA | 226.0 | 28.056 |
| Triphenylene | TPH | 228.0 | 28.056 |
| Chrysene | CHR | 228.0 | 28.105 |
| Benzo(b)fluoranthene | BBF | 252.0 | 32.634 |
| Benzo(j)fluoranthene | BJF | 252.0 | 32.674 |
| Benzo(k)fluoranthene | BKF | 252.0 | 32.742 |
| Benzo(e)pyrene | BEP | 252.0 | 33.733 |
| Benzo(a)pyrene (also called benzo(def)chrysene) | BAP | 252.0 | 33.931 |
| Perylene | PER | 252.0 | 34.265 |
| Indeno(123-cd)pyrene | INP | 276.0 | 38.602 |
| Dibenz(ah)anthracene | DBA | 278.0 | 38.710 |
| Dibenz(ac)anthracene | DCA | 278.0 | 38.710 |
| Benzo(ghi)perylene | BPE | 276.0 | 39.848 |
| Anthanthrene | ATT | 276.0 | 40.510 |
| Coronene | COR | 300.0 | 49.473 |

**Table S5** MRM masses and retention times of NOPAHs in GC-MS

| Substance | Acronym | Group | Target ions [m/z] | Qualifier ions [m/z] | Reten-tion time [min] |
| --- | --- | --- | --- | --- | --- |
| 1,4-Naphthoquinone | 1,4-O2NAP | OPAH | 158 > 102 | 159 > 103 | 4.06 |
| Dibenzofuran | DBF | O-heterocycle | 168 > 139 | 169 > 140 | 5.29 |
| Naphthalene-1-aldehyde | 1-(CHO)NAP | OPAH | 156 > 128 | 157 > 128 | 5.33 |
| 1-Nitronaphthalene | 1-NNAP | NPAH | 174 > 127 | 173 > 145 | 6.01 |
| 2-Nitronaphthalene | 2-NNAP | NPAH | 173 > 127 | 174 > 127 | 6.37 |
| 9-Fluorenone | 9-OFLN | OPAH | 180 > 152 | 181 > 153 | 7.25 |
| 6H-Benzo[c]chromen-6-one (also called 6H-dibenzo(bd)pyran-6-one) | 6-OBCC | O-heterocycle | 196 > 139 | 197 > 140 | 9.5 |
| 3-Nitroacenaphthene | 3-NACE | NPAH | 199 > 152 | 200 > 153 | 9.81 |
| 9,10-Anthraquinone | 9,10-O2ANT | OPAH | 208 > 152 | 209 > 153 | 9.9 |
| 9,10-Phenanthrenequinone | 9,10-O2PHE | OPAH | 180 > 152 | 209 > 153 | 10.01 |
| 5-Nitroacenaphthene | 5-NACE | NPAH | 199 > 169 | 199 > 152 | 10.34 |
| 2-Nitrofluorene | 2-NFLN | NPAH | 211 > 164 | 212 > 195 | 11.69 |
| 9-Nitroanthracene | 9-NANT | NPAH | 223 > 193 | 223 > 178 | 12.06 |
| 9-Nitrophenanthrene | 9-NPHE | NPAH | 223 > 167 | 223 > 178 | 13.11 |
| 3-Nitrophenanthrene | 3-NPHE | NPAH | 223 > 176 | 223 > 193 | 13.82 |
| Benzo[a]fluoren-11-one | 11-OBaFLN | OPAH | 230 > 202 | 231 > 203 | 15.19 |
| Benzo[b]fluoren-11-one | 11-OBbFLN | OPAH | 230 > 202 | 231 > 203 | 16.09 |
| Benzanthrone (also called 7H-Benz(de) anthracene-7-one) | BAN | OPAH | 230 > 202 | 231 > 203 | 17.28 |
| 2-Nitrofluoranthene + 3-nitrofluoranthene | 2-+3-NFLT | NPAH | 247 > 201 | 248 > 202 | 18.18 |
| Benz[a]anthracene-7,12-dione | 7,12-O2BAA | OPAH | 258 > 202 | 259 > 203 | 18.6 |
| 1-Nitropyrene | 1-NPYR | NPAH | 247 > 217 | 247 > 201 | 18.77 |
| 5,12-Naphthacenequinone | 5,12-O2NAC | OPAH | 258 > 202 | 259 > 203 | 19.77 |
| 7-Nitrobenzo[a]anthracene | 7-NBAA | NPAH | 273 > 215 | 274 > 257 | 21.44 |
| 6H-Benzo[c,d]pyren-6-one | 6-OBPYR | OPAH | 254 > 226 | 255 > 227 | 22.12 |
| 6-Nitrochrysene | 6-NCHR | NPAH | 273 > 215 | 274 > 226 | 22.65 |
| 1.3-Dinitropyrene | 1,3-N2PYR | NPAH | 292 > 188 | 292 > 176 | 23.65 |
| 1.6-Dinitropyrene | 1,6-N2PYR | NPAH | 292 > 176 | 292 > 232 | 24.4 |
| 3-Nitrobenzanthrone | 3-NBAN | NOPAH | 245 >217 | 246 > 218 | 24.77 |
| 1.8-Dinitropyrene | 1,8-N2PYR | NPAH | 292 > 176 | 292 > 232 | 24.93 |
| 6-Nitrobenzo[a]pyrene | 6-NBAP | NPAH | 297 > 239 | 297 > 224 | 26.81 |

**Table S6** Recoveries of target compounds from duplicate measurements

| Substance | Recovery [%] |
| --- | --- |
| 1-NNAP | 99 |
| 2-NNAP | 127 |
| 9-OFLN | 61 |
| 9,10-O2ANT | 171 |
| 5-NACE | 121 |
| 2-NFLN | 113 |
| 9-NANT | 106 |
| 9-NPHE | 113 |
| 3-NPHE | 105 |
| 11-OBaFLN | 162 |
| 11-OBbFLN | 154 |
| BAN | 113 |
| 2-NFLT | 101 |
| 3-NFLT | 94 |
| 7,12-O2BAA | 128 |
| 1-NPYR | 94 |
| 7-NBAA | 109 |
| 6-NCHR | 97 |
| 3-NBAN | 86 |
| 1,3-N2PYR | 81 |
| 1,6-N2PYR | 123 |
| 1,8-N2PYR | 152 |
| 6-NBAP | 111 |

**Table S7** Limits of quantification (LOQs) of a) PAHs b) OPAHs and O-heterocycles and c) NPAHs in ng g^-1^ (mLOQ: method LOQ; iLOQ: instrumental LOQ; STD: Standard deviation)

a)

| Compound | mLOQ first 10 samples with blanks 1-2^a^ | mLOQ samples all other samples with blanks 3-5 | iLOQ | LOQ (max of iLOQ & mLOQ) first 10 samples with blanks 1-2^a^ | LOQ (max of iLOQ & mLOQ) samples all other samples with blanks 3-5 |
| --- | --- | --- | --- | --- | --- |
| Naphthalene | 0.648 | 0.594 | 0.033 | 0.648 | 0.594 |
| Acenaphthylene | 0.000 | 0.000 | 0.023 | 0.023 | 0.023 |
| Acenaphthene | 0.000 | 0.000 | 0.027 | 0.027 | 0.027 |
| Fluorene | 0.684 | 0.438 | 0.020 | 0.684 | 0.438 |
| Phenanthrene | 5.241 | 4.288 | 0.025 | 5.241 | 4.288 |
| Anthracene | 0.015 | 0.520 | 0.030 | 0.030 | 0.520 |
| Fluoranthene | 3.014 | 4.695 | 0.029 | 3.014 | 4.695 |
| Pyrene | 0.756 | 3.680 | 0.031 | 0.756 | 3.680 |
| Retene | 0.034 | 14.425 | 0.068 | 0.068 | 14.425 |
| Benzo(b)fluorene | 0.000 | 0.000 | 0.081 | 0.081 | 0.081 |
| Benzo(ghi)fluoranthene | 0.010 | 0.607 | 0.021 | 0.021 | 0.607 |
| Cyclopenta(cd)pyrene | 0.000 | 0.000 | 0.030 | 0.030 | 0.030 |
| Benzo(a)anthracene | 0.015 | 0.943 | 0.029 | 0.029 | 0.943 |
| Triphenylene | 0.447 | 0.800 | 0.024 | 0.447 | 0.800 |
| Chrysene | 0.205 | 1.167 | 0.028 | 0.205 | 1.167 |
| Benzo(b)fluoranthene | 0.033 | 0.286 | 0.066 | 0.066 | 0.286 |
| Benzo(j)fluoranthene | 0.034 | 0.217 | 0.068 | 0.068 | 0.217 |
| Benzo(k)fluoranthene | 0.039 | 0.119 | 0.078 | 0.078 | 0.119 |
| Benzo(e)pyrene | 0.000 | 0.000 | 0.069 | 0.069 | 0.069 |
| Benzo(a)pyrene | 0.000 | 0.000 | 0.107 | 0.107 | 0.107 |
| Perylene | 0.000 | 0.000 | 0.090 | 0.090 | 0.090 |
| Indeno(123-cd)pyrene | 0.000 | 0.000 | 0.061 | 0.061 | 0.061 |
| Dibenz(ah)anthracene | 0.000 | 0.000 | 0.049 | 0.049 | 0.049 |
| Dibenz(ac)anthracene | 0.000 | 0.000 | 0.049 | 0.049 | 0.049 |
| Benzo(ghi)perylene | 0.000 | 0.000 | 0.051 | 0.051 | 0.051 |
| Anthanthrene | 0.202 | 1.172 | 0.058 | 0.202 | 1.172 |
| Coronene | 0.000 | 0.000 | 0.084 | 0.084 | 0.084 |

^a^First 10 samples: Kos_2010_1a,, Kos_2017_1a, Kos_2017_8a, Mokra_2015_5a, Mokra_2010Sp_2a, Mokra_2015_2a, Mokra_2010Sp_5a, Kos_2010_8a, Kos_2017_1b, Mokra_2015_2b

b)

| Compound | mLOQ sample  #1-4 with blanks 1-2^b^ | mLOQ samples >#4 with blanks 3-5 | iLOQ (average of sample-specific iLOQ) | STD of iLOQ | LOQ (max of iLOQ & mLOQ) sample  #1-4 with blanks 1-2^b^ | LOQ (max of iLOQ & mLOQ) samples >#4 with blanks 3-5 |
| --- | --- | --- | --- | --- | --- | --- |
| 1,4-Naphthoquinone | 0.17 | 0.04 | 0.016 | 0.01 | 0.17 | 0.04 |
| Naphthalene-1-aldehyde | 0.10 | 0.02 | 0.005 | 0.00 | 0.10 | 0.02 |
| Dibenzofuran | 0.35 | 0.12 | 0.001 | 0.00 | 0.35 | 0.12 |
| 9-Fluorenone | 1.16 | 0.51 | 0.003 | 0.00 | 1.16 | 0.51 |
| 6H-Benzo(c)chromen-6-one | 0.21 | 0.32 | 0.009 | 0.01 | 0.21 | 0.32 |
| 9,10-Anthraquinone | 1.63 | 4.67 | 0.012 | 0.01 | 1.63 | 4.67 |
| 9,10-Phenanthroquinone | 0.00 | 0.00 | 2.844 | 1.99 | 2.84 | 2.84 |
| 11H-Benzo(a)fluoren-11-one | 0.10 | 0.16 | 0.026 | 0.05 | 0.10 | 0.16 |
| 11H-Benzo(b)fluoren-11-one | 0.06 | 0.05 | 0.022 | 0.05 | 0.06 | 0.05 |
| Benzanthrone | 0.05 | 0.13 | 0.020 | 0.04 | 0.05 | 0.13 |
| Benz(a)anthracene-7,12-dione | 0.06 | 0.03 | 0.021 | 0.05 | 0.06 | 0.03 |
| 5,12-Naphthacenequinone | 0.00 | 0.00 | 0.015 | 0.03 | 0.01 | 0.01 |
| 6H-Benzo(cd)pyren-6-one | 0.01 | 0.02 | 0.012 | 0.03 | 0.01 | 0.02 |

^b^First 4 samples: Kos_2010_8a, Kos_2017_1b, Mokra_2015_2b, Kos_2017_1c

c)

| Compound | mLOQ sample  #1-4 with blanks 1-2^b^ | mLOQ samples >#4 with blanks 3-5 | iLOQ (average of sample-specific iLOQ) | STD of iLOQ | LOQ (max of iLOQ & mLOQ) sample  #1-4 with blanks 1-2^b^ | LOQ (max of iLOQ & mLOQ) samples >#4 with blanks 3-5 |
| --- | --- | --- | --- | --- | --- | --- |
| 1-Nitronaphthalene | 0.072 | 0.104 | 0.009 | 0.005 | 0.072 | 0.104 |
| 2-Nitronaphthalene | 0.055 | 0.091 | 0.004 | 0.003 | 0.055 | 0.091 |
| 3-Nitroacenaphthene | 0.000 | 0.000 | 0.178 | 0.127 | 0.178 | 0.178 |
| 5-Nitroacenaphthene | 0.000 | 0.000 | 0.010 | 0.006 | 0.010 | 0.010 |
| 2-Nitrofluorene | 0.000 | 0.000 | 0.002 | 0.001 | 0.002 | 0.002 |
| 9-Nitroanthracene | 0.000 | 0.000 | 0.049 | 0.023 | 0.049 | 0.049 |
| 9-Nitrophenanthrene | 0.000 | 0.000 | 0.041 | 0.017 | 0.041 | 0.041 |
| 3-Nitrophenanthrene | 0.000 | 0.000 | 0.015 | 0.007 | 0.015 | 0.015 |
| 2-+3-Nitrofluoranthene | 0.012 | 0.026 | 0.003 | 0.002 | 0.012 | 0.026 |
| 1-Nitropyrene | 0.011 | 0.018 | 0.004 | 0.003 | 0.011 | 0.018 |
| 7-Nitrobenzo(a)anthracene | 0.000 | 0.000 | 0.009 | 0.025 | 0.009 | 0.009 |
| 6-Nitrochrysene | 0.000 | 0.000 | 0.007 | 0.018 | 0.007 | 0.007 |
| 1,3-Dinitropyrene | 0.000 | 0.000 | 0.001 | 0.001 | 0.001 | 0.001 |
| 1,6-Dinitropyrene | 0.000 | 0.000 | 0.003 | 0.002 | 0.003 | 0.003 |
| 1,8-Dinitropyrene | 0.000 | 0.000 | 0.003 | 0.002 | 0.003 | 0.003 |
| 3-Nitrobenzanthrone | 0.000 | 0.000 | 0.488 | 0.495 | 0.488 | 0.488 |
| 6-Nitrobenzo(a)pyrene | 0.017 | 0.011 | 0.007 | 0.009 | 0.017 | 0.011 |

^b^First 4 samples: Kos_2010_8a, Kos_2017_1b, Mokra_2015_2b, Kos_2017_1c

**Table S8** Repeatability (CoVs) of individual PACs, a) PAHs, b) OPAHs and O-heterocycles and c) NPAHs, of the 11 samples measured in duplicate or triplicate (STD: Standard deviation)

a)

| Compound | Average of CoV [%] | STD of CoV [%] | Compound | Average of CoV [%] | STD of CoV [%] |
| --- | --- | --- | --- | --- | --- |
| Naphthalene | 33 | 23 | **Benzo(b)fluoranthene** | 12 | 9 |
| Acenaphthylene | 15 | 18 | **Benzo(j)fluoranthene** | 19 | 11 |
| Acenaphthene | 21 | 31 | **Benzo(k)fluoranthene** | 12 | 9 |
| Fluorene | 41 | 40 | **Benzo(e)pyrene** | 14 | 12 |
| Phenanthrene | 31 | 34 | **Benzo(a)pyrene** | 14 | 10 |
| Anthracene | 35 | 40 | **Perylene** | 16 | 12 |
| Fluoranthene | 36 | 40 | **Indeno(123-cd)pyrene** | 12 | 11 |
| Pyrene | 32 | 30 | **Dibenz(ah)anthracene** | 12 | 10 |
| Retene | 33 | 21 | **Dibenz(ac)anthracene** | 16 | 8 |
| Benzo(b)fluorene | 27 | 35 | **Benzo(ghi)perylene** | 15 | 10 |
| Benzo(ghi)fluoranthene | 20 | 21 | **Anthanthrene** | 28 | 28 |
| Cyclopenta(cd)pyrene | - | - | **Coronene** | 20 | 15 |
| Benzo(a)anthracene | 26 | 20 | **Σ_16_US-EPA PAHs** | 15 | 12 |
| Triphenylene | 22 | 22 | **Σ_27_PAHs** | 15 | 12 |
| Chrysene | 21 | 18 |  | | |

b)

| Compound | Average of CoV [%] | STD of CoV [%] |
| --- | --- | --- |
| 1,4-Naphthoquinone | 37 | 32 |
| Naphthalene-1-aldehyde | 35 | 27 |
| Dibenzofuran | 27 | 19 |
| 9-Fluorenone | 48 | 43 |
| 6H-Benzo(c)chromen-6-one | 38 | 34 |
| 9,10-Anthraquinone | 135^a^ | 52 |
| 9,10-Phenanthroquinone | - | - |
| 11H-Benzo(a)fluoren-11-one | 31 | 24 |
| 11H-Benzo(b)fluoren-11-one | 30 | 25 |
| Benzanthrone | 39 | 20 |
| Benz(a)anthracene-7,12-dione | 27 | 21 |
| 5,12-Naphthacenequinone | 21 | 16 |
| 6H-Benzo(cd)pyren-6-one | 24 | 14 |
| Σ13OPAHs (11 OPAHs+2 O-heterocycles) | 22 | 17 |
| Σ11OPAHs | 23 | 19 |

^a^High value since often close to LOQ, which is relatively high, and if <LOQ calculated with 0 ng g^-1^ since detection frequency is <25 %.

c)

| Compound | Average of CoV [%] | STD of CoV [%] |
| --- | --- | --- |
| 1-Nitronaphthalene | 31 | 31 |
| 2-Nitronaphthalene | 38 | 45 |
| 2-+3-Nitrofluoranthene | 26 | 22 |
| 1-Nitropyrene | 36 | 34 |
| 6-Nitrobenzo(a)pyrene | 28 | 23 |
| Σ6NPAHs | 27 | 25 |


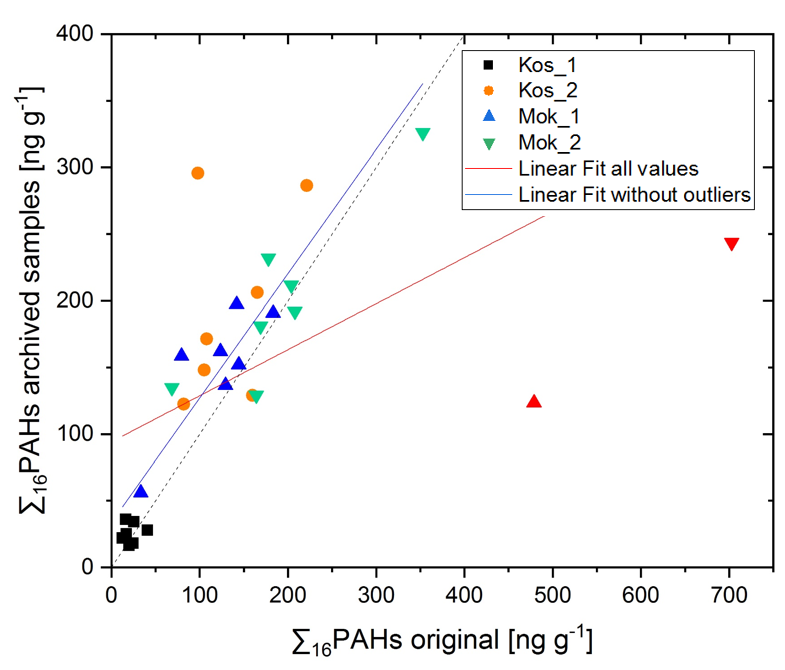


**Fig. S2** ∑_16_PAHs concentration from measurement done temporarily close to the sampling date, c_original_, compared to ∑_16_PAHs concentration from re-measurement, c_archived_ _sample_, of the archived soil samples. Linear fit with all values (red fit line with equation c_archived sample_ = 0.35 c_original_ + 94.25; R = 0.58, p<0.05) and linear fit without two outlier values from Mokrá-1 and Mokrá-2 (marked in red) (blue fit line with equation c_archived sample_ = 0.93 c_original_ + 33.77; R = 0.86, p<0.05 )

a)


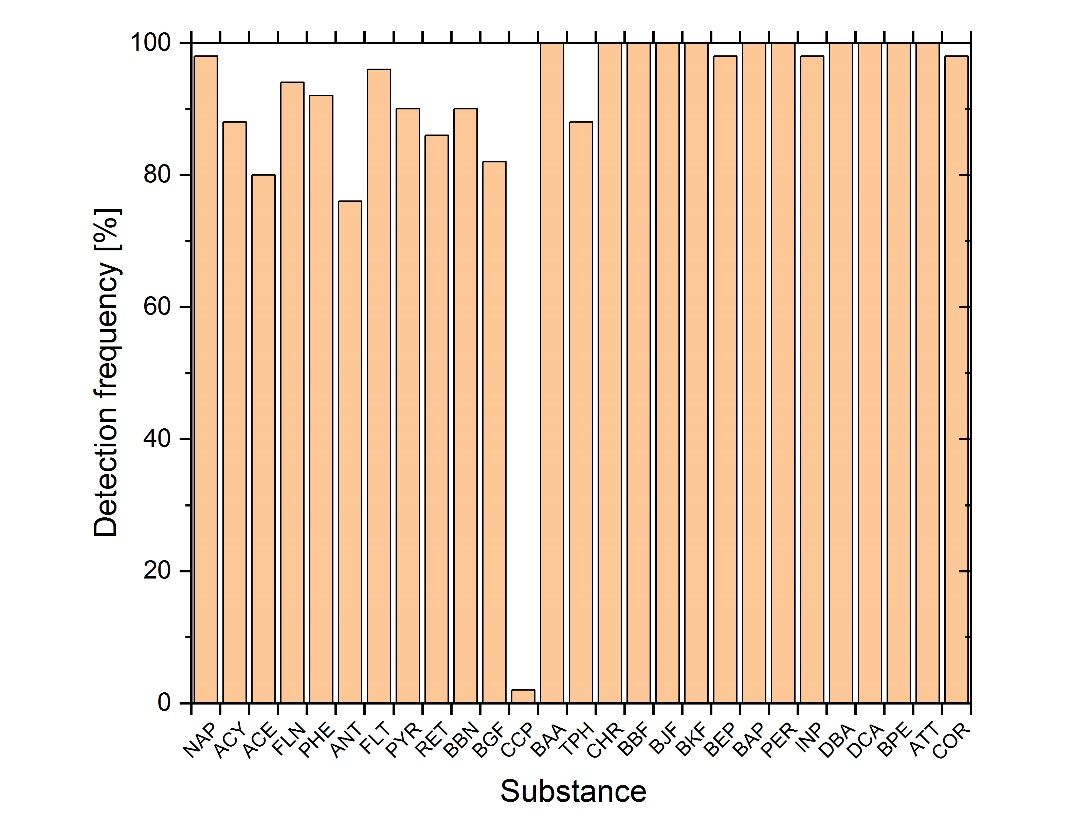


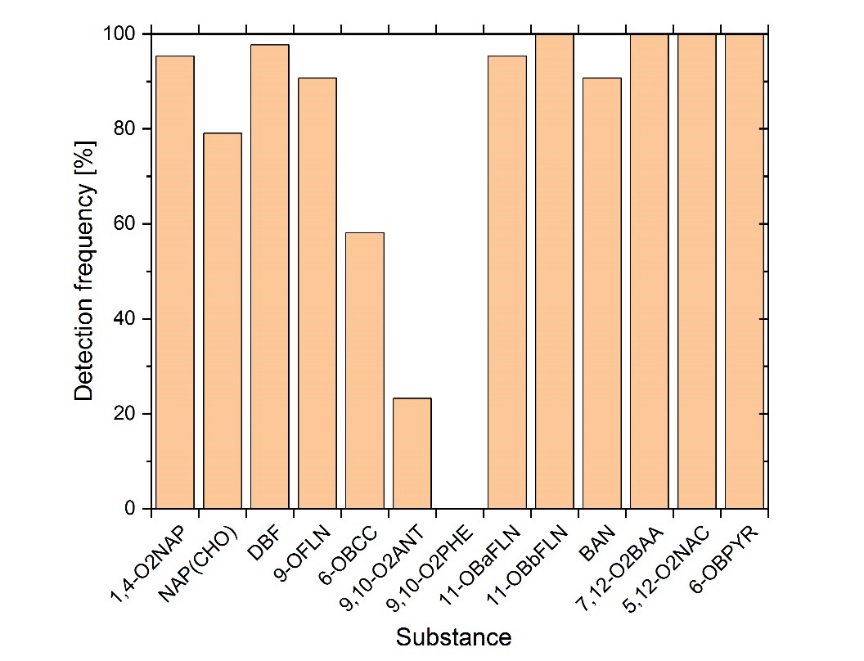

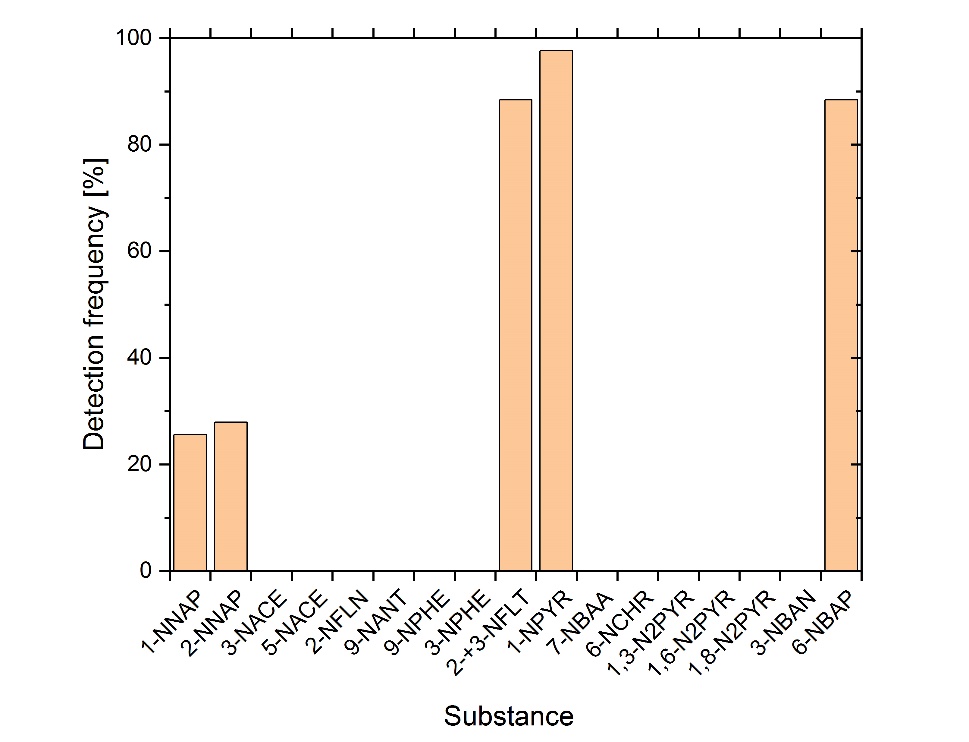


c)

b)

**Fig. S3** Detection frequency of a) PAHs, b) OPAHs and O-heterocycles and c) NPAHs using the sum of the average of blanks and 1 × standard deviation of blanks as LOQ


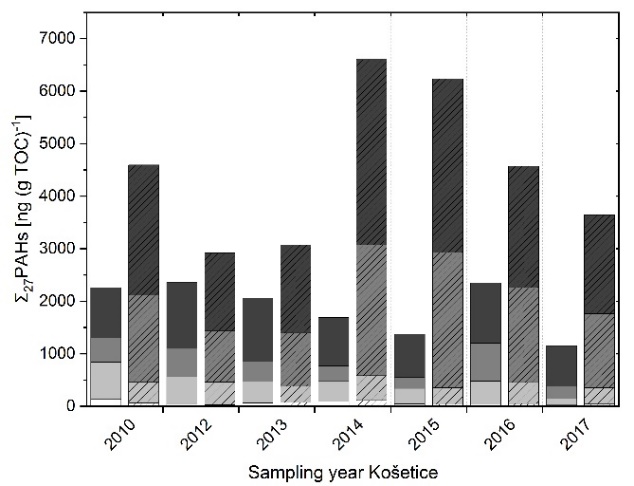

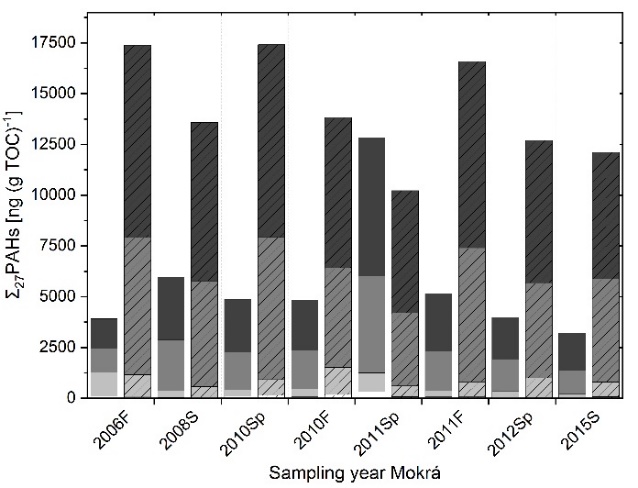


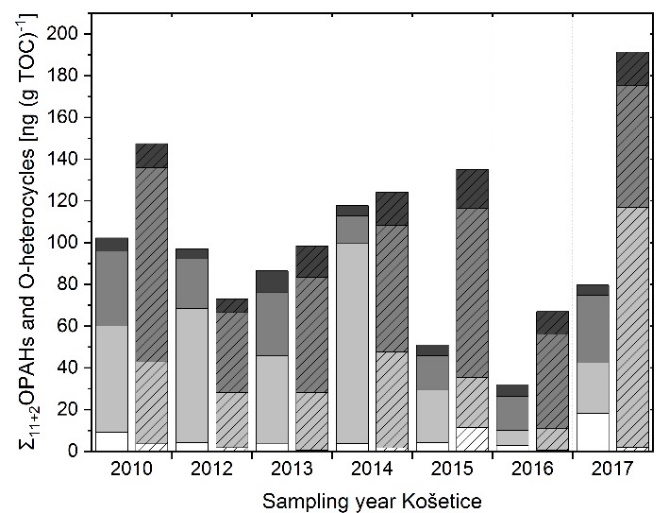

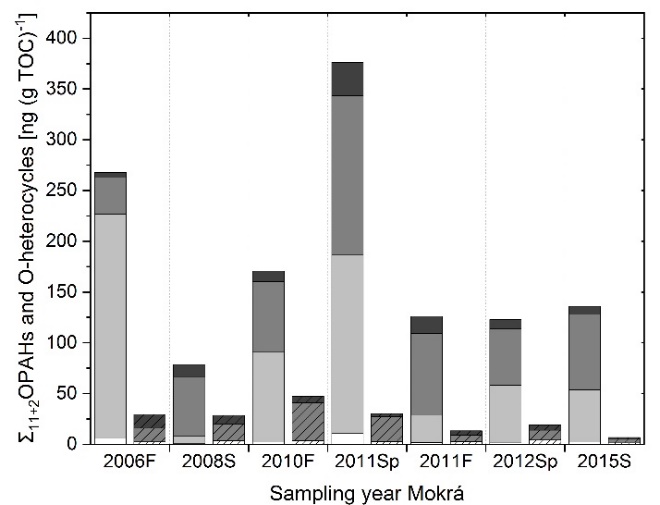


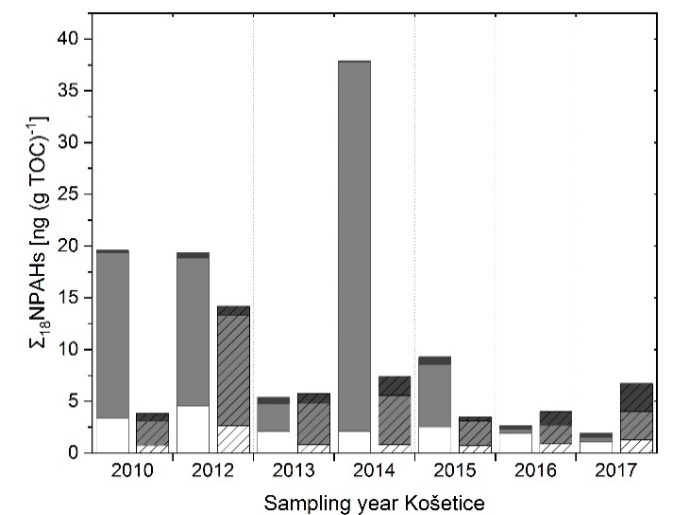

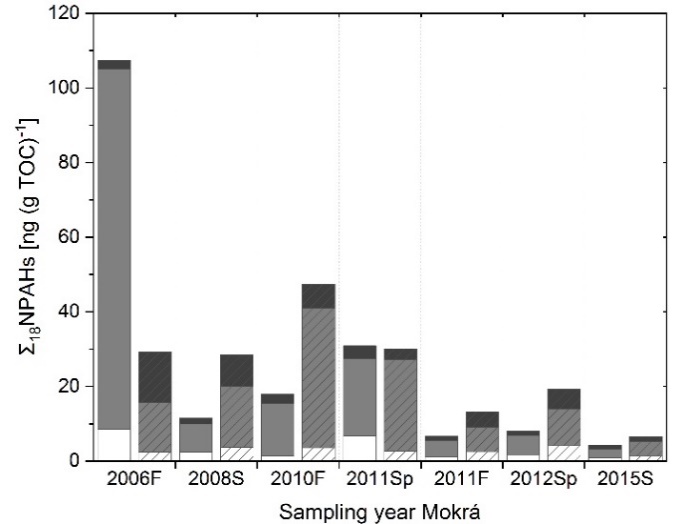


**Fig. S4** Concentration normalized for TOC content in ng (g TOC)^-1^ of A & B: Σ_27_PAHs split into 2-ring (white), 3-ring (light grey), 4-ring (grey) and 5-7-ring PAHs (dark grey), A: at Košetice-1 (plain) and Košetice-2 (dashed); B: at Mokrá-1 (plain) and Mokrá-2 (dashed); C & D: Σ_11+2_OPAHs and O-heterocycles split into 2-ring (white), 3-ring (light grey), 4-ring (grey) and 5-ring OPAHs (dark grey), C: at Košetice-1 (plain) and Košetice-2 (dashed); D: at Mokrá-1 (plain) and Mokrá-2 (dashed); E & F: Σ_18_NPAHs split into 2-ring NPAHs (white), 3-ring NPAHs (light grey), 4-ring NPAHs (grey) and 5-ring NPAHs (dark grey), E: at Košetice-1 (plain) and Košetice-2 (dashed); F: at Mokrá-1 (plain) and Mokrá-2 (dashed); (F: Fall; Sp: Spring; S: Summer).


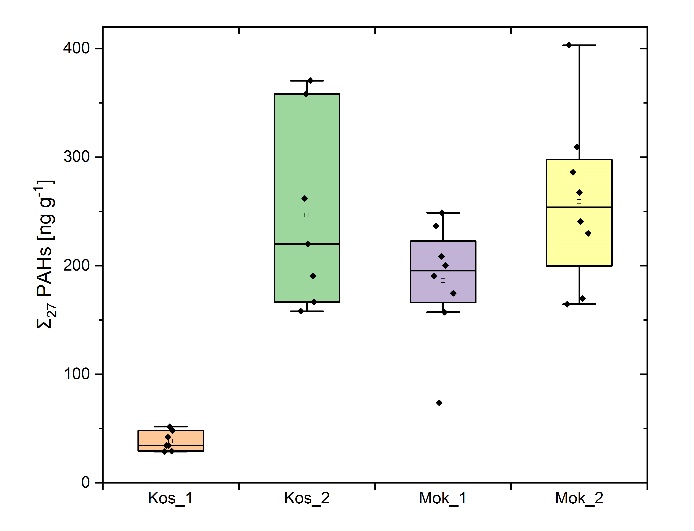

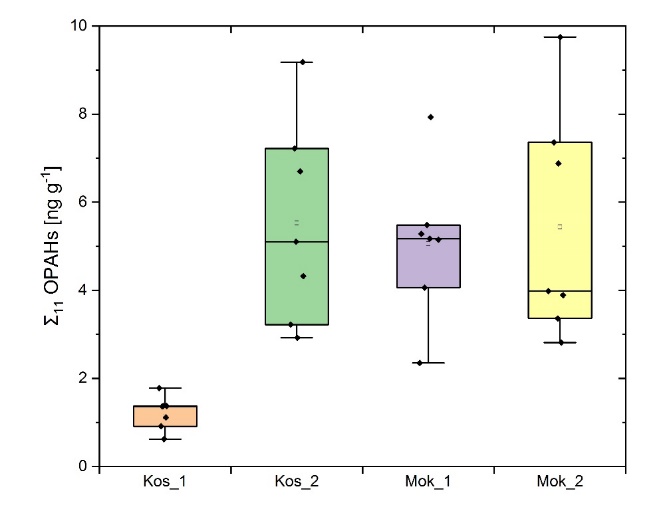

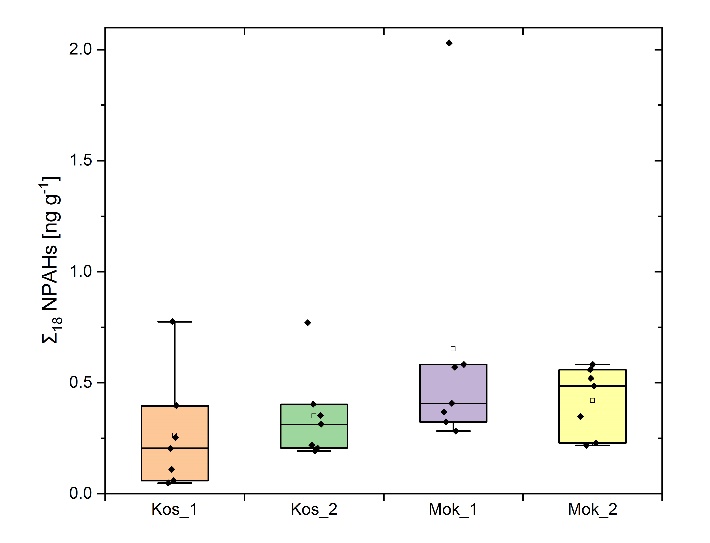

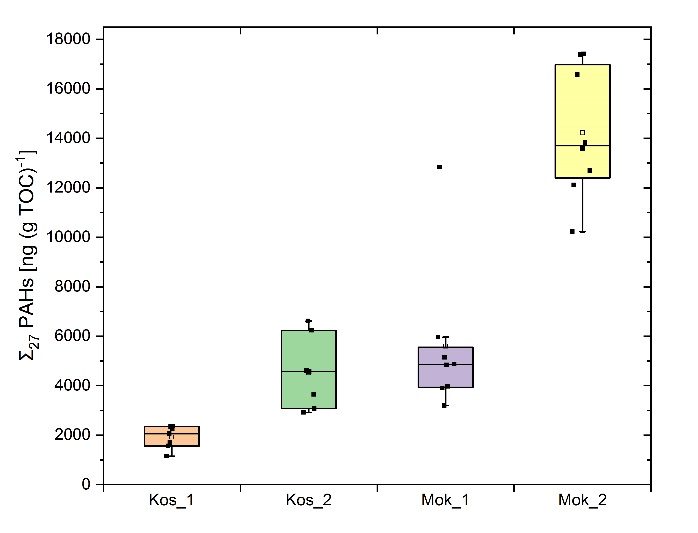


c)

d)

b)

a)


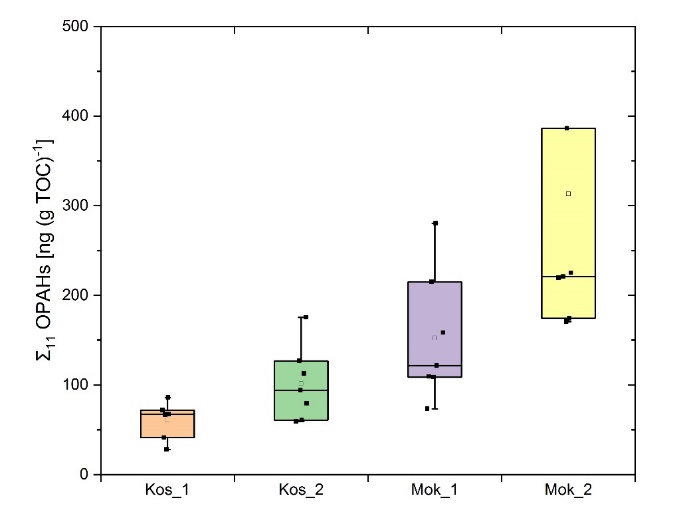

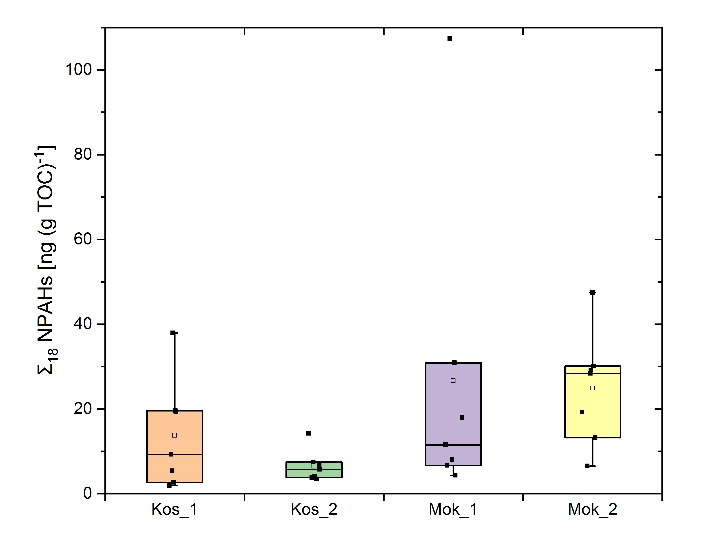


f)

e)

**Fig. S5** Box-and-whisker-plot of location average concentrations of Σ27PAHs (a and d), Σ_11_OPAHs (b and e) and Σ_18_NPAHs (c and f) in ng g^-1^ (a-c) and normalized for TOC content in ng (g TOC)^-1^ (d-f) (empty square: Mean value; Filled squares: Measurement points; Filled box with extra borders: Interquartile range (IQR) bound by the 75th and 25th percentile and range of 1.5 IQR; Horizontal line: Median)

**Table S9** Concentration of a) Σ_11_OPAHs, Σ_2_O-heterocycles, Σ_16_PAHs, Σ_27_PAHs in ng g^-1^ and b) ∑_18_NPAHs and individual NPAHs in pg g^-1^ (F: Fall; Sp: Spring; S: Summer). Values <LOQ were replaced by LOQ/2 if the detection frequency was >25 % (**Fig. S3**), else replaced by 0 ng g^-1^

a)

| Location | Year & season | Σ_11_OPAHs | Σ_2_O-heterocycles | Σ_16_PAHs | Σ_27_PAHs |
| --- | --- | --- | --- | --- | --- |
| Košetice-1 | 2010 | 1.11 | 0.21 | 22 | 29 |
|  | 2012 | 1.37 | 0.61 | 36 | 48 |
|  | 2013 | 1.36 | 0.40 | 28 | 42 |
|  | 2014 | 1.37 | 1.03 | 25 | 35 |
|  | 2015 | 0.91 | 0.22 | 18 | 34 |
|  | 2016 | 0.62 | 0.09 | 34 | 52 |
|  | 2017 | 1.78 | 0.19 | 17 | 29 |
| Košetice-2 | 2010 | 7.22 | 1.18 | 206 | 262 |
|  | 2012 | 3.22 | 0.74 | 122 | 158 |
|  | 2013 | 4.32 | 1.01 | 129 | 167 |
|  | 2014 | 5.10 | 1.63 | 286 | 358 |
|  | 2015 | 6.70 | 0.74 | 296 | 370 |
|  | 2016 | 2.92 | 0.30 | 171 | 220 |
|  | 2017 | 9.18 | 0.83 | 148 | 191 |
| Mokrá-1 | 2006F | 4.06 | 1.00 | 56 | 74 |
|  | 2008S | 2.35 | 0.16 | 152 | 190 |
|  | 2010Sp | - | - | 137 | 175 |
|  | 2010F | 5.15 | 0.40 | 123 | 157 |
|  | 2011Sp | 5.17 | 1.77 | 191 | 237 |
|  | 2011F | 5.28 | 0.81 | 197 | 249 |
|  | 2012Sp | 5.48 | 0.72 | 159 | 200 |
|  | 2015S | 7.93 | 0.94 | 162 | 209 |
| Mokrá-2 | 2006F | 6.88 | 0.41 | 244 | 309 |
|  | 2008S | 3.36 | 0.41 | 212 | 267 |
|  | 2010Sp | - | - | 192 | 241 |
|  | 2010F | 9.75 | 1.01 | 135 | 170 |
|  | 2011Sp | 2.81 | 0.58 | 129 | 165 |
|  | 2011F | 3.89 | 0.30 | 232 | 286 |
|  | 2012Sp | 3.98 | 0.16 | 181 | 230 |
|  | 2015S | 7.36 | 0.58 | 326 | 403 |

b)

| Location | Year | Σ_18_NPAHs | 1-NNAP | 2-NNAP | 2-+3-NFLT | 1-NPYR | 6-NBAP |
| --- | --- | --- | --- | --- | --- | --- | --- |
| Košetice-1 | 2010 | 255.1 | 14.5 | 28.7 | 5.3 | 201.2 | 3.5 |
|  | 2012 | 403.5 | 14.9 | 78.4 | 68.8 | 222.5 | 11.5 |
|  | 2013 | 112.9 | 14.5 | 28.7 | 33.8 | 20.1 | 12.9 |
|  | 2014 | 779.3 | 14.5 | 28.8 | 41.5 | 686.4 | 3.5 |
|  | 2015 | 209.8 | 25.9 | 29.1 | 49.1 | 83.4 | 17.1 |
|  | 2016 | 59.6 | 14.1 | 28.0 | 5.2 | 2.9 | 8.7 |
|  | 2017 | 48.8 | 12.4 | 14.7 | 4.6 | 5.1 | 11.4 |
| Košetice-2 | 2010 | 220.1 | 14.5 | 28.7 | 30.8 | 100.7 | 43.7 |
|  | 2012 | 773.6 | 14.7 | 129.2 | 31.8 | 544.9 | 49.4 |
|  | 2013 | 318.1 | 14.5 | 28.8 | 50.5 | 165.6 | 53.1 |
|  | 2014 | 414.4 | 14.7 | 29.2 | 46.9 | 210.2 | 101.8 |
|  | 2015 | 210.9 | 14.8 | 29.3 | 46.7 | 93.5 | 21.6 |
|  | 2016 | 193.3 | 14.3 | 28.4 | 46.0 | 40.7 | 63.2 |
|  | 2017 | 355.2 | 14.3 | 52.9 | 53.2 | 90.9 | 140.4 |
| Mokrá-1 | 2006F | 2031.4 | 71.6 | 89.2 | 5.3 | 1820.7 | 43.2 |
|  | 2008S | 372.3 | 50.4 | 28.2 | 29.2 | 208.8 | 51.0 |
|  | 2010F | 583.0 | 14.6 | 28.9 | 46.5 | 412.6 | 79.7 |
|  | 2011Sp | 577.4 | 48.1 | 77.4 | 86.3 | 293.9 | 64.5 |
|  | 2011F | 326.0 | 29.8 | 28.6 | 62.1 | 143.5 | 59.4 |
|  | 2012Sp | 409.3 | 25.6 | 50.3 | 51.7 | 218.7 | 60.6 |
|  | 2015S | 288.9 | 12.3 | 41.0 | 85.1 | 69.0 | 74.8 |
| Mokrá-2 | 2006F | 523.9 | 14.6 | 28.9 | 37.6 | 197.1 | 241.3 |
|  | 2008S | 563.2 | 22.4 | 49.9 | 68.1 | 252.7 | 165.8 |
|  | 2010F | 583.0 | 14.6 | 28.9 | 46.5 | 412.6 | 79.7 |
|  | 2011Sp | 489.0 | 14.3 | 28.4 | 76.0 | 319.6 | 46.4 |
|  | 2011F | 229.2 | 14.3 | 28.4 | 37.4 | 76.2 | 72.1 |
|  | 2012Sp | 348.4 | 45.5 | 28.9 | 20.2 | 158.0 | 95.3 |
|  | 2015S | 229.1 | 14.5 | 28.7 | 69.5 | 60.0 | 45.1 |

**Table S10** Same as Table S9 but normalized for soil TOC content in ng (g TOC)^-1^

a)

| Location | Year & season | Σ_11_OPAHs | Σ_2_O-heterocycles | Σ_16_PAHs | Σ_27_PAHs |
| --- | --- | --- | --- | --- | --- |
| Košetice-1 | 2010 | 86.0 | 16.3 | 1704 | 2262 |
|  | 2012 | 67.2 | 29.9 | 1764 | 2360 |
|  | 2013 | 66.7 | 19.8 | 1361 | 2059 |
|  | 2014 | 67.2 | 50.5 | 1228 | 1689 |
|  | 2015 | 41.3 | 9.8 | 826 | 1560 |
|  | 2016 | 28.0 | 4.0 | 1553 | 2351 |
|  | 2017 | 71.9 | 7.8 | 671 | 1153 |
| Košetice-2 | 2010 | 126.8 | 20.8 | 3620 | 4597 |
|  | 2012 | 59.5 | 13.6 | 2258 | 2917 |
|  | 2013 | 79.6 | 18.7 | 2380 | 3073 |
|  | 2014 | 94.1 | 30.0 | 5284 | 6609 |
|  | 2015 | 112.8 | 12.5 | 4979 | 6235 |
|  | 2016 | 60.7 | 6.3 | 3562 | 4574 |
|  | 2017 | 175.5 | 15.8 | 2832 | 3643 |
| Mokrá-1 | 2006F | 215.0 | 52.7 | 2959 | 3902 |
|  | 2008S | 73.5 | 5.1 | 4765 | 5967 |
|  | 2010Sp | - | - | 3808 | 4868 |
|  | 2010F | 158.3 | 12.4 | 3796 | 4837 |
|  | 2011Sp | 280.4 | 95.9 | 10337 | 12828 |
|  | 2011F | 109.3 | 16.8 | 4080 | 5142 |
|  | 2012Sp | 108.6 | 14.2 | 3140 | 3968 |
|  | 2015S | 121.6 | 14.5 | 2483 | 3199 |
| Mokrá-2 | 2006F | 386.2 | 23.0 | 13692 | 17385 |
|  | 2008S | 170.7 | 21.1 | 10771 | 13585 |
|  | 2010Sp | - | - | 13891 | 17411 |
|  | 2010F | 794.3 | 82.5 | 10976 | 13818 |
|  | 2011Sp | 174.4 | 36.0 | 8022 | 10220 |
|  | 2011F | 225.2 | 17.4 | 13440 | 16576 |
|  | 2012Sp | 219.6 | 8.9 | 9994 | 12693 |
|  | 2015S | 220.9 | 17.3 | 9794 | 12107 |

b)

| Location | Year & season | Σ_18_ NPAHs | 1-NNAP | 2-NNAP | 2-+3-NFLT | 1-NPYR | 6-NBAP |
| --- | --- | --- | --- | --- | --- | --- | --- |
| Košetice-1 | 2010 | 19.74 | 1.12 | 2.22 | 0.41 | 15.57 | 0.27 |
|  | 2012 | 19.75 | 0.73 | 3.84 | 3.37 | 10.89 | 0.56 |
|  | 2013 | 5.52 | 0.71 | 1.40 | 1.65 | 0.99 | 0.63 |
|  | 2014 | 38.15 | 0.71 | 1.41 | 2.03 | 33.60 | 0.17 |
|  | 2015 | 9.54 | 1.18 | 1.32 | 2.23 | 3.79 | 0.78 |
|  | 2016 | 2.71 | 0.64 | 1.27 | 0.24 | 0.13 | 0.39 |
|  | 2017 | 1.97 | 0.50 | 0.59 | 0.19 | 0.21 | 0.46 |
| Košetice-2 | 2010 | 3.86 | 0.25 | 0.50 | 0.54 | 1.77 | 0.77 |
|  | 2012 | 14.27 | 0.27 | 2.38 | 0.59 | 10.05 | 0.91 |
|  | 2013 | 5.87 | 0.27 | 0.53 | 0.93 | 3.05 | 0.98 |
|  | 2014 | 7.65 | 0.27 | 0.54 | 0.87 | 3.88 | 1.88 |
|  | 2015 | 3.55 | 0.25 | 0.49 | 0.79 | 1.57 | 0.36 |
|  | 2016 | 4.02 | 0.30 | 0.59 | 0.96 | 0.85 | 1.31 |
|  | 2017 | 6.79 | 0.27 | 1.01 | 1.02 | 1.74 | 2.68 |
| Mokrá-1 | 2006F | 107.48 | 3.79 | 4.72 | 0.28 | 96.33 | 2.29 |
|  | 2008S | 11.67 | 1.58 | 0.88 | 0.91 | 6.54 | 1.60 |
|  | 2010F | 17.93 | 0.45 | 0.89 | 1.43 | 12.69 | 2.45 |
|  | 2011Sp | 31.29 | 2.61 | 4.19 | 4.68 | 15.92 | 3.50 |
|  | 2011F | 6.74 | 0.62 | 0.59 | 1.28 | 2.97 | 1.23 |
|  | 2012Sp | 8.11 | 0.51 | 1.00 | 1.02 | 4.33 | 1.20 |
|  | 2015S | 4.43 | 0.19 | 0.63 | 1.31 | 1.06 | 1.15 |
| Mokrá-2 | 2006F | 29.43 | 0.82 | 1.62 | 2.11 | 11.07 | 13.56 |
|  | 2008S | 28.63 | 1.14 | 2.54 | 3.46 | 12.85 | 8.43 |
|  | 2010F | 47.50 | 1.19 | 2.35 | 3.79 | 33.61 | 6.49 |
|  | 2011Sp | 30.37 | 0.89 | 1.76 | 4.72 | 19.85 | 2.88 |
|  | 2011F | 13.28 | 0.83 | 1.65 | 2.17 | 4.42 | 4.18 |
|  | 2012Sp | 19.25 | 2.51 | 1.60 | 1.11 | 8.73 | 5.27 |
|  | 2015S | 6.88 | 0.43 | 0.86 | 2.09 | 1.80 | 1.35 |

**Table S11** Concentrations of PAHs in soil samples from a) Košetice-1, b) Košetice-2, c) Mokrá-1 and d) Mokrá-2 in ng g^-1^ (F:Fall; Sp:Spring; S:Summer; STD: Standard deviation)

a)

| Compound | 2010 | 2012 | 2013 | 2014 | 2015 | 2016 | 2017 | Mean | STD | Median |
| --- | --- | --- | --- | --- | --- | --- | --- | --- | --- | --- |
| NAP | 1.70 | 0.99 | 1.36 | 1.80 | 1.03 | 0.83 | 0.55 | **1.18** | 0.46 | 1.03 |
| ACY | 0.01 | 0.20 | 0.19 | 0.22 | 0.21 | 0.17 | 0.01 | **0.15** | 0.09 | 0.19 |
| ACE | 0.01 | 0.11 | 0.13 | 0.14 | 0.01 | 0.01 | 0.05 | **0.07** | 0.06 | 0.05 |
| FLN | 0.53 | 0.79 | 0.86 | 0.82 | 0.67 | 0.21 | 0.21 | **0.59** | 0.28 | 0.67 |
| PHE | 7.07 | 6.50 | 3.36 | 4.98 | 1.48 | 2.21 | 0.82 | **3.77** | 2.46 | 3.36 |
| ANT | 0.11 | 0.16 | 0.16 | 0.16 | 0.16 | 0.41 | 0.08 | **0.18** | 0.11 | 0.16 |
| FLT | 2.12 | 3.82 | 2.38 | 2.65 | 1.51 | 5.49 | 1.81 | **2.82** | 1.39 | 2.38 |
| PYR | 1.78 | 2.37 | 1.74 | 0.55 | 0.90 | 5.19 | 1.42 | **1.99** | 1.53 | 1.74 |
| RET | 1.42 | 2.69 | 3.54 | 1.75 | 3.94 | 6.69 | 1.85 | **3.13** | 1.83 | 2.69 |
| BBN | 0.32 | 0.52 | 0.40 | 0.44 | 0.15 | 0.04 | 0.35 | **0.32** | 0.17 | 0.35 |
| BGF | 0.21 | 0.14 | 0.14 | 0.14 | 0.14 | 0.62 | 0.22 | **0.23** | 0.17 | 0.14 |
| CCP | <0.03 | <0.03 | <0.03 | <0.03 | <0.03 | <0.03 | 0.15 | **0.02** | 0.05 | <0.03 |
| BAA | 0.71 | 1.84 | 1.47 | 0.96 | 0.97 | 2.40 | 0.89 | **1.32** | 0.61 | 0.97 |
| TPH | 0.27 | 0.42 | 0.28 | 0.12 | 0.12 | 0.52 | 0.24 | **0.28** | 0.15 | 0.27 |
| CHR | 0.77 | 2.00 | 1.48 | 1.06 | 1.03 | 2.24 | 1.05 | **1.38** | 0.56 | 1.06 |
| BBF | 1.89 | 4.49 | 3.72 | 3.36 | 2.81 | 4.38 | 2.55 | **3.31** | 0.96 | 3.36 |
| BJF | 0.90 | 1.78 | 1.29 | 0.82 | 0.85 | 1.38 | 1.41 | **1.20** | 0.36 | 1.29 |
| BKF | 0.67 | 1.45 | 1.20 | 0.91 | 0.89 | 1.39 | 0.80 | **1.04** | 0.30 | 0.91 |
| BEP | 1.87 | 3.81 | 4.32 | 2.78 | 3.74 | 4.17 | 3.36 | **3.44** | 0.86 | 3.74 |
| BAP | 1.61 | 3.47 | 2.88 | 2.30 | 2.02 | 3.38 | 1.82 | **2.50** | 0.75 | 2.30 |
| PER | 0.35 | 0.75 | 0.67 | 0.62 | 0.44 | 0.78 | 0.41 | **0.57** | 0.17 | 0.62 |
| INP | 1.36 | 2.86 | 2.79 | 2.20 | 1.73 | 2.49 | 1.63 | **2.15** | 0.59 | 2.20 |
| DBA | 0.37 | 0.90 | 0.83 | 0.89 | 0.72 | 0.99 | 1.09 | **0.83** | 0.23 | 0.89 |
| DCA | 0.30 | 0.69 | 0.63 | 0.62 | 0.56 | 0.68 | 0.79 | **0.61** | 0.15 | 0.63 |
| BPE | 1.31 | 4.07 | 3.24 | 2.08 | 2.04 | 2.38 | 1.86 | **2.43** | 0.93 | 2.08 |
| ATT | 1.18 | 0.76 | 2.33 | 1.60 | 1.75 | 2.29 | 2.54 | **1.78** | 0.66 | 1.75 |
| COR | 0.38 | 0.62 | 0.67 | 0.52 | 0.36 | 0.39 | 0.63 | **0.51** | 0.13 | 0.52 |
| ∑_16_ PAHs | **22.02** | **36.03** | **27.80** | **25.09** | **18.18** | **34.16** | **16.64** | **25.70** | 7.48 | 25.09 |

b)

| Compound | 2010 | 2012 | 2013 | 2014 | 2015 | 2016 | 2017 | Mean | STD | Median |
| --- | --- | --- | --- | --- | --- | --- | --- | --- | --- | --- |
| NAP | 3.49 | 1.51 | 3.91 | 6.67 | 2.02 | 2.48 | 2.12 | **3.17** | 1.76 | 2.48 |
| ACY | 0.62 | 0.51 | 0.50 | 0.94 | 0.87 | 0.63 | 0.56 | **0.66** | 0.18 | 0.62 |
| ACE | 0.54 | 0.21 | 0.38 | 0.59 | 0.50 | 0.47 | 0.34 | **0.43** | 0.13 | 0.47 |
| FLN | 1.40 | 1.84 | 1.79 | 2.04 | 1.15 | 0.69 | 0.90 | **1.40** | 0.51 | 1.40 |
| PHE | 14.68 | 13.40 | 7.88 | 15.13 | 10.56 | 7.99 | 7.86 | **11.07** | 3.29 | 10.56 |
| ANT | 1.35 | 0.61 | 0.50 | 1.65 | 1.86 | 1.42 | 1.07 | **1.21** | 0.51 | 1.35 |
| FLT | 32.18 | 20.42 | 18.57 | 45.72 | 50.86 | 30.63 | 26.11 | **32.07** | 12.21 | 30.63 |
| PYR | 26.90 | 13.62 | 14.56 | 36.11 | 40.60 | 24.50 | 19.25 | **25.08** | 10.34 | 24.50 |
| RET | 4.34 | 6.84 | 5.57 | 4.56 | 3.98 | 8.22 | 5.71 | **5.60** | 1.51 | 5.57 |
| BBN | 3.01 | 1.50 | 2.06 | 4.53 | 5.30 | 3.15 | 2.48 | **3.15** | 1.35 | 3.01 |
| BGF | 2.76 | 1.61 | 1.61 | 3.74 | 4.35 | 2.67 | 2.39 | **2.73** | 1.02 | 2.67 |
| CCP | <0.03 | <0.03 | <0.03 | <0.03 | <0.03 | <0.03 | <0.03 | **<0.03** | 0.00 | <0.03 |
| BAA | 14.03 | 7.02 | 8.20 | 21.34 | 25.04 | 11.99 | 10.81 | **14.06** | 6.74 | 11.99 |
| TPH | 3.60 | 2.28 | 2.37 | 5.73 | 5.79 | 3.92 | 3.06 | **3.82** | 1.45 | 3.60 |
| CHR | 14.29 | 7.93 | 9.42 | 21.58 | 25.33 | 12.49 | 11.80 | **14.69** | 6.42 | 12.49 |
| BBF | 28.74 | 17.05 | 20.05 | 43.58 | 43.63 | 24.57 | 22.84 | **28.64** | 10.85 | 24.57 |
| BJF | 9.55 | 4.81 | 5.38 | 15.37 | 15.60 | 8.74 | 6.83 | **9.47** | 4.44 | 8.74 |
| BKF | 9.39 | 5.11 | 6.11 | 13.00 | 14.26 | 7.68 | 6.78 | **8.90** | 3.51 | 7.68 |
| BEP | 14.04 | 9.12 | 9.77 | 17.79 | 19.36 | 10.91 | 11.39 | **13.20** | 4.01 | 11.39 |
| BAP | 21.39 | 10.61 | 13.05 | 28.10 | 32.00 | 17.07 | 13.42 | **19.38** | 8.14 | 17.07 |
| PER | 5.14 | 2.47 | 3.35 | 7.14 | 7.50 | 4.31 | 3.13 | **4.72** | 1.97 | 4.31 |
| INP | 18.75 | 10.98 | 11.83 | 25.82 | 24.49 | 14.62 | 12.08 | **16.94** | 6.19 | 14.62 |
| DBA | 2.66 | 1.72 | 2.00 | 3.83 | 4.03 | 2.82 | 2.50 | **2.80** | 0.86 | 2.66 |
| DCA | 2.28 | 1.50 | 1.36 | 3.02 | 3.38 | 1.78 | 1.90 | **2.17** | 0.77 | 1.90 |
| BPE | 15.85 | 9.86 | 10.24 | 20.27 | 18.58 | 11.25 | 9.67 | **13.67** | 4.48 | 11.25 |
| ATT | 4.26 | 1.89 | 2.36 | 3.27 | 4.49 | 2.22 | 2.65 | **3.02** | 1.02 | 2.65 |
| COR | 6.65 | 3.67 | 3.72 | 6.63 | 4.83 | 2.79 | 2.87 | **4.45** | 1.64 | 3.72 |
| ∑_16_ PAHs | **206.27** | **122.40** | **129.00** | **286.38** | **295.78** | **171.31** | **148.09** | **194.18** | 71.90 | 171.31 |

c)

| Compound | 2006F | 2008S | 2010Sp | 2010F | 2011Sp | 2011F | 2012Sp | 2015S | Mean | STD | Median |
| --- | --- | --- | --- | --- | --- | --- | --- | --- | --- | --- | --- |
| NAP | 1.57 | 0.90 | 3.05 | 2.53 | 5.98 | 3.78 | 2.30 | 3.32 | **2.93** | 1.55 | 2.79 |
| ACY | 0.26 | 0.34 | 0.50 | 0.38 | 0.74 | 0.60 | 0.53 | 0.55 | **0.49** | 0.15 | 0.51 |
| ACE | 0.01 | 0.50 | 0.46 | 0.40 | 0.70 | 0.50 | 0.40 | 0.44 | **0.43** | 0.19 | 0.45 |
| FLN | 0.94 | 0.41 | 0.61 | 0.65 | 1.16 | 0.71 | 0.75 | 0.68 | **0.74** | 0.23 | 0.69 |
| PHE | 13.87 | 5.52 | 7.13 | 7.03 | 11.26 | 8.59 | 7.64 | 6.10 | **8.39** | 2.82 | 7.38 |
| ANT | 0.45 | 1.21 | 0.81 | 0.57 | 1.30 | 1.39 | 0.94 | 1.10 | **0.97** | 0.34 | 1.02 |
| FLT | 10.55 | 28.04 | 23.95 | 22.92 | 29.82 | 30.38 | 27.59 | 24.74 | **24.75** | 6.35 | 26.16 |
| PYR | 6.19 | 23.16 | 19.33 | 17.20 | 23.23 | 25.49 | 22.85 | 20.61 | **19.76** | 6.07 | 21.73 |
| RET | 6.80 | 2.72 | 1.97 | 3.45 | 1.87 | 2.18 | 4.87 | 1.93 | **3.22** | 1.77 | 2.45 |
| BBN | 0.04 | 3.05 | 1.82 | 1.95 | 2.84 | 2.78 | 2.57 | 2.26 | **2.16** | 0.96 | 2.41 |
| BGF | 0.71 | 2.24 | 2.11 | 1.65 | 2.68 | 3.39 | 2.44 | 2.46 | **2.21** | 0.78 | 2.34 |
| CCP | <0.03 | <0.03 | <0.03 | <0.03 | <0.03 | <0.03 | <0.03 | <0.03 | **<0.03** | 0.00 | <0.03 |
| BAA | 2.06 | 11.36 | 8.74 | 8.11 | 13.85 | 14.73 | 11.67 | 10.30 | **10.10** | 3.96 | 10.83 |
| TPH | 0.68 | 2.78 | 2.99 | 2.53 | 3.77 | 3.93 | 3.17 | 3.58 | **2.93** | 1.03 | 3.08 |
| CHR | 2.68 | 11.30 | 9.89 | 8.73 | 14.37 | 16.12 | 12.11 | 12.57 | **10.97** | 4.09 | 11.71 |
| BBF | 5.64 | 21.70 | 20.59 | 18.38 | 29.42 | 31.30 | 24.43 | 25.45 | **22.11** | 7.95 | 23.06 |
| BJF | 2.03 | 6.52 | 5.69 | 6.19 | 9.23 | 11.58 | 7.97 | 9.31 | **7.31** | 2.90 | 7.24 |
| BKF | 1.66 | 6.89 | 6.17 | 5.43 | 9.14 | 9.80 | 7.49 | 7.93 | **6.81** | 2.53 | 7.19 |
| BEP | 3.76 | 11.72 | 10.90 | 9.73 | 13.52 | 15.60 | 11.47 | 13.17 | **11.23** | 3.52 | 11.59 |
| BAP | 3.86 | 16.73 | 12.85 | 11.78 | 20.25 | 23.23 | 16.62 | 18.09 | **15.43** | 5.95 | 16.67 |
| PER | 0.73 | 3.56 | 2.49 | 2.41 | 4.27 | 4.51 | 3.47 | 3.55 | **3.13** | 1.22 | 3.51 |
| INP | 2.99 | 11.95 | 10.95 | 9.34 | 15.75 | 15.98 | 11.75 | 14.15 | **11.61** | 4.19 | 11.85 |
| DBA | 0.62 | 2.02 | 1.69 | 2.23 | 2.47 | 2.96 | 2.37 | 3.35 | **2.22** | 0.83 | 2.30 |
| DCA | 0.48 | 1.32 | 1.84 | 1.43 | 2.64 | 2.29 | 1.89 | 2.09 | **1.75** | 0.67 | 1.86 |
| BPE | 2.55 | 9.97 | 9.93 | 7.75 | 11.32 | 11.71 | 9.15 | 12.53 | **9.36** | 3.14 | 9.95 |
| ATT | 1.64 | 2.56 | 5.77 | 3.06 | 2.32 | 2.64 | 2.13 | 4.40 | **3.07** | 1.36 | 2.60 |
| COR | 0.95 | 1.86 | 2.46 | 1.43 | 2.82 | 2.44 | 1.80 | 3.95 | **2.21** | 0.93 | 2.15 |
| ∑_16_ PAHs | **55.92** | **152.00** | **136.65** | **123.42** | **190.77** | **197.26** | **158.57** | **161.90** | **147.06** | 44.37 | 155.28 |

d)

| Compound | 2006F | 2008S | 2010Sp | 2010F | 2011Sp | 2011F | 2012Sp | 2015S | Mean | STD | Median |
| --- | --- | --- | --- | --- | --- | --- | --- | --- | --- | --- | --- |
| NAP | 1.02 | 1.00 | 1.97 | 2.15 | 1.08 | 1.29 | 0.09 | 2.16 | **1.35** | 0.72 | 1.19 |
| ACY | 0.96 | 0.56 | 0.71 | 0.64 | 0.59 | 0.66 | 3.54 | 0.78 | **1.05** | 1.01 | 0.68 |
| ACE | 0.32 | 0.24 | 0.30 | 0.24 | 0.24 | 0.39 | 0.56 | 0.91 | **0.40** | 0.23 | 0.31 |
| FLN | 0.63 | 0.39 | 0.39 | 1.16 | 0.41 | 0.69 | 2.63 | 1.24 | **0.94** | 0.76 | 0.66 |
| PHE | 10.86 | 5.83 | 6.32 | 10.55 | 5.36 | 8.05 | 7.22 | 16.54 | **8.84** | 3.73 | 7.63 |
| ANT | 1.62 | 1.32 | 1.58 | 0.78 | 0.65 | 1.67 | 1.10 | 3.30 | **1.50** | 0.82 | 1.45 |
| FLT | 38.24 | 28.68 | 30.07 | 20.04 | 16.86 | 31.52 | 24.97 | 56.36 | **30.84** | 12.29 | 29.38 |
| PYR | 32.42 | 25.86 | 25.90 | 14.32 | 14.57 | 29.28 | 21.95 | 46.99 | **26.41** | 10.52 | 25.88 |
| RET | 5.00 | 1.85 | 1.36 | 2.97 | 1.32 | 1.08 | 2.79 | 1.41 | **2.22** | 1.33 | 1.63 |
| BBN | 4.38 | 3.38 | 3.16 | 2.02 | 2.05 | 3.72 | 2.74 | 5.40 | **3.36** | 1.15 | 3.27 |
| BGF | 3.64 | 3.04 | 2.72 | 1.97 | 1.82 | 3.20 | 2.98 | 4.35 | **2.97** | 0.83 | 3.01 |
| CCP | <0.03 | <0.03 | <0.03 | <0.03 | <0.03 | <0.03 | <0.03 | <0.03 | **<0.03** | 0.00 | <0.03 |
| BAA | 20.50 | 21.60 | 18.25 | 10.88 | 11.52 | 24.05 | 16.04 | 28.61 | **18.93** | 6.07 | 19.37 |
| TPH | 4.87 | 4.27 | 4.17 | 2.76 | 2.74 | 4.54 | 3.75 | 6.04 | **4.14** | 1.09 | 4.22 |
| CHR | 19.87 | 18.50 | 15.35 | 10.25 | 10.17 | 20.75 | 15.53 | 25.99 | **17.05** | 5.38 | 17.01 |
| BBF | 36.71 | 33.57 | 26.70 | 19.53 | 22.13 | 37.36 | 26.23 | 43.87 | **30.76** | 8.42 | 30.14 |
| BJF | 12.09 | 11.06 | 9.61 | 5.32 | 6.43 | 10.08 | 9.31 | 14.05 | **9.74** | 2.84 | 9.85 |
| BKF | 12.18 | 11.03 | 8.99 | 6.08 | 6.79 | 11.23 | 8.51 | 14.58 | **9.93** | 2.86 | 10.01 |
| BEP | 20.20 | 17.27 | 15.71 | 11.16 | 12.40 | 17.51 | 14.76 | 21.71 | **16.34** | 3.61 | 16.49 |
| BAP | 30.64 | 27.82 | 22.76 | 15.45 | 17.05 | 28.36 | 22.80 | 35.33 | **25.03** | 6.78 | 25.31 |
| PER | 7.00 | 6.12 | 4.85 | 3.36 | 3.74 | 6.40 | 5.15 | 7.97 | **5.58** | 1.59 | 5.64 |
| INP | 19.39 | 18.07 | 15.89 | 11.56 | 11.32 | 18.17 | 15.19 | 23.15 | **16.59** | 3.98 | 16.98 |
| DBA | 2.78 | 2.66 | 2.25 | 1.73 | 1.51 | 3.25 | 2.67 | 4.62 | **2.68** | 0.97 | 2.66 |
| DCA | 2.31 | 2.39 | 1.92 | 1.48 | 1.56 | 2.15 | 1.62 | 3.65 | **2.14** | 0.70 | 2.04 |
| BPE | 15.58 | 14.75 | 14.58 | 9.34 | 8.94 | 15.17 | 11.89 | 21.70 | **13.99** | 4.07 | 14.66 |
| ATT | 3.96 | 3.63 | 2.19 | 2.20 | 1.86 | 3.08 | 3.72 | 7.34 | **3.50** | 1.74 | 3.35 |
| COR | 2.27 | 2.35 | 2.97 | 1.63 | 1.47 | 2.35 | 2.03 | 5.10 | **2.52** | 1.14 | 2.31 |
| ∑16 PAHs | 243.72 | 211.88 | 192.00 | 134.72 | 129.19 | 231.88 | 180.90 | 326.14 | **206.30** | 63.66 | 201.94 |

**Table S12** PAH concentration in surface soil (sampling depth 10 cm)

| Location | Type of site | Land use | Collection year | PAH concentration [ng g^-1^] | Mean | PAHs | Reference |
| --- | --- | --- | --- | --- | --- | --- | --- |
| Košetice | Background | Grassland | 2010-2017 (except 2011) | 17-296 | 110 | Σ16 EPA | This study |
| Mokrá | Semi-urban | Grassland | 2006- 2015 | 56-326 | 177 | Σ16 EPA | This study |
| Košetice | Background | Different | 1996-2005 | 41-5,600 | 600 | Σ16 EPA | Holoubek et al. 2007 |
| Central and Eastern Europe | Background,  rural, urban, industrial | Not specified | 2005-2006 | 68–58,384 | n.d. | Σ16 EPA | Holoubek et al. 2007 |
| Scotland^a^ | Urban and rural | Different | 2007-2009 | 34-12,560 | 1466 | Σ16 EPA | Rhind et al. 2013 |
| France^b^ | Industrial, urban, suburban, remote | Not specified | 2000 | 450-5,650 | 2510 | Σ14 | Motelay-Massei et al. 2004 |
|  | Remote |  | 2000 | 450-940 | n.d. | Σ14 |  |
| UK (England, Northern Ireland,  Scotland, Wales)^a^ | Rural heritage | Not specified | 2001-2002 | 25-9,230 | 270 | Σ22 | Environmen-tal Agency 2007 |
|  | Rural |  |  | 43-167,502 | 2244 | Σ22 |  |
|  | Urban |  |  | 92-551,000 | 14200 | Σ22 |  |
| Estonia | Urban, rural | Not specified | 1996 | 11-153,000 | n.d. | Σ12 | Trapido 1999 |
|  | Rural |  | 1996 | 11-2240 | ≈100 | Σ12 |  |
| Norway^a^ | Background/remote | Grassland | 1998 | 8.6-1050 | 63 | Σ15 | Nam et al. 2008 |
| UK^a^ |  |  | 1998 | 56-11,200 | 700 | Σ15 |  |
| Europe | Rural | Not specified | Not specified | Median: 300-400 | n.d. | Σ16 | UNEP 2003 |
| Temperate latitudes | Remote | Grassland | 1994 | 63-321 | 142 | Σ20 | Wilcke & Amelung 2000 |
| Czech Republic | Mainly rural | Arable^c^ | 1992-2007 | 140-2,436 | 847 | Σ16 | Holoubek et al. 2009 |
|  |  | Grassland | Not specified | 123-15,284 | 2511 | Σ16 |  |
|  |  | Forest-highland |  | 149–1,435 | 539 | Σ16 |  |
| Czech Republic | Different | Arable | 1997-2009 | 538-933 | n.d. | Σ16 | National Centre for Toxic Compounds 2017 |
|  |  | Grassland | 1997-2009 | 594 – 1,235 | n.d. | Σ16 |  |
|  | Protected area | Non-forest, not distorted | 1997-2009 | 101 – 303 | n.d. | Σ16 |  |

^a^Sampling depth: 0-5 cm ^b^Sampling depth: 3-10 cm ^c^Sampling depth: 0-25 cm

**Table S13** Concentrations of OPAHs and O-heterocycles in soil samples from a) Košetice-1, b) Košetice-2, c) Mokrá-1 and d) Mokrá-2 in ng g^-1^ (F: Fall; Sp: Spring; S:Summer; STD: Standard deviation). Values <LOQ were replaced by LOQ/2 if the detection frequency was >25 % (Fig. S3), else replaced by 0 ng g^-1^

a)

| Compound | 2010 | 2012 | 2013 | 2014 | 2015 | 2016 | 2017 | Mean | STD | Median |
| --- | --- | --- | --- | --- | --- | --- | --- | --- | --- | --- |
| 1,4-O_2_NAP | 0.10 | 0.06 | 0.08 | 0.05 | 0.09 | 0.06 | 0.43 | **0.12** | 0.14 | 0.08 |
| 1-(CHO)NAP | 0.015 | 0.024 | 0.003 | 0.028 | 0.003 | 0.003 | 0.022 | **0.014** | 0.011 | 0.015 |
| DBF | 0.14 | 0.37 | 0.33 | 0.32 | 0.14 | 0.02 | 0.10 | **0.20** | 0.13 | 0.14 |
| 9-OFLN | 0.45 | 0.70 | 0.45 | 0.92 | 0.34 | 0.07 | 0.22 | **0.45** | 0.29 | 0.45 |
| 6-OBCC | 0.07 | 0.24 | 0.07 | 0.72 | 0.07 | 0.07 | 0.09 | **0.19** | 0.24 | 0.07 |
| 9,10-O_2_ANT | <4.7 | <4.7 | <4.7 | <4.7 | <4.7 | <4.7 | 0.19 | **0.03** | 0.07 | 0.19 |
| 9,10-O_2_PHE | <2.8 | <2.8 | <2.8 | <2.8 | <2.8 | <2.8 | <2.8 | <2.8 | 0.00 | <2.8 |
| 11-OBaFLN | 0.12 | 0.16 | 0.19 | 0.09 | 0.06 | 0.05 | 0.22 | **0.13** | 0.07 | 0.12 |
| 11-OBbFLN | 0.13 | 0.14 | 0.16 | 0.07 | 0.13 | 0.11 | 0.23 | **0.14** | 0.05 | 0.13 |
| BAN | 0.04 | 0.03 | 0.09 | 0.03 | 0.05 | 0.08 | 0.15 | **0.06** | 0.04 | 0.05 |
| 7,12-O_2_BAA | 0.13 | 0.12 | 0.12 | 0.06 | 0.08 | 0.07 | 0.15 | **0.10** | 0.03 | 0.12 |
| 5,12-O_2_NAC | 0.04 | 0.04 | 0.06 | 0.02 | 0.05 | 0.05 | 0.06 | **0.05** | 0.01 | 0.05 |
| 6-OBPYR | 0.08 | 0.10 | 0.21 | 0.10 | 0.12 | 0.13 | 0.12 | **0.12** | 0.04 | 0.12 |
| Σ_13_OPAHs and O-heterocycles | 1.32 | 1.98 | 1.77 | 2.40 | 1.12 | 0.71 | 1.98 | **1.61** | 0.59 | 1.77 |
| Σ_11_OPAHs | 1.11 | 1.37 | 1.36 | 1.37 | 0.91 | 0.62 | 1.78 | **1.22** | 0.38 | 1.36 |

b)

| Compound | 2010 | 2012 | 2013 | 2014 | 2015 | 2016 | 2017 | Mean | STD | Median |
| --- | --- | --- | --- | --- | --- | --- | --- | --- | --- | --- |
| 1,4-O_2_NAP | 0.15 | 0.05 | 0.02 | 0.05 | 0.07 | 0.02 | 0.06 | **0.06** | 0.04 | 0.05 |
| 1-(CHO)NAP | 0.069 | 0.046 | 0.011 | 0.038 | 0.023 | 0.007 | 0.041 | **0.033** | 0.021 | 0.038 |
| DBF | 0.91 | 0.66 | 0.68 | 0.80 | 0.39 | 0.14 | 0.24 | **0.55** | 0.29 | 0.66 |
| 9-OFLN | 1.04 | 0.69 | 0.48 | 0.87 | 0.69 | 0.20 | 0.88 | **0.69** | 0.28 | 0.69 |
| 6-OBCC | 0.27 | 0.07 | 0.34 | 0.83 | 0.35 | 0.17 | 0.59 | **0.37** | 0.26 | 0.34 |
| 9,10-O_2_ANT | <4.7 | <4.7 | <4.7 | <4.7 | <4.7 | <4.7 | 4.30 | **0.61** | 1.63 | 0.00 |
| 9,10-O_2_PHE | <2.8 | <2.8 | <2.8 | <2.8 | <2.8 | <2.8 | <2.8 | <2.8 | 0.00 | <2.8 |
| 11-OBaFLN | 1.42 | 0.49 | 0.67 | 0.95 | 1.45 | 0.54 | 0.83 | **0.91** | 0.40 | 0.83 |
| 11-OBbFLN | 1.58 | 0.62 | 0.88 | 0.84 | 1.43 | 0.61 | 0.86 | **0.98** | 0.38 | 0.86 |
| BAN | 0.67 | 0.28 | 0.44 | 0.42 | 0.62 | 0.36 | 0.53 | **0.47** | 0.14 | 0.44 |
| 7,12-O_2_BAA | 1.05 | 0.47 | 0.67 | 0.69 | 0.78 | 0.43 | 0.54 | **0.66** | 0.21 | 0.67 |
| 5,12-O_2_NAC | 0.57 | 0.22 | 0.33 | 0.38 | 0.51 | 0.24 | 0.29 | **0.36** | 0.13 | 0.33 |
| 6-OBPYR | 0.67 | 0.36 | 0.81 | 0.86 | 1.12 | 0.53 | 0.85 | **0.74** | 0.25 | 0.81 |
| Σ_13_OPAHs and O-heterocycles | 8.41 | 3.96 | 5.33 | 6.73 | 7.44 | 3.22 | 10.01 | **6.44** | 2.43 | 6.73 |
| Σ_11_OPAHs | 7.22 | 3.22 | 4.32 | 5.10 | 6.70 | 2.92 | 9.18 | **5.52** | 2.29 | 5.10 |

c)

| Compound | 2006F | 2008S | 2010F | 2011Sp | 2011F | 2012Sp | 2015S | Mean | STD | Median |
| --- | --- | --- | --- | --- | --- | --- | --- | --- | --- | --- |
| 1,4-O_2_NAP | 0.08 | 0.01 | 0.05 | 0.16 | 0.06 | 0.05 | 0.10 | **0.07** | 0.05 | 0.06 |
| 1-(CHO)NAP | 0.049 | 0.003 | 0.023 | 0.045 | 0.011 | 0.014 | 0.048 | **0.028** | 0.019 | 0.023 |
| DBF | 0.92 | 0.09 | 0.33 | 1.05 | 0.28 | 0.44 | 0.37 | **0.50** | 0.35 | 0.37 |
| 9-OFLN | 1.55 | 0.07 | 0.65 | 1.48 | 0.50 | 0.69 | 1.15 | **0.87** | 0.54 | 0.69 |
| 6-OBCC | 0.07 | 0.07 | 0.07 | 0.72 | 0.53 | 0.28 | 0.57 | **0.33** | 0.27 | 0.28 |
| 9,10-O_2_ANT | 1.61 | <4.7 | 1.83 | <4.7 | <4.7 | 1.46 | 1.26 | **0.88** | 0.84 | 1.26 |
| 9,10-O_2_PHE | <2.8 | <2.8 | <2.8 | <2.8 | <2.8 | <2.8 | <2.8 | <2.8 | 0.00 | <2.8 |
| 11-OBaFLN | 0.23 | 0.49 | 0.71 | 0.90 | 1.13 | 0.76 | 1.27 | **0.79** | 0.36 | 0.76 |
| 11-OBbFLN | 0.19 | 0.51 | 0.53 | 0.68 | 1.19 | 0.89 | 1.44 | **0.78** | 0.43 | 0.68 |
| BAN | 0.06 | 0.31 | 0.27 | 0.48 | 0.60 | 0.37 | 0.66 | **0.39** | 0.21 | 0.37 |
| 7,12-O_2_BAA | 0.14 | 0.35 | 0.50 | 0.53 | 0.66 | 0.46 | 1.00 | **0.52** | 0.27 | 0.50 |
| 5,12-O_2_NAC | 0.06 | 0.20 | 0.23 | 0.30 | 0.32 | 0.31 | 0.48 | **0.27** | 0.13 | 0.30 |
| 6-OBPYR | 0.10 | 0.40 | 0.34 | 0.60 | 0.81 | 0.46 | 0.52 | **0.46** | 0.22 | 0.46 |
| Σ_13_OPAHs and O-heterocycles | 5.06 | 2.51 | 5.55 | 6.94 | 6.10 | 6.20 | 8.87 | **5.89** | 1.93 | 6.10 |
| Σ_11_OPAHs | 4.06 | 2.35 | 5.15 | 5.17 | 5.28 | 5.48 | 7.93 | **5.06** | 1.67 | 5.17 |

d)

| Compound | 2006F | 2008S | 2010F | 2011Sp | 2011F | 2012Sp | 2015S | Mean | STD | Median |
| --- | --- | --- | --- | --- | --- | --- | --- | --- | --- | --- |
| 1,4-O_2_NAP | 0.028 | 0.016 | 0.034 | 0.028 | 0.032 | 0.005 | 0.062 | **0.030** | 0.018 | 0.028 |
| 1-(CHO)NAP | 0.019 | 0.003 | 0.058 | 0.014 | 0.009 | 0.003 | 0.015 | **0.017** | 0.019 | 0.014 |
| DBF | 0.31 | 0.13 | 0.59 | 0.24 | 0.23 | 0.09 | 0.09 | **0.24** | 0.17 | 0.23 |
| 9-OFLN | 0.71 | 0.37 | 1.28 | 0.40 | 0.43 | 0.18 | 0.39 | **0.54** | 0.36 | 0.40 |
| 6-OBCC | 0.10 | 0.28 | 0.42 | 0.34 | 0.07 | 0.07 | 0.48 | **0.25** | 0.17 | 0.28 |
| 9,10-O_2_ANT | 0.75 | <4.7 | 5.05 | <4.7 | <4.7 | <4.7 | <4.7 | **0.83** | 1.88 | 0.00 |
| 9,10-O_2_PHE | <2.8 | <2.8 | <2.8 | <2.8 | <2.8 | <2.8 | <2.8 | <2.8 | 0.00 | <2.8 |
| 11-OBaFLN | 1.36 | 0.73 | 1.18 | 0.66 | 1.13 | 1.09 | 1.57 | **1.10** | 0.32 | 1.13 |
| 11-OBbFLN | 1.42 | 0.79 | 0.73 | 0.57 | 0.80 | 0.89 | 2.20 | **1.06** | 0.57 | 0.80 |
| BAN | 0.67 | 0.26 | 0.23 | 0.21 | 0.31 | 0.43 | 0.72 | **0.41** | 0.21 | 0.31 |
| 7,12-O_2_BAA | 0.59 | 0.36 | 0.34 | 0.34 | 0.36 | 0.37 | 0.89 | **0.46** | 0.21 | 0.36 |
| 5,12-O_2_NAC | 0.57 | 0.36 | 0.22 | 0.20 | 0.34 | 0.36 | 0.69 | **0.39** | 0.18 | 0.36 |
| 6-OBPYR | 0.78 | 0.47 | 0.62 | 0.38 | 0.46 | 0.64 | 0.82 | **0.60** | 0.16 | 0.62 |
| Σ_13_OPAHs and O-heterocycles | 7.28 | 3.77 | 10.76 | 3.39 | 4.19 | 4.14 | 7.93 | **5.92** | 2.79 | 4.19 |
| Σ_11_OPAHs | 6.88 | 3.36 | 9.75 | 2.81 | 3.89 | 3.98 | 7.36 | **5.43** | 2.59 | 3.98 |


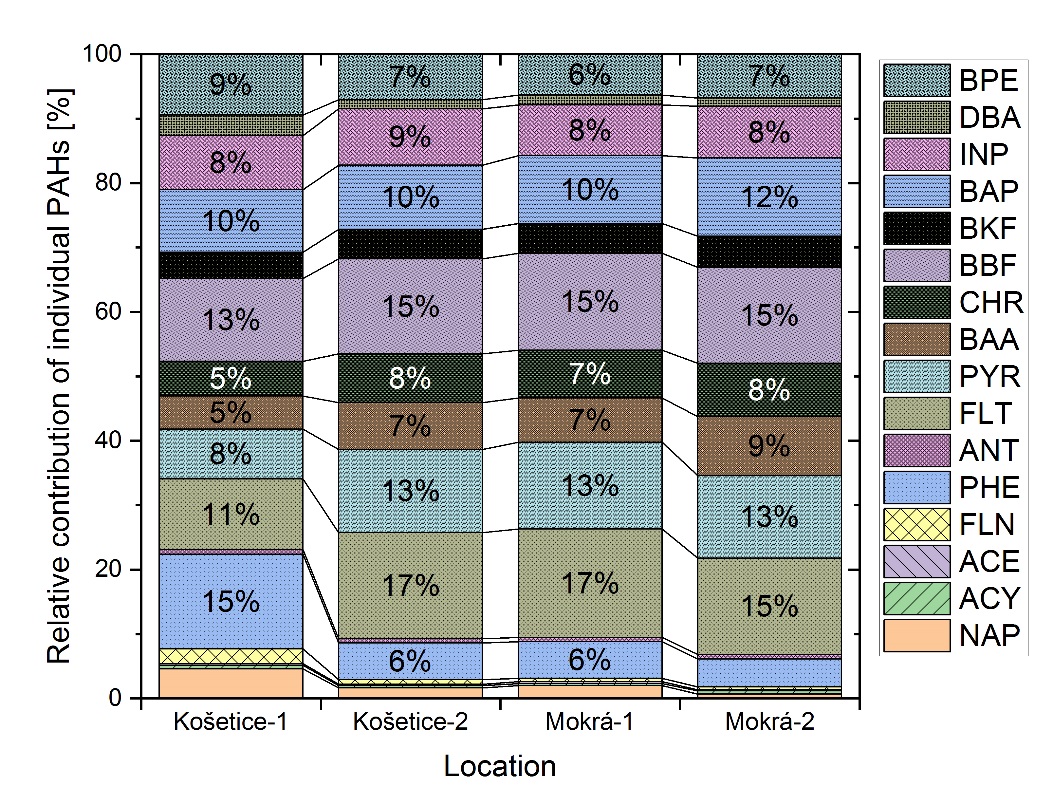


a)


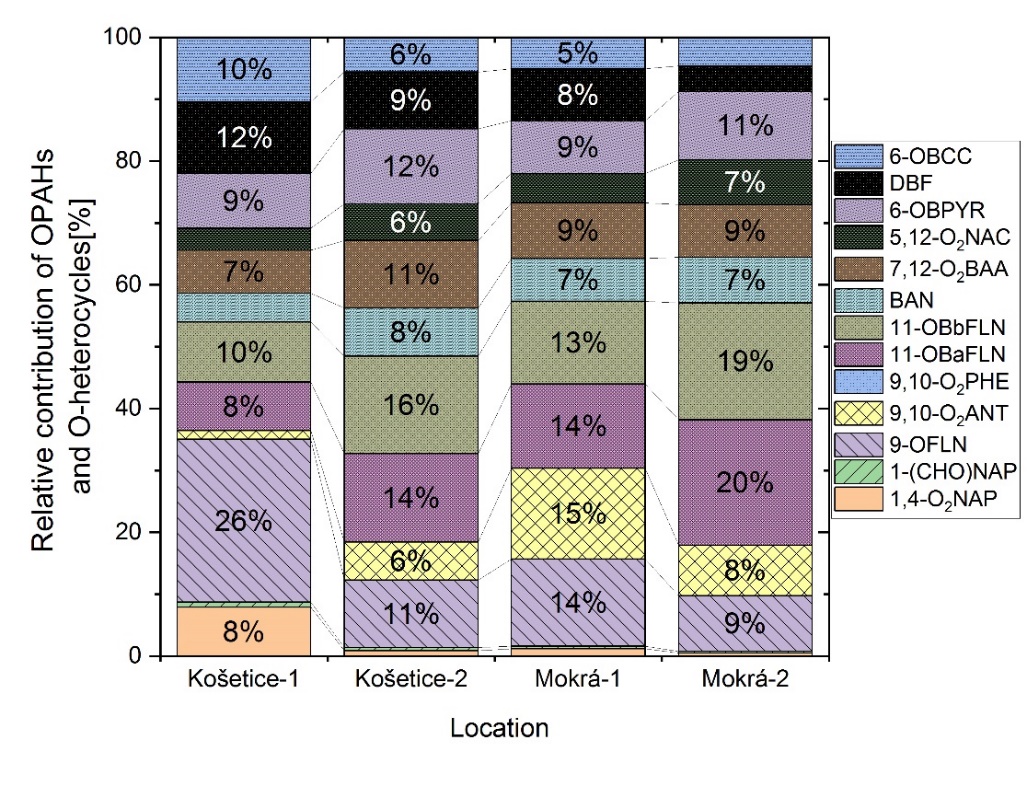

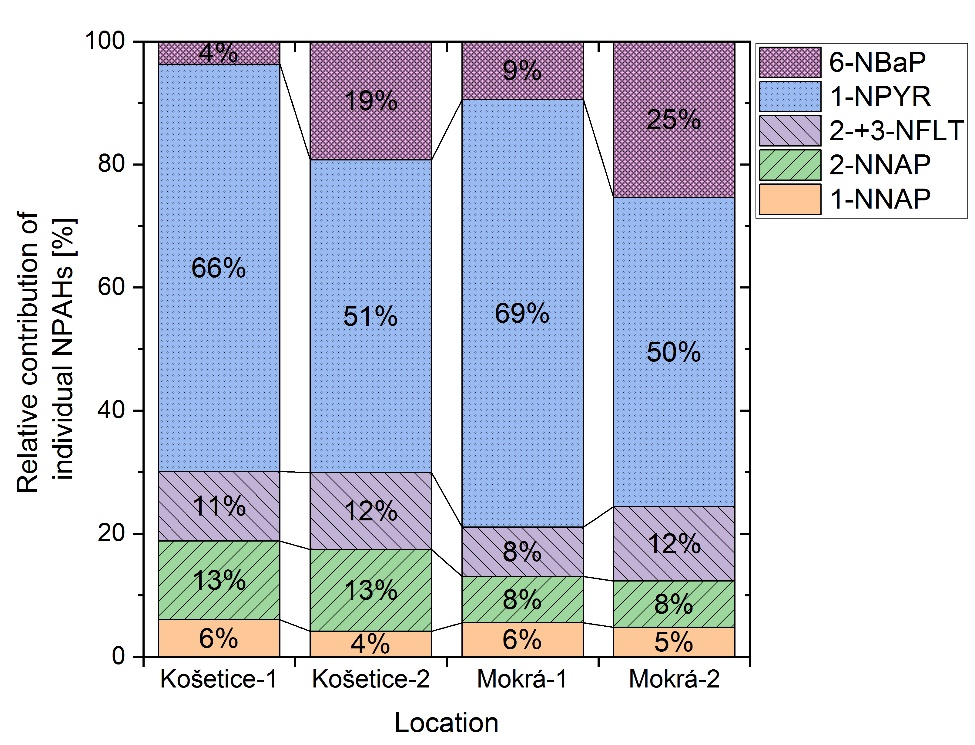


c)

b)

**Fig. S6** Location average of relative concentrations of a) 16 PAHs, b) 11 OPAHs and 2 O-heterocycles and c) 18 NPAHs in soil from Košetice and Mokrá (average of all examined years)


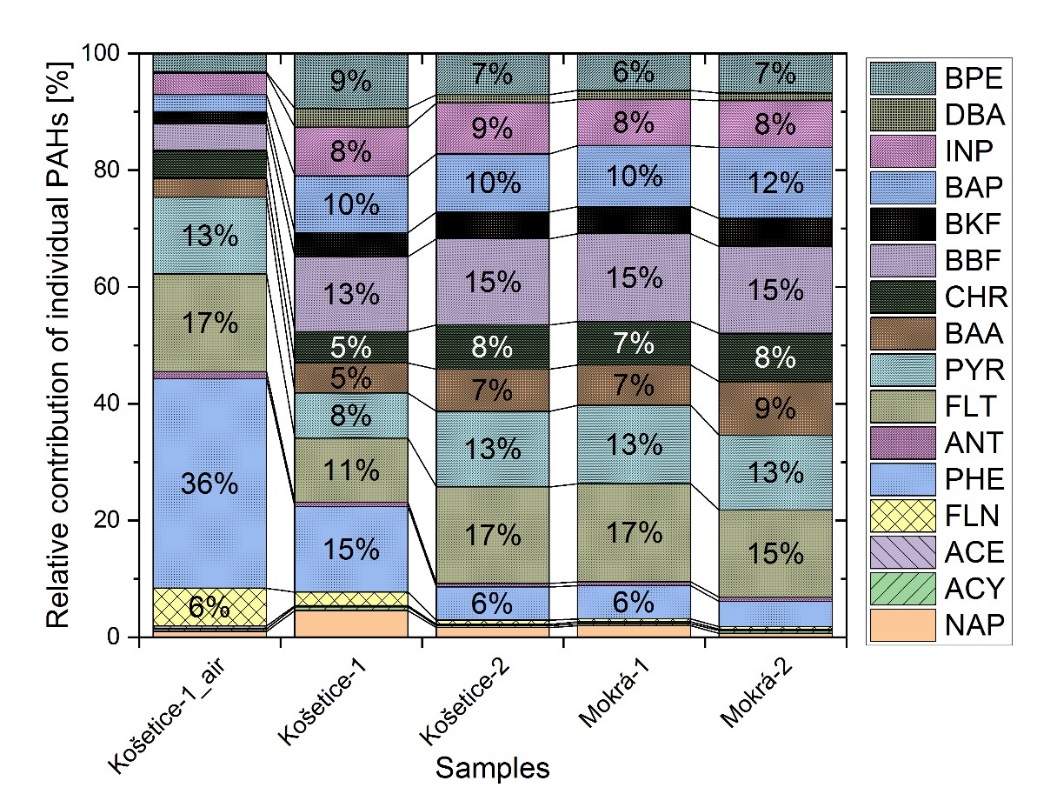


b)

a)


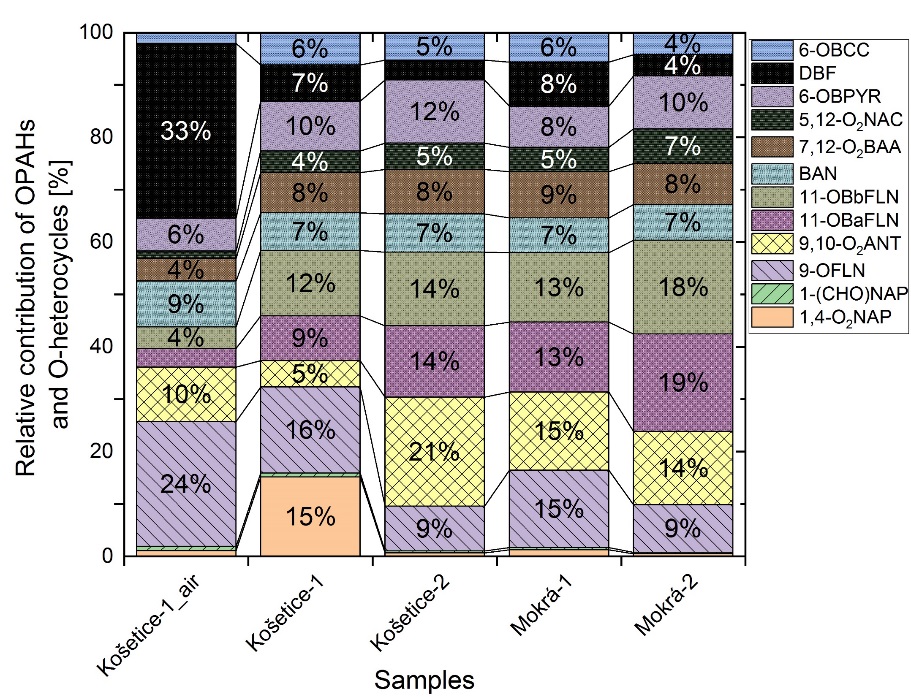


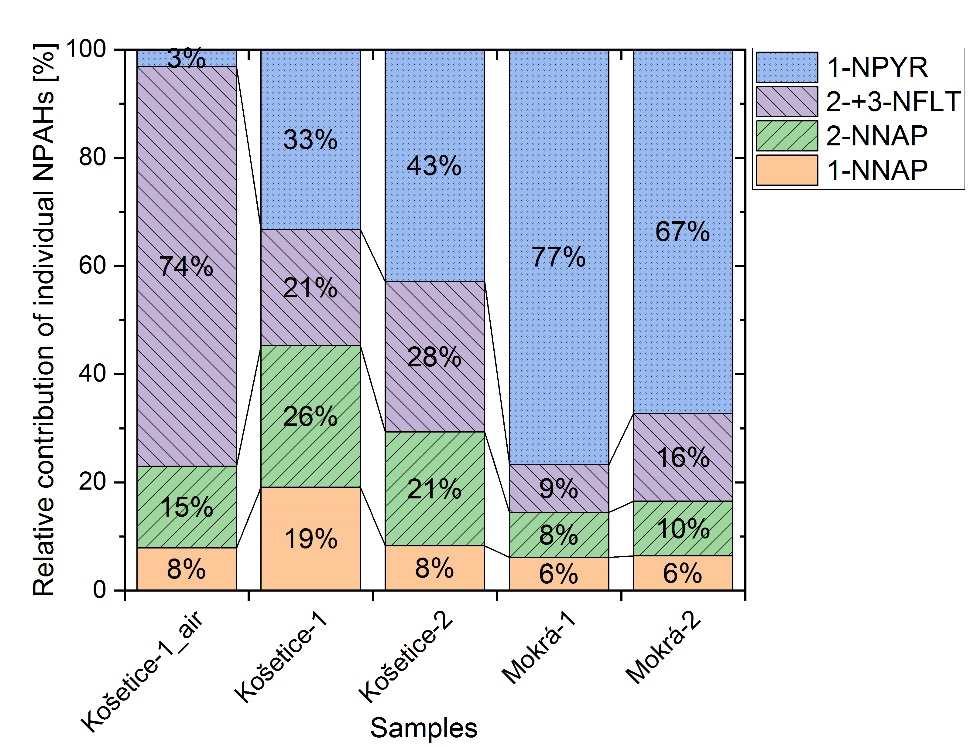


c)

**Fig. S7** Location average of relative contribution of a) 16 PAHs, b) 11 OPAHs and 2 O-heterocycles and c) 18 NPAHs in air at Košetice (taken from Nežiková et al. 2021) and in soil. The relative contributions in soil and air from Košetice show the average of the years 2015-2017, the mean of all examined years is shown for the locations in Mokrá


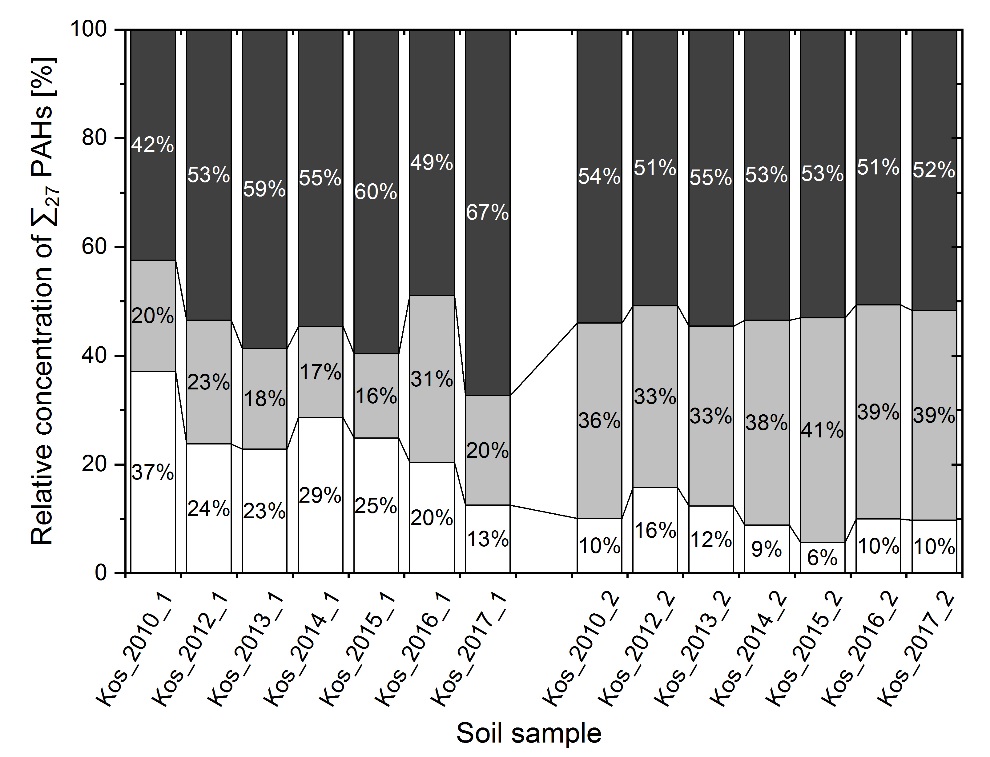

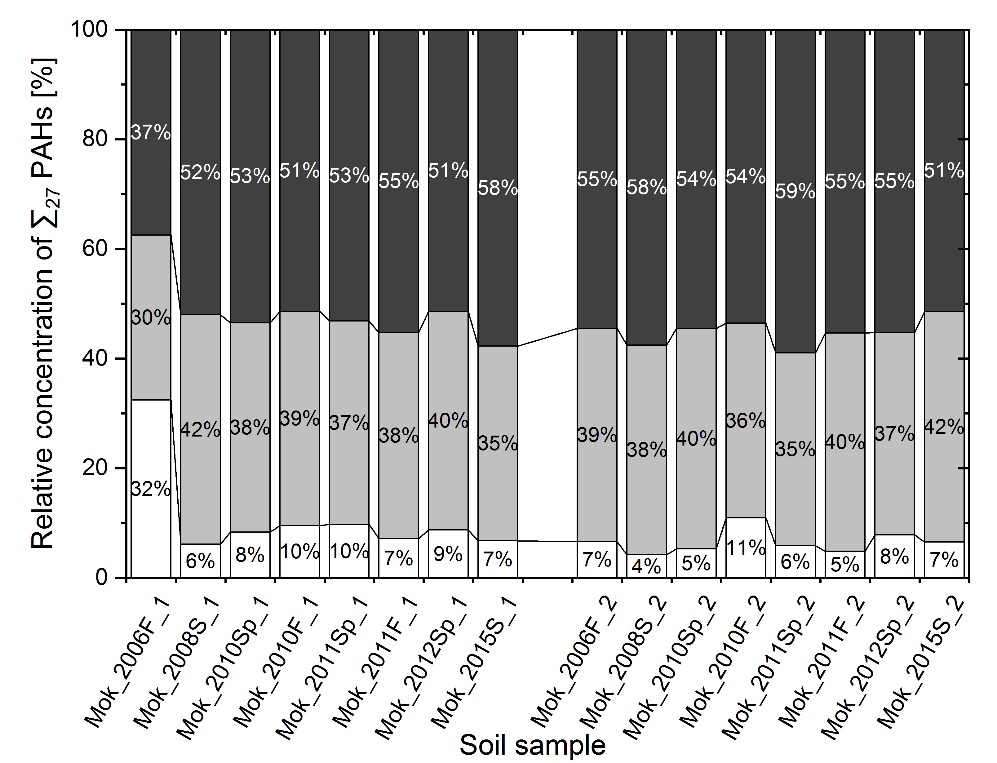


b)

a)

**Fig. S8** Relative concentrations of different ring size PAHs to the ∑_27_PAHs a) in Košetice and b) in Mokrá, split into 2-3-ring PAHs (white), 4 ring PAHs (light grey) and 5-7-ring PAHs (dark grey)

**Table S14** Temperature and precipitation compared to average in Košetice and Mokrá (Czech Hydrometeorological Institute 2005-2017)

| Site | Year | Deviation of average temperature (1981-2010) in winter (Dec/Jan/Feb) [°C] | Precipitation amount as % of the long-term normal (1981-2010) in winter (Dec/Jan/Feb) |
| --- | --- | --- | --- |
| Košetice | 2010 | -1.1 | 108.7 |
|  | 2012 | 0.0 | 121.3 |
|  | 2013 | 0.0 | 139.7 |
|  | 2014 | 2.6 | 46.7 |
|  | 2015 | 2.3 | 77.3 |
|  | 2016 | 3.3 | 85.3 |
|  | 2017 | -0.9 | 69.3 |
| Mokrá | 2006 | -2.0 | 139.3 |
|  | 2008 | 2.0 | 58.7 |
|  | 2010 | -1.1 | 149.0 |
|  | 2011 | -1.1 | 63.7 |
|  | 2012 | 0.0 | 84.3 |
|  | 2015 | 2.3 | 83.7 |


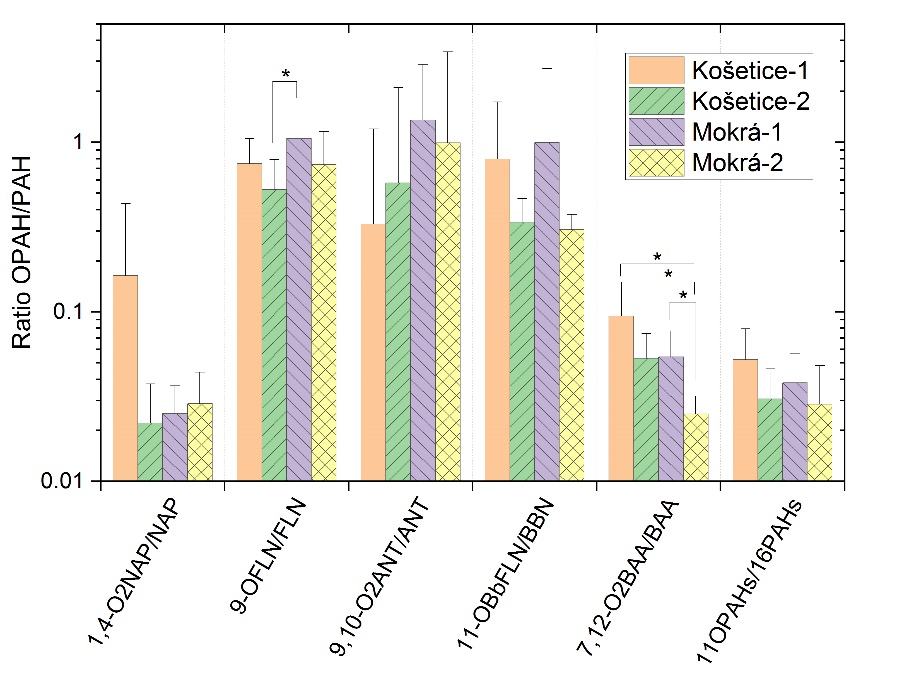


b)

a)


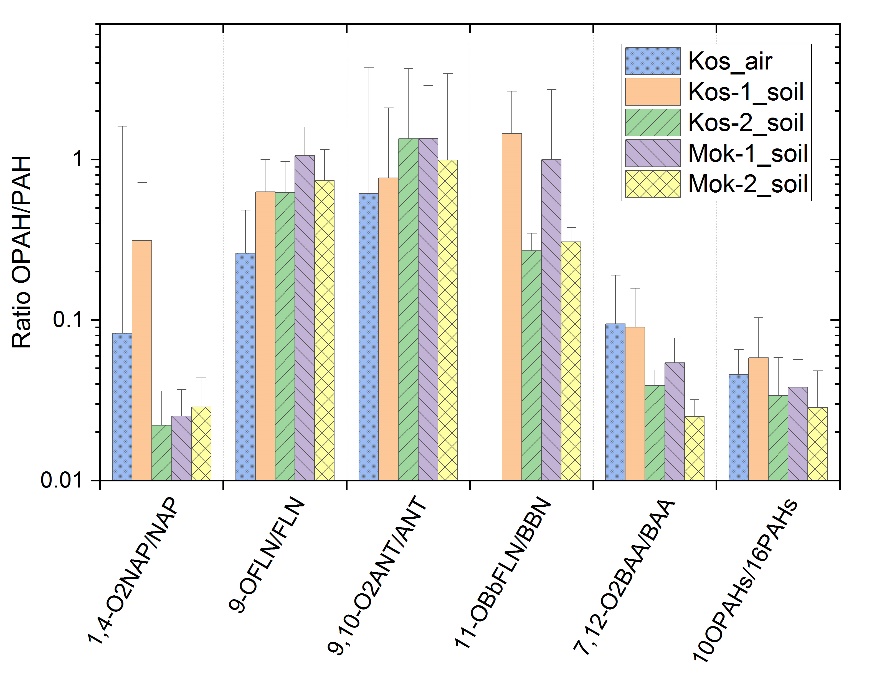


**Fig. S9** Average ratio of OPAHs and corresponding parent PAHs in soil from Košetice and Mokrá a) of all examined years and b) of 2015-2017 at Košetice-1 and -2, of air data from 2015-2017 at Košetice (data from Nežiková et al. 2021) and for all examined years from Mokrá soil. Since BBN was not measured by Nežiková et al., the ratio 11-OBbFLN/BBN is not available for air in Košetice. “*” shows the significance with p<0.05 (Student’s t-test). In b), only the significance between Košetice-1 air and soil was tested but difficult to achieve with only 3 soil samples between 2015-2017. Error bars show the standard deviation of the ratio from different years. Lower limit value for the ratio 9,10-O_2_ANT/ANT since detection frequency of 9,10-O_2_ANT was <25 % (23 %). For calculation: Values <LOQ were replaced by LOQ/2 if the detection frequency was >25 % (**Fig. S3**), else replaced by 0 ng g^-1^. “*” shows the significance with p<0.05 (Student’s t-test) in diagram a).


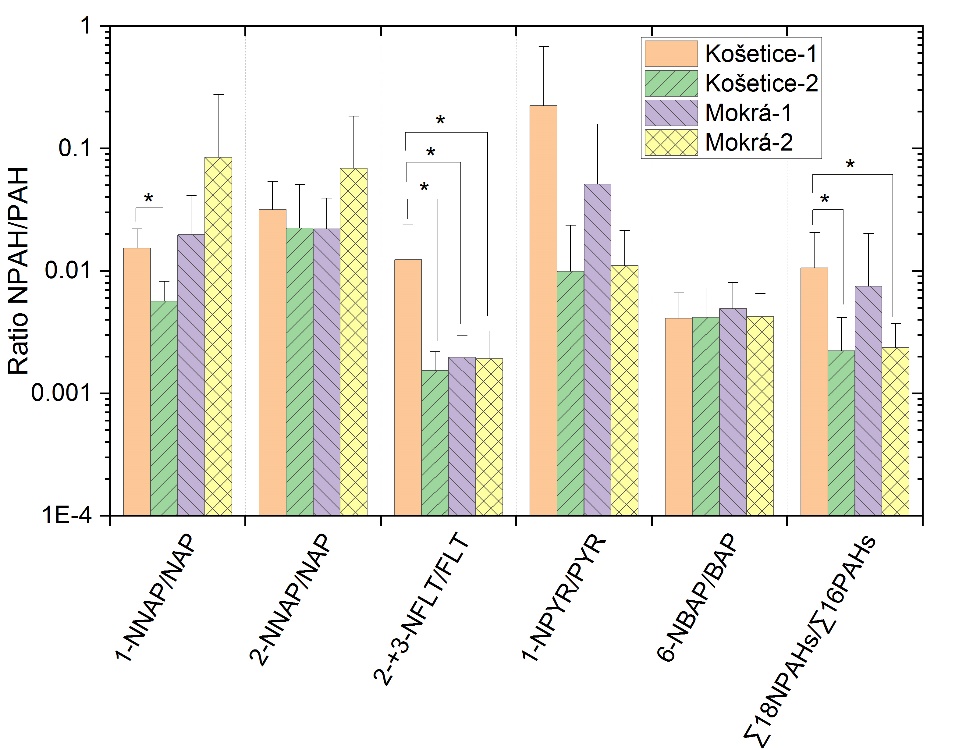


b)

a)


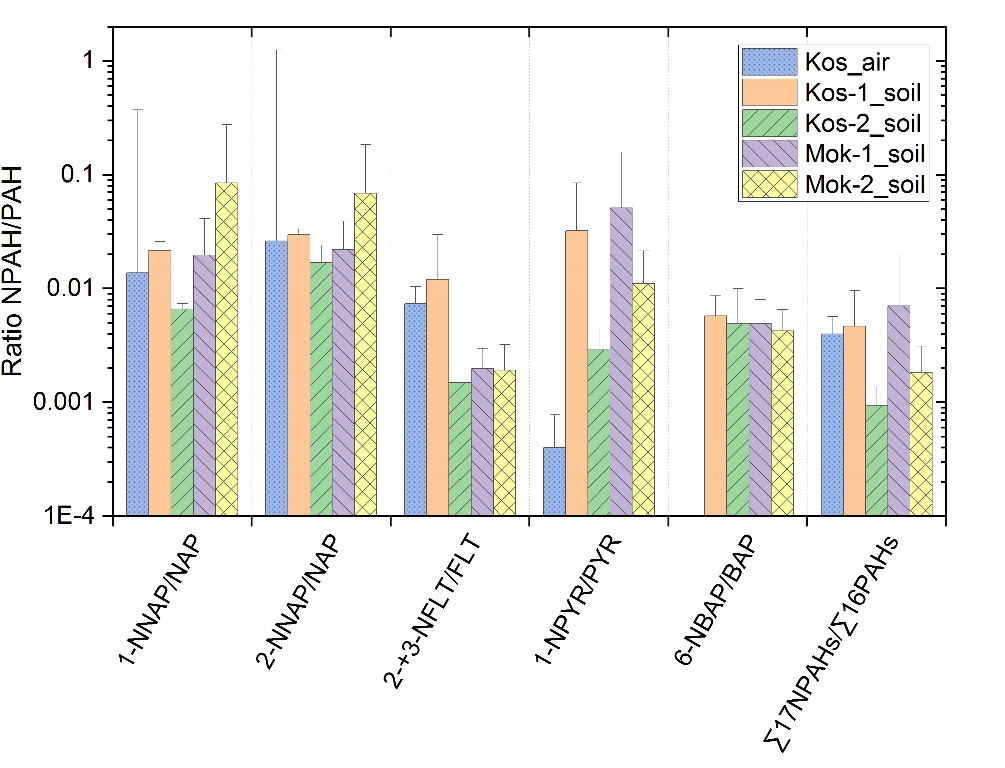


**Fig. S10** Ratio of NPAHs and corresponding parent-PAHs in soil from Košetice and Mokrá a) of all examined years and b) of 2015-2017 at Košetice-1 and Košetice-2, of the air data from 2015-2017 at Košetice (data from Nežiková et al. 2021) and for all examined years from Mokrá soil. Since 6-NBAP was not measured by Nežiková et al., the ratio 6-NBAP/BAP is not available for air in Košetice. Ratios of 1-NNAP/NAP and 2-NNAP/NAP are upper limits, since NNAP values <LOQ were replaced by LOQ/2 (detection frequency >25% i.e., ≈30 %). “*” shows the significance with p<0.05 (Student’s t-test) in diagram a).

**Table S15** OPAH/PAH ratios in soil at different locations; n.d.: not determined

| Location | Košetice,  Czech Republic | Mokrá,  Czech Republic | Manaus,  Brazil | Mainz,  Germany | Berlin,  Germany | Gardsjön,  Sweden | | Gothenburg,  Sweden | Argentina | | China | Bratislava,  Slovakia | | Bangkok,  Thailand | | Yangtze River Delta, China | | Xi'an,  China | | Uzbekistan | |
| --- | --- | --- | --- | --- | --- | --- | --- | --- | --- | --- | --- | --- | --- | --- | --- | --- | --- | --- | --- | --- | --- |
| Type of location | Back-ground | Semi-urban | Back-ground | Urban | Contaminated | Back-ground | | Urban | Remote | | Mixed agri-cultural | Urban | | Urban | | Industrial, agricul-tural | | Sub-urban | | Indus-trial semi-arid | |
| Reference | This study | | Bandowe& Wilcke 2010 | | | | Brorström-Lundén et al. 2010 | | | Wilcke et al. 2014 | Sun et al. 2017 | Bando-we et al. 2011 | Bando-we et al. 2014 | | Cai et al. 2017 | | Wei et al. 2015 | | Bandowe et al. 2010 | |  |
| Soil sampling depth [cm] | 0-10 | 0-10 | 0-10 | 0-10 | 0-10 | Not specified | | | Topsoil (ca. 0-10 or 20) | | 0-20 | topsoil | | 0-5 & 5-10 | | surface | | 0-5 | | 0-10 & 10-20 | |
| TOC [g kg^-1^] | 37 | 28 | 41 | 46 | 18 | n.d. | | n.d. | 4-40 | | n.d. | 5.6-53 | | 4-52 | | n.d. | | 7.92 | | 2-23 | |
| Average temp. [°C] | 9.1 | 8.7 | 27.4 | 11 | 13.1 | 6.5 | | 7.6 | 11.4-18 | | 15-18 | 6.7 | | 28.1 | | 16 | | 14.8 | | 16 | |
| Mean annual precipitation [mm] | 650 | 461 | 2145 | 334 | 570 | 1137 | | 772 | 185-1100 | | 500 | 649 | | 1430 | | 730-1526 | | 528 | | 320-550 | |
| 1,4-O_2_NAP/NAP | 0.04 | 0.02 | <0.06 | <0.04 | 8.67 | n.d. | | n.d. | n.d. | | n.d. | 0.02 | | 0.75 | | n.d. | | 0.13 | | 0.01 | |
| 9-OFLN/FLN | 0.58 | 0.79 | 2.43 | 4.97 | 5089 | 1.80 | | 0.72 | 70.5 | | 0.4 | 5.58 | | 12.20 | | 1.32 | | 4.44 | | 31.00 | |
| 9,10-O_2_ANT/ANT | 0.45^a^ | 1.17^a^ | >8.4 | 6.30 | 11177 | 13.00 | | 3.04 | 2.71-30.0 | | 2.6 | 1.38 | | 7.00 | | 3.50 | | 0.50 | | 29.00 | |
| 11-OBbFLN/BBN | 0.32 | 0.33 | n.d. | n.d. | n.d. | n.d. | | n.d. | n.d. | | n.d. | n.d. | | n.d. | | n.d. | | n.d. | | n.d. | |
| 7,12-O_2_BAA/BAA | 0.05 | 0.03 | n.d. | n.d. | n.d. | 2.33 | | 0.28 | 30.3 | | 0.14 | 0.54 | | 1.90 | | 0.15 | | 0.99 | | n.d. | |

^a^Lower limit value since detection frequency of 9,10-O_2_ANT only 23 %. For calculation: Values <LOQ were replaced by LOQ/2 if the detection frequency was >25 % (**Fig. S3**), else replaced by 0 ng g^-1^

**Table S16** NPAH concentration in surface soil (ng g^-1^) at different locations; n.d. = not determined; <x = smaller than not reported limit

| Location | Košetice, Czech Republic | Mokrá, Czech Republic | Gardsjön, Sweden | Gothenburg, Sweden | Ejby, Denmark | Yangtze River Delta, China | Basel, Switzerland | Hanoi, Vietnam | China plateau | China temperate | China temperate | China subtropical | China tropical |
| --- | --- | --- | --- | --- | --- | --- | --- | --- | --- | --- | --- | --- | --- |
| Type of location | Back-ground | Semi-urban | Back-ground | Urban | Back-ground | Urban | Urban | Urban, traffic | rural | urban | rural | rural | rural |
| Type of soil | Grassland | | Not specified | | Grass-land with cattle grazing | Not speci-fied | Parks & play-grounds | Not speci-fied | Forest, agricultural, river shore, grassland | | | | |
| Reference | This study | | Brorström-Lundén et al. 2010 | | Vikelsøe at al. 2002 | Cai et al. 2017 | Niederer 1998 | Pham et al. 2015 | Bandowe et al. 2019 | | | | |
| Soil sampling depth [cm] | 0-10 | 0-10 | upper 2-3 | upper 2-3 | 0-50 | 0-10 | 0-5 | 0–5, 5–10 & 15–20 | 0-5 | 0-5 | 0-5 | 0-5 | 0-5 |
| TOC [g kg^-1^] | 37 | 28 | n.d. | n.d. | n.d. | n.d. | n.d. | n.d. | 39 | 14 | 19 | 19 | 21 |
| Number of NPAHs | 18 | 18 | 8 | 8 | 3 | 12 | 8 | 10 | n.d. | n.d. | n.d. | n.d. | n.d. |
| Sum of NPAHs [ng g^-1^] | 0.31 | 0.54 | 5.83 | 1.35 | 0.5 | 0.60 | 0.34 | 0.3 | n.d. | n.d. | n.d. | n.d. | n.d. |
| 1-NNAP | 0.015 | 0.028 | n.d. | n.d. | 0.1 | 0.4 | <0.03 | n.d. | 0.1 | 0.8 | 0.2 | 0.2 | 0.1 |
| 2-NNAP | 0.040 | 0.040 | n.d. | n.d. | <x | 1.2 | <0.03 | n.d. | n.d. | n.d. | n.d. | n.d. | n.d. |
| 3-NACE | <0.178 | <0.178 | n.d. | n.d. | n.d. | n.d. | n.d. | n.d. | n.d. | n.d. | n.d. | n.d. | n.d. |
| 5-NACE | <0.010 | <0.010 | n.d. | n.d. | n.d. | <x | 0.03 | n.d. | 0.1 | 0.3 | 0.4 | 0.2 | 0.2 |
| 2-NFLN | <0.002 | <0.002 | n.d. | n.d. | 0.4 | <x | 0.07 | n.d. | 0.1 | 0.1 | 0.2 | 0.1 | 0.1 |
| 9-NANT | <0.049 | <0.049 | 0.55 | 0.13 | n.d. | <x | n.d. | 0.0 | 0.1 | 0.2 | 0.2 | 0.1 | 0.1 |
| Location | **Košetice, Czech Republic** | **Mokrá, Czech Republic** | **Gardsjön, Sweden** | **Gothenburg, Sweden** | **Ejby, Denmark** | **Yangtze River Delta, China** | **Basel, Switzerland** | **Hanoi, Vietnam** | **China plateau** | **China temperate** | **China temperate** | **China subtropical** | **China tropical** |
| 9-NPHE | <0.041 | <0.041 | n.d. | n.d. | n.d. | <x | n.d. | n.d. | 0.1 | 0.4 | 0.4 | 0.3 | 0.2 |
| 3-NPHE | <0.015 | <0.015 | n.d. | n.d. | n.d. | <x | n.d. | n.d. | n.d. | n.d. | n.d. | n.d. | n.d. |
| 2+3-NFLT | 0.037 | 0.052 | 1.7 | 0.50 | n.d. | n.d. | 0.03 | n.d. | 0.6 | 2.1 | 3.9 | 1.8 | 1.3 |
| 1-NPYR | 0.176 | 0.332 | 3.2 | 0.31 | n.d. | 1.6 | 0.15 | 0.0 | 0.3 | 0.6 | 2 | 1.3 | 1.2 |
| 7-NBAA | <0.009 | <0.009 | <0.04 | <0.02 | n.d. | <x | n.d. | 0.02 | n.d. | n.d. | n.d. | n.d. | n.d. |
| 6-NCHR | <0.007 | <0.007 | n.d. | n.d. | n.d. | <x | <0.03 | 0.113 | 0.7 | 0.5 | 0.5 | 0.8 | 2.4 |
| 1,3-N_2_PYR | <0.001 | <0.001 | <0.1 | <0.06 | n.d. | n.d. | n.d. | 0.0007 | n.d. | n.d. | n.d. | n.d. | n.d. |
| 1,6-N_2_PYR | <0.003 | <0.003 | <0.1 | <0.06 | n.d. | n.d. | n.d. | 0.0045 | n.d. | n.d. | n.d. | n.d. | n.d. |
| 1,8-N_2_PYR | <0.003 | <0.003 | n.d. | n.d. | n.d. | n.d. | n.d. | 0.007 | n.d. | n.d. | n.d. | n.d. | n.d. |
| 6-NBaP | 0.039 | 0.084 | n.d. | n.d. | n.d. | <x | n.d. | 0.027 | n.d. | n.d. | n.d. | n.d. | n.d. |
| 3-NBAN | <0.488 | <0.488 | <0.2 | <0.05 | n.d. | n.d. | n.d. | n.d. | n.d. | n.d. | n.d. | n.d. | n.d. |

**Table S17** Sum of 16 EPA-prioritized PAHs, Σ_16_PAH, in soil at Košetice and Mokrá in ng g^-1^ (STD: Standard deviation)

| Site/Location | Year | Mean | STD | Median | Min | Max | Reference |
| --- | --- | --- | --- | --- | --- | --- | --- |
| Mokrá-1 & 2 | 2006-2015^b^ | 177 | 42 | 171 | 56 | 326 | This study |
| Mokrá-1 | 2006-2015^b^ | 147 | 44 | 155 | 56 | 197 | This study |
| Mokrá-2 | 2006-2015^b^ | 206 | 64 | 202 | 129 | 326 | This study |
| Mokrá-1 | 1998-2005 | 89 | 75 | 61 | 27 | 382 | Hofman, RECETOX, unpublished |
| Mokrá-2 | 1998-2005 | 194 | 121 | 148 | 96 | 593 | Hofman, RECETOX, unpublished |
| Košetice-1 & 2 | 2010-2017^a^ | 110 | 100 | 79 | 16 | 295 | This study |
| Košetice-1 | 2010-2017^a^ | 26 | 7 | 25 | 16 | 36 | This study |
| Košetice-2 | 2010-2017^a^ | 194 | 72 | 171 | 122 | 295 | This study |
| Košetice-1 | 1996-2007 | 72 | 25 | 66 | 41 | 116 | Holoubek et al. 2007 + Prokeš et al. 2019 |
| Košetice-2 | 1996-2007 | 146 | 56 | 137 | 80 | 256 | Holoubek et al. 2007 + Prokeš et al. 2019 |
| Košetice: 9 sites including 1 & 2 | 1996-2005 | 600 |  | 280 | 41 | 5400 | Holoubek et al. 2007 |

^a^except 2011 ^b^except 2007, 2009, 2013, 2014

# References

Bandowe, B.A.M., Shukurov, N., Kersten, M., Wilcke, W. (2010). Polycyclic aromatic hydrocarbons (PAHs) and their oxygen-containing derivatives (OPAHs) in soils from the Angren industrial area, Uzbekistan. *Environmental Pollution*, 158, 2888–2899

Bandowe, B.A.M. & Wilcke W. (2010). Analysis of polycyclic aromatic hydrocarbons and their oxygen-containing derivatives and metabolites in soil. *Journal of Environmental Quality*, 39, 1349-1358

Bandowe, B.A.M., Sobocka J., Wilcke W. (2011). Oxygen-containing polycyclic aromatic hydrocarbons (OPAHs) in urban soils of Bratislava, Slovakia: patterns, relation to PAHs and vertical distribution. *Environmental Pollution,* 159, 539–549

Bandowe, B.A.M., Gómez Lueso M., Wilcke W. (2014). Oxygenated polycyclic aromatic hydrocarbons and azaarenes in urban soils: A comparison of a tropical city (Bangkok) with two temperate cities (Bratislava and Gothenburg). *Chemosphere,* 107, 407-414

Bandowe, B.A.M., Leimer, S., Meusel, H., Velescu, A., Dassen, S., Eisenhauer, N., Hoffmann, T., Oelmann, Y., Wilcke, W. (2019). Plant diversity enhances the natural attenuation of polycyclic aromatic compounds (PAHs and oxygenated PAHs) in grassland soils. *Soil Biology and Biochemistry,* 129, 60–70

Brorström-Lundén, E., Remberger, M., Kaj, L., Hansson, K., Palm-Cousins, A., Andersson, H., Haglund, P., Ghebremeskel, M., Schlabach, M. (2010). Results from the Swedish National Screening Programme 2008: screening of unintentionally produced organic contaminants. *Swedish Environmental Research Institute (IVL) report B1944*, Göteborg, Sweden

Cai, C.Y., Li, J.Y., Wu, D., Wang, X.L., Tsang, D.C.W., Li, X.D., Sun, J.T., Zhu, L.Z.,Shen, H.Z., Tao, S., Liu, W.X. (2017). Spatial distribution, emission source and health risk of parent PAHs and derivatives in surface soils from the Yangtze River Delta, eastern China. *Chemosphere,* 178, 301-308Cousins, I.T., Kreibich, H., Hudson, L.E., Lead, W.A., Jones, K.C. (1997). PAHs in soils: contemporary UK data and evidence for potential contamination problems caused by exposure of samples to laboratory air. *The Science of the Total Environment*, 203, 141-156

Czech Hydrometeorological Institute (2005-2017), [https://www.chmi.cz/historicka-data/pocasi/uzemni-teploty?l=en#](https://www.chmi.cz/historicka-data/pocasi/uzemni-teploty?l=en)

Degrendele C., Fiedler H., Kocan A., Kukučka P., Přibylová P., Prokeš R., Klánová J., Lammel G. (2020). Multiyear levels of PCDD/Fs, dl-PCBs and PAHs in background air in central Europe and implications for deposition. *Chemosphere*, 240, 124852

Hollender, J., Koch, B., Lutermann, C, Dott, W. (2002). Efficiency of different methods and solvents for the extraction of polycyclic aromatic hydrocarbons from soils. *International Journal of Environmental Analytical Chemistry*, 83, 21–32

Holoubek, I., Klánová, J., Jarkovský, J., Kubík, V., Helešic, J. (2007). Trends in background levels of persistent organic pollutants at Košetice observatory, Czech Republic. Part II. Aquatic and terrestrial environments 1996–2005. *Journal of Environmental* *Monitoring,* 9, 564–571

Holoubek, I., Dušek, L., Sáňka, M., Hofman, J., Čupra, P., Jarkovský, J., Zbíral, J., Klánová, J. (2009). Soil burdens of persistent organic pollutants – Their levels, fate and risk. Part I. Variation of concentration ranges according to different soil uses and locations. *Environmental Pollution*, 157, 3207–3217

Kalina, J., Scheringer, M., Borůvková, J., Kukučka, P., Přibylová, P., Bohlin-Nizzetto, P., Klánova, J. (2017). Passive air samplers as a tool for assessing long-term trends in atmospheric concentrations of semivolatile organic compounds, *Environmental Science and Technology*, 51, 7047-7054

Lau, E.V., Gan, S., Ng, H.K. (2010). Extraction techniques for polycyclic aromatic hydrocarbons in soils. *International journal of analytical chemistry*, 2010, 1-9

Lhotka, R., Pokorná, P., Zíková, N.L. (2019). Long-term trends in PAH concentrations and sources at rural background site in Central Europe. *Atmosphere,* 10, 687

MacLeod, C.J.A. & Semple, K.T., 2003. Sequential extraction of low concentrations of pyrene and formation of non-extractable residues in sterile and non-sterile soils. *Soil Biology & Biochemistry*, 35, 1443–1450

Motelay-Massei, A., Ollivon, D., Garban, B., Teil, M.J., Blanchard, M., Chevreuil, M.,2004. Distribution and spatial trends of PAHs and PCBs in soils in the Seine River basin, France. *Chemosphere,* 55, 555-565

Nam, J.J., Thomas, G.O., Jaward, F.M., Steinnes, E., Gustafsson, O., Jones, K.C. (2008). PAHs in background soils from Western Europe: influence of atmospheric deposition and soil organic matter. *Chemosphere,* 70, 1596-1602

National Centre for Toxic Compounds (2017). Czech Republic updated national implementation plan for the Stockholm Convention on persistent organic pollutants in the period 2012 – 2017.

Nežiková, B., Degrendele, C., Bandowe, B.A.M., Holubová Šmejkalová, A., Kukučka, P., Martiník, J., Prokeš, R., Přibylová, P., Klánová, J., Lammel, G. (2021). Atmospheric concentrations of nitrated and oxygenated polycyclic aromatic hydrocarbons and oxygen heterocycles are declining in Central Europe, *Chemosphere*, 269, 128738

Niederer, M. (1998). Determination of polycyclic aromatic hydrocarbons and substitutes (nitro-, oxy-PAHs) in urban soil and airborne particulate by GCMS and NCI-MS/MS. *Environmental Science and Pollution Research,* 5, 209-216

Pham, C.T., Tang, N., Toriba, A. (2015). Polycyclic aromatic hydrocarbons and nitropolycyclic aromatic hydrocarbons in atmospheric particles and soil at a traffic site in Hanoi, Vietnam. *Polycyclic Aromatic Compounds*, 35, 353-371

Prokeš, R., Příbylová, P., Borůvková, J., Audy, O., Martiník, J., Vinkler, J., Klánová, J., Holoubek, I. (2019). 30 years of integrated POPs monitoring at National Atmospheric Observatory Kosetice, Ovzduší – 14^th^ Czech-Slovak Meeting on Air Pollution, Brno, Czech Republic, 16.-17.4.2019 (published as: Ovzduší 2019 Program a Sborník Konference, Report Masaryk University, ISBN 978-80-210-6203-0, Brno, Czech Republic, pp. 36-37)

Rhind, S.M., Kyle, C.E., Kerr, C., Osprey, M., Zhang, Z.L., Duff, E.I., Lilly, A., Nolan, A., Hudson, G., Towers, W., Bell, J., Coull, M., McKenzie, C. (2013). Concentrations and geographic distribution of selected organic pollutants in Scottish surface soils. *Environmental Pollution,* 182, 15–27

Sun Z., Zhu Y., Zhuo S.J., Liu W.P., Zeng E.Y., Wang X.L., Xing B.S., Tao S. (2017). Occurrence of nitro- and oxy-PAHs in agricultural soils in eastern China and excess lifetime cancer risks from human exposure through soil ingestion. *Environment International*, 108, 261-270

Tomaz, S., Shahpoury, P., Jaffrezo, J.L., Lammel, G., Perraudin, E., Villenave, E., Albinet, A. (2016). One-year study of polycyclic aromatic compounds at an urban site in Grenoble (France): Seasonal variations, gas/particle partitioning and cancer risk estimation. *Science of the Total Environment*, 565, 1071–1083

UBA (2003). Überprüfung von Methoden des Anhanges 1 der Bundesbodenschutz- und Altlastenverordnung (BBodSchV) zur Beurteilung der Bodenqualität. *Umweltbundesamt report* #FB 000397 20174240 (*Texte* 37/03), Federal Environment Agency, Dessau, Germany

UNEP (2003). Global Report 2003 Regionally based assessment of POPs

USEPA (2007), Environment Agency, UKSHS Report No. 9 PAHs soil

USEPA (2019). Estimation Programs Interface Suite™ for Microsoft® Windows, v 4.11. United States Environmental Protection Agency, Washington, USA

Trapido, M. (1999). Polycyclic aromatic hydrocarbons in Estonian soil: contamination and profiles. *Environmental Pollution*, 105, 67-74

Vikelsøe, J., Thomsen, M., Carlsen, L., Johansen, E. (2002). Persistent organic pollutants in soil, sludge and sediment. A multianalytical field study of selected organic chlorinated and brominated compounds. National Environmental Research Institute, Denmark. *NERI Technical Report* No. 402, 100 pp. URL: <http://technical-reports.dmu.dk>

Wei, C., Bandowe, B.A.M., Han, Y., Cao, J., Zhan, C., Wilcke, W. (2015). Polycyclic aromatic hydrocarbons (PAHs) and their derivatives (alkyl-PAHs, oxygenated-PAHs, nitrated-PAHs and azaarenes) in urban road dusts from Xi’an, Central China. *Chemosphere,* 134, 512–520

Wilcke, W. (2000). Synopsis polycyclic aromatic hydrocarbons (PAHs) in soil - a review. *Journal of Plant Nutrition and Soil Science*, 163, 229-248

Wilcke, W., Amelung, W. (2000). Persistent organic pollutants (POPs) in native grassland soils along a climosequence in North America. *Soil Science Society of America Journal*, 64, 2140–2148

Wilcke, W., Bandowe, B.A.M., Gomez Lueso, M., Ruppenthal, M. del Valle, H. Oelmann, Y. (2014a). Polycyclic aromatic hydrocarbons (PAHs) and their polar derivatives (oxygenated PAHs, azaarenes) in soils along a climosequence in Argentina. *Science of the Total Environment,* 473–474, 317–325
